# *In Silico* Characterization and Phylogenetic Distribution of Extracellular Matrix Components in the Model Rhizobacteria *Pseudomonas fluorescens* F113 and Other Pseudomonads

**DOI:** 10.3390/microorganisms8111740

**Published:** 2020-11-06

**Authors:** Esther Blanco-Romero, Daniel Garrido-Sanz, Rafael Rivilla, Miguel Redondo-Nieto, Marta Martín

**Affiliations:** Departamento de Biología, Facultad de Ciencias, Universidad Autónoma de Madrid, c/Darwin 2, 28049 Madrid, Spain; esther.blanco@uam.es (E.B.-R.); daniel.garrido@uam.es (D.G.-S.); rafael.rivilla@uam.es (R.R.); miguel.redondo@uam.es (M.R.-N.)

**Keywords:** extracellular matrix, *Pseudomonas*, *Pseudomonas fluorescens* F113, phylogenetic, biofilm, *Pseudomonas* acidic polysaccharide

## Abstract

Biofilms are complex structures that are crucial during host–bacteria interaction and colonization. Bacteria within biofilms are surrounded by an extracellular matrix (ECM) typically composed of proteins, polysaccharides, lipids, and DNA. Pseudomonads contain a variety of ECM components, some of which have been extensively characterized. However, neither the ECM composition of plant-associated pseudomonads nor their phylogenetic distribution within the genus has been so thoroughly studied. In this work, we use in silico methods to describe the ECM composition of *Pseudomonas fluorescens* F113, a plant growth-promoting rhizobacteria and model for rhizosphere colonization. These components include the polysaccharides alginate, poly-N-acetyl-glucosamine (PNAG) and levan; the adhesins LapA, MapA and PsmE; and the functional amyloids in *Pseudomonas*. Interestingly, we identified novel components: the *Pseudomonas* acidic polysaccharide (Pap), whose presence is limited within the genus; and a novel type of Flp/Tad pilus, partially different from the one described in *P. aeruginosa*. Furthermore, we explored the phylogenetic distribution of the most relevant ECM components in nearly 600 complete *Pseudomonas* genomes. Our analyses show that *Pseudomonas* populations contain a diverse set of gene/gene clusters potentially involved in the formation of their ECMs, showing certain commensal versus pathogen lifestyle specialization.

## 1. Introduction

The genus *Pseudomonas* is remarkably diverse, with more than 200 reported species thus far [1,2,3,4]. Strains belonging to this genus are ubiquitous and have a versatile metabolism [5]. This diversity is also reflected in the different mechanisms they display for environmental adaption. Among these traits, biofilm formation is a common bacterial lifestyle strategy essential during niche and host colonization. Bacteria exhibit numerous ways to adhere to solid surfaces, at the air–liquid interface, and to cells [6]. Bacterial biofilms are complex structures typically embedded in a self-produced extracellular matrix (ECM), which in turn is composed of exopolysaccharides (EPS), proteins, lipids, and extracellular DNA [7]. Members of the genus *Pseudomonas* contain a significant and diverse array of components forming the ECM [8]. Some of them are reportedly widespread in the genus, such as the exopolysaccharide alginate [9,10,11,12,13,14] or functional amyloids in *Pseudomonas* (Fap) [15]. However, most of the ECM components have only been described in certain taxonomic groups and seem to be specialized according to the bacteria niche and host interactions these groups display.

Several pseudomonad species have been extensively characterized regarding biofilm formation such as the opportunistic pathogen *Pseudomonas aeruginosa* [16] or plant-associated species, mainly from the *P. fluorescens, P. putida*, and *P. syringae* groups [17]. Among the matrix components used by *P. aeruginosa* for biofilm formation, alginate, polysaccharide synthesis locus (Psl) and pellicle locus (Pel) exopolysaccharides, and the adhesin CdrA have been extensively studied [18,19,20,21,22,23,24].

On the other hand, plant-interacting *Pseudomonas* can produce many different types of EPSs and adhesive proteins during biofilm formation. *P. protegens* genomes contain the *psl* and/or *pel* gene clusters involved in EMC formation through exopolysaccharide production, whereas *P. syringae* genomes contain the *pel* gene cluster but not the *psl* cluster [17]. However, *P. fluorescens*, *P. putida,* and *P. stutzeri* do not possess complete *psl* nor *pel* clusters in their genomes [17]. In these species, distinct biofilm-related polysaccharides have also been described. For instance, exopolysaccharides A and B (Pea and Peb, respectively) are important ECM components in *P. putida* [25]. In the *P. fluorescens* group of species (henceforth Pfl group), cellulose [26] and also the poly-N-acetyl-glucosamine (PNAG) in non-cellulose-producing strains [27] have been described as key ECM components. Finally, levan (a polyfructan neutral polysaccharide) has also been described in *P. syringae* and certain Pfl group strains [10,28,29].

Regarding adhesive proteins, they can be divided into non-fimbrial or fimbrial based on their structure. Non-fimbrial adhesins include calcium-dependent adhesins belonging to the repeats-in-toxin (RTX) family. These megaproteins are exported through type I secretion systems and are key elements in the formation of biofilms [30]. In this sense, the large adhesion protein A (LapA) is recognized as a key RTX adhesin involved in the attachment to surfaces, including plants, in certain species belonging to *P. putida* and *P. fluorescens* groups [31,32]. Besides LapA, *P. putida* KT2440 also produces another extracellular adhesin named large adhesion protein F (LapF), being both necessary during attachment and rhizosphere colonization [33,34]. Recently, another extracellular protein, the medium adhesion protein A (MapA), has been described in *P. fluorescens* Pf0-1 as a participant in biofilm formation [35]. On the other hand, fimbrial adhesins that have been characterized in the *Pseudomonas* genus with a role in biofilm formation include proteinaceous structures such as the type IV pili [36,37] or Fap [38].

In recent years, multilocus sequence and whole-genome analyses of the genus *Pseudomonas* have allowed establishing the existence of several groups of closely related species, including *P. aeruginosa*, *P. stutzeri*, *P. putida*, *P. syringae*, *P. asplenii,* and the Pfl group [1,3]. Likewise, the Pfl group can be subdivided into nine phylogenetic subgroups (SGs), *P. protegens*, *P. chlororaphis*, *P. corrugata*, *P. koreensis*, *P. jessenii*, *P. mandelii*, *P. fragi*, *P. gessardii,* and *P. fluorescens* [3,39]. Bacteria belonging to the genus *Pseudomonas* have the potential to produce several plant growth-promoting traits, including plant hormones, antibiotics, or compounds involved in the solubilization of nutrients [40,41,42,43]. The Pfl group encompasses bacteria that are great colonizers of the rhizosphere and known to produce a wide array of compounds or activities helping plant physiology and growth, such as the synthesis of antimicrobial compounds against phytopathogens or plant hormones [44,45,46]. *Pseudomonas fluorescens* F113 (hereafter F113) is a plant growth-promoting rhizobacteria (PGPR) and a model for rhizosphere colonization that belongs to the *P. corrugata* SG [39]. However, the mechanisms that this bacterium uses to achieve biofilm formation are still poorly characterized.

Due to the importance of members of this genus from a pathogenic and biotechnological perspective, increasing the knowledge of the mechanisms involved in bacteria–host interaction such as biofilm formation is crucial. The aim of this study was to identify the potential ECM components encoded in the F113 genome and to assess the phylogenetic distribution of the most relevant ECM components present in pseudomonads among all the sequenced *Pseudomonas* available to date.

## 2. Materials and Methods

### 2.1. Gene and Protein Analysis in Pseudomonas fluorescens F113

*P. fluorescens* F113 gene clusters related to ECM components were analyzed using the “*Pseudomonas* Genome Database” [47] and Blastn [48]. Gene maps were represented at scale using the “genoPlotR” R package version 0.8.9 [49]. Operon prediction was achieved using the Database of Prokaryotic Operons (DOOR) 2.0 tool [50]. The predicted functions of gene products were assessed by searches of homologs with known function in other organisms using the “*Pseudomonas* Genome Database” and Blastp [48], excluding *Pseudomonadales* in the case of the newly described components (Supplementary Table S1). The families of glycosyltransferases (GTs) encoded in the gene clusters were retrieved from the Carbohydrate-active enzymes (CAZy) database [51]. Protein domains were identified and analyzed in PHMMER [52], the Pfam database [53], and SMART [54]. Potential transmembrane regions were predicted by TMHMM2 server v. 2.0 [55], PHMMER, and SMART. Protein structures were studied with Phyre^2^ [56] intensive mode unless stated otherwise, and SwissModel [57]. For prediction of the protein subcellular localization, the PSORTb v.3.0.2 (based on CMSVM, CytoSVM, and SCL-BLAST) [58] and CELLO v.2.5 [59] tools were used.

### 2.2. Datasets

All sequenced and complete *Pseudomonas* genomes, proteomes, and annotations, with a level of completion of “chromosome”, were downloaded from the NCBI ftp server, RefSeq database [60] on 24 February 2020. Genomes with no taxonomic classification at the species level were excluded. Duplicated strain genomes were also removed, maintaining those with lower number of contigs. The resulting proteomes of a total of 611 *Pseudomonas* were used for further analysis and are listed in Supplementary Table S2.

### 2.3. Identification of Orthologous Groups and Core-Protein Phylogeny

Orthologous groups (OGs) among all *Pseudomonas* genomes were identified using all-vs-all amino acid searches within the OrthoFinder software, version 2.3.3 [61], using DIAMOND version 0.9.30.131 [62]. The resulting set of 149 single-copy orthologous sequences present in all the genomes was aligned using Clustal Omega version 1.2.4 [63] and further concatenated prior to phylogenetic analysis using the Perl script “catfasta2phyml” [64]. Poorly aligned columns and divergent regions were removed using Gblocks version 0.91b [65], applying a minimum block length of 2 and allowing gap positions in all sequences. A phylogenetic tree was built using the maximum-likelihood (ML) phylogenetic inference implemented in RAxML-HPC (v.8.2.12) [66] via the CIPRES Science Gateway tool [67], using the LG model of amino acid evolution [68], the gamma-distributed substitution rates, and empirical amino acid frequencies. Fast bootstrapping with the subsequent search of the best tree [69] and the autoMRE criterion to determine the number of replicates [70] were applied. The tree was visualized and exported using MEGAX [71], rooted on the midpoint.

### 2.4. Phylogenetic Distribution of Extracellular Components

The distribution of the ECM components among the different *Pseudomonas* phylogenetic groups was examined based on the OGs previously identified. Phylogenetic groups represented by a limited number of proteomes and proteomes not clustered within any phylogenetic group were excluded. Presence/absence data was represented using “ggplot2” R package version 3.2.1 [72].

### 2.5. Phylogeny of Flp/Tad and Pap Matrix Components

The OGs of six fimbrial low-molecular-weight (Flp)/tight adherence protein (Tad) type A, six Flp/Tad type B, and nine *Pseudomonas* acidic polysaccharide (Pap) proteins were used to construct phylogenetic trees. Amino acid sequences from the OGs of these proteins were aligned, concatenated, and processed as described above. The order of concatenation was TadZG, PprB, FppA, TadF, and PprA for Flp/Tad type A matrix component; TadV, PSF113-4189, TadEGZ, and CpaE-like for Flp/Tad type B; and PapDEFHIJLMO for the Pap matrix component. The resulting filtered and concatenated alignments were used to infer the phylogenetic trees of Flp/Tad type A, Flp/Tad type B, and Pap matrix components, using the same methods and parameters specified above.

### 2.6. Synteny of Flp/Tad Clusters

Synteny of the *flp-tad* cluster was based on GenBank annotations (accessed on July 2020) and represented with “genoPlotR” package version 0.8.9. Protein homology was analyzed using Blastp.

## 3. Results and Discussion

### 3.1. Gene Clusters Involved in the Production of Extracellular Matrix Components in Pseudomonas fluorescens F113

According to sequence-based predictions, the genome of the model rhizobacteria *Pseudomonas fluorescens* F113 contains nine gene clusters putatively involved in the synthesis of ECM components. The in silico analyses carried out in this work identified that four of these gene/gene clusters are putatively involved in the synthesis of polysaccharides while the remaining five are likely implicated in the production of extracellular proteins or proteinaceous structures.

#### 3.1.1. Polysaccharides

**Figure 1:**
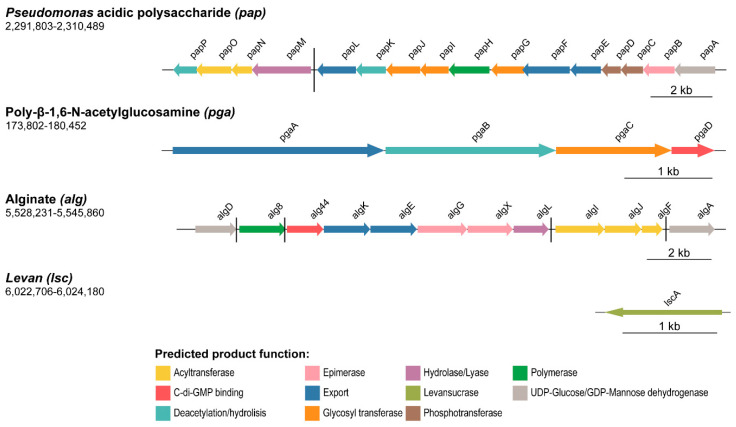
Polysaccharide biosynthetic clusters organization in *P. fluorescens* F113 genome. Schematic representation of the four genes/gene clusters likely associated with polysaccharide production in F113. Genes are scaled and colored according to the putative function of their encoded proteins, as shown in the legend. Arrow direction indicates the orientation of the gene. The name for each gene is represented above the corresponding arrow. Genomic coordinates are shown below the polysaccharide (gene cluster) name. Black horizontal bars depict different transcriptional regulatory units.

Four of the identified gene/gene clusters in *P. fluorescens* F113 encode proteins likely involved in the biosynthesis of polysaccharides: PNAG, encoded by *pgaABCD* (PSF113_0161-0164); *Pseudomonas* acidic polysaccharide (Pap), encoded by *papABCDEFGHIJKLMNOP* (PSF113_1955-1970); alginate, encoded by *alg* (PSF113_4752-4763); and levan, encoded by *lscA* (PSF113_5195, Figure 1).

The *pga* gene cluster contains four putative open reading frames (ORFs, *pgaABCD*), most likely forming a single transcriptional unit, as shown in Figure 1. Their gene products show high homology (around 70%) to proteins that have been shown previously to be involved in the synthesis and export system of the PNAG exopolysaccharide from *Achromobacter* spp. and *Paraburkholderia* spp. [73,74] (Supplementary Table S1). PgaA is a porin involved in the export of the synthesized polysaccharide to the extracellular space. PgaB is a periplasmic carbohydrate esterase involved in the polysaccharide deacetylation by catalyzing the hydrolysis of the N-linked acetyl group from the GlcNAc residues. Finally, PgaC and PgaD are cytoplasmic membrane proteins, being the catalytic synthase (GT-2 family member) and the regulatory subunits, respectively. The *pga* cluster was previously described as a biofilm component involved in cell-cell attachment in *P. fluorescens* SBW25 [27] and is present in other species of pseudomonads with the same genetic organization as in F113. The sequence identity of F113 Pga proteins compared with orthologs within the genus ranges from 70% to more than 99%.

In addition, here we describe a novel polysaccharide synthesis locus designated *pap* (from *Pseudomonas* acidic polysaccharide), which covers 18 Kb and is composed of 16 putative ORFs (*papABCDEFGHIJKLMNOP*), as illustrated in Figure 1. According to the DOOR prediction tool, the *pap* cluster is divided into two different operons: *papA-L* and *papM-P*. We have found that the complete Pap biosynthetic pathway is only present within the *Pseudomonas* genus (homology with proteins outside the *Pseudomonas* genus is only found for PapA-D, Supplementary Table S1) and shares limited homology with proteins involved in the synthesis of other known polysaccharides. Thereby, the *pap* cluster is putatively involved in the synthesis of a novel polysaccharide in *Pseudomonas*. Further analyses have been done to predict the function of Pap proteins and to decipher the biosynthetic pathway of this polysaccharide, which are addressed in the following section.

On the other hand, the *alg* gene cluster is composed of 12 ORFs (PSF113_4752-4763) with five putative operons, as shown in Figure 1. These genes have been recently described as responsible for the synthesis of alginate in the closely related strain *Pseudomonas corrugata* CFBP 5454 [75]. Syntenic organization of the *alg* gene cluster is maintained in F113 and *P. corrugata* CFBP 5454, and the ORFs are almost identical, ranging from 88.1% to 98% of sequence identity (Supplementary Table S3). F113 *alg* gene cluster also has similarity with the alginate encoding cluster from *P. aeruginosa* PAO1 [24], with sequence identities ranging from 71–85%. The sequence identity between F113 and *P. aeruginosa* PAO1 alginate biosynthetic proteins ranges from 43–77% (Supplementary Table S3). Alginate plays a crucial role in biofilm formation in *P. aeruginosa* mucoid strains [76].

Finally, the F113 genome contains the gene PSF113_5195 (Figure 1), which encodes a putative levansucrase implicated in the synthesis of levan, an extracellular polysaccharide composed of sucrose [77,78]. Levansucrases from *P. syringae* pv. *tomato* DC3000 (Lsc3) and *P. chlororaphis* subsp. *aurantica* (LscA) have been characterized and shown to be able to polymerize levan from sucrose and fructooligosaccharides of different lengths [79] with a role in nutrient storage during biofilm formation [10]. The F113 orthologue is 93.4% identical in sequence to LscA, therefore, we have named the F113 gene as *lscA*.

#### 3.1.2. Extracellular Proteins and Proteinaceous Structures

**Figure 2:**
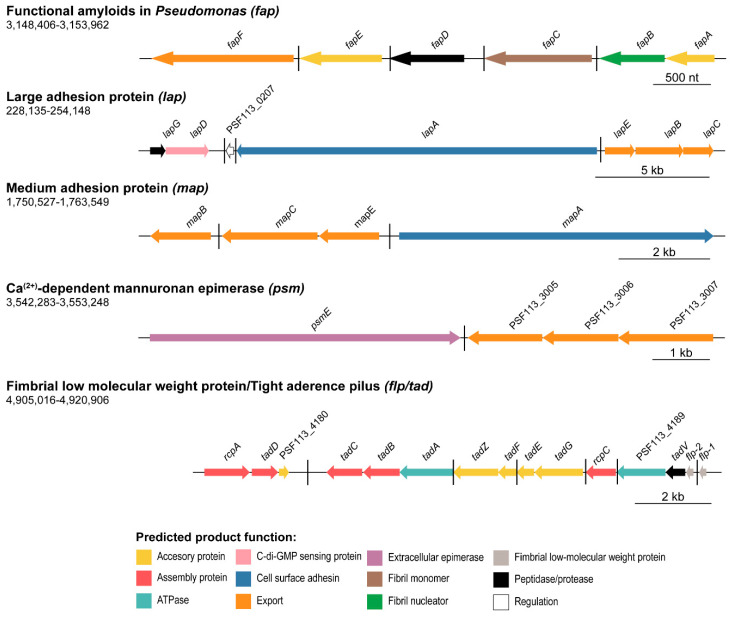
Protein or proteinaceous structure-encoding clusters organization in *P. fluorescens* F113 genome. Schematic representation of the five putative extracellular protein or proteinaceous structure-encoding clusters in F113. Genes are scaled and colored according to the putative function of their encoded proteins as shown in the legends. Arrow direction indicates the orientation of the gene. The name for each gene is represented above the corresponding arrow. Genomic coordinates are shown below the polysaccharide (gene cluster) name. Black horizontal bars depict different transcriptional regulatory units.

Five gene clusters that encode extracellular proteins or proteinaceous structures likely related to the formation of the ECM and their associated transport systems have been identified in the genome of F113 (Figure 2). These clusters include genes putatively encoding the production and secretion of the large LapA adhesin system (PSF113_0205-PSF113_0211), the MapA system (PSF113_1508-1510), and a putative adhesin/extracellular epimerase (PsmE, PSF113_3005-3007). The other two clusters are predicted to encode the functional amyloids in *Pseudomonas* (Fap) system (PSF113_2680-PSF113_2685) and a fimbrial low-molecular-weight protein (Flp)/tight adherence (Tad) pilus (PSF113_4178-PSF113_4192).

Concerning adhesive proteins, the genome of F113 encodes genes likely involved in the synthesis of both fimbrial and non-fimbrial adhesins. The *lap* cluster in F113 is composed of seven ORFs, as observed in Figure 2. The *lapA* gene encodes the large RTX adhesin LapA, the *lapBCE* operon encodes the associated ABC type I secretion system, and the *lapDG* operon encodes the regulatory system. This system was originally described in the closely related strain *P. fluorescens* WCS365 with a role in the transition from reversible to irreversible attachment [32] and whose genetic organization is identical to F113. The sequence identity of the F113 *lapA* ORF compared to the *P. fluorescens* WCS365 LapA is 93%. The other ORFs in the system share a sequence identity of 94–99% (Supplementary Table S3). High amino acid sequence identity can also be observed between F113 and *P. fluorescens* WCS365 Lap proteins (92.3% for LapA and ranging from 97.5–99.5% in the rest of the proteins, Supplementary Table S3).

As shown in Figure 2, the genome of *P. fluorescens* F113 also encodes another RTX-like adhesin system similar to the Lap system (protein sequence identity ranging from 45.8–61%), and that has been recently described as a biofilm component in *P. fluorescens* Pf0-1 [35]: the medium adhesion protein A (MapA). The genetic region also includes the *mapBCE* genes encoding the type I ABC transporter, both in *P. fluorescens* Pf0-1 and F113. MapA proteins from F113 and *P. fluorescens* Pf-01 share 70% sequence identity, and the associated transport systems around an 88–92% protein sequence identity (Supplementary Table S3). The analysis of conserved domains (SMART and Pfam hidden Markov models (HMM); Supplementary Figure S1) revealed that MapA presents four domains: a typical cell-adhesion IG domain, a cadherin-like domain, a Von Willebrand factor type A domain, and an RTX calcium-binding nonapeptide repeats domain. MapA protein from F113 has very low structural homology to other known proteins (identity < 30%, alignment coverage < 7% with Phyre^2^ normal mode).

Another large extracellular protein encoded by the F113 genome is PsmE (PSF113_3004). The *psmE* gene is followed by genes encoding its putative associated type I secretion system (PSF113_3005-PSF113_3007), probably forming a single cluster, as shown in Figure 2. According to the protein domain analysis (SMART and Pfam HMM, Supplementary Figure S1), PsmE contains six domains: a parallel beta-helix (Pbh1), an immunoglobulin-like (IG), an RTX calcium-binding nonapeptide repeat, a pectate lyase, a peptidase M10 serralysin C-terminal, and dystroglycan-type cadherin-like domains. The function of this protein is unknown although the presence of an IG and a cadherin-like domain could imply some adhesive properties. Moreover, the *psmE* nucleotide sequence shares 75% of sequence identity (66% alignment coverage) with *P. syringae* pv. *tomato* DC3000 *psmE* gene (PSPTO_4084, Supplementary Table S3). The protein PsmE from *P. syringae* pv. *tomato* DC3000 has been described as a mannuronan C-5-epimerase and O-acetylhydrolase whose function is related to the modification of alginate by forming guluronic acid (G)-blocks that may be essential for producing stronger gels [80]. Furthermore, at the structural level, the F113 PsmE protein shares certain similarity (60% identity, 20% alignment coverage) with the extracellular mannuronan C-5 epimerase AlgE4 module 2 from *Azotobacter vinelandii*, according to Phyre^2^ and SwissModel, which is an MG block-forming enzyme that catalyzes the alternate epimerization of β-D-mannuronic acid (M) to α-L-guluronic acid (G) in alginate [81].

The *fap* cluster contains six ORFs (*fapA-F*, PSF113_2685-2680) and five predicted operons, as shown in Figure 2. The encoded proteins are putatively involved in the synthesis of amyloids as previously demonstrated in other pseudomonads species [82] and other 14 proteobacterial genera [38]. The relevance of amyloid proteins for structuring biofilms and its biogenesis in pseudomonads have also been analyzed [15], showing that FapC is the major fiber subunit and FapF the exporter. The F113 *fap* genes show the same genetic organization as their homologs in other pseudomonads. However, homology with the Fap proteins of other pseudomonads is highly variable. For instance, the sequence identity of the F113 FapC protein with FapC proteins from strains belonging to the Pfl group ranges from less than 70% to more than 99%. Differences with other groups are even higher. The F113 FapC shows only 43% identity with the FapC proteins from different strains of *P. aeruginosa* (Supplementary Table S3).

The last predicted proteinaceous structure encoded in the F113 genome is the Flp/Tad pilus, a special type IVb pilus structure considered a fimbrial adhesin [30]. The *flp/tad* locus is a large gene cluster composed of 15 ORFs (PSF113_4178-4192) organized in seven putative transcriptional units (Figure 2). This locus has been described in a variety of *Bacteria* and *Archaea* [36], where it plays an important role in colonization. In pseudomonads, an Flp/Tad system has been described in *P. aeruginosa* [83], although half of the Flp/Tad proteins encoded in its genome share no homology with the ones found in F113, as we will detail in the following sections. The comparison of the *flp/tad* cluster of F113 with other bacteria revealed that it is composed of the genes that encode the major and minor fimbrial components (*flp-1* and *flp-2*, respectively), a peptidase (*tadV*), ATPases (PSF113_4189 and *tadA*), assembly proteins (*rcpC*, *rcpA*, and *tadBCD*), and several accessory proteins (*tadGEFZ* and PSF113_4180). There is high redundancy in functions in the *flp/tad* operon with several accessory, assembly, and ATPases encoded proteins.

### 3.2. In Silico Description of the Novel Pap Polysaccharide

As mentioned above, the genome of F113 contains the *pap* gene cluster (*papABCDEFGHIJKLMNOP*), that encodes the proteins necessary for the biosynthesis of a novel polysaccharide. Sequence and structural homology analyses have been used to predict the function of the Pap biosynthetic proteins. Domains, predicted localization, and function for Pap proteins are summarized in Table 1.

PapA is a putative uridine diphosphate (UDP)-glucose (UDP-Glc)/guanidine diphosphate (GDP)-mannose dehydrogenase, probably implicated in the transformation of UDP-Glc/GDP-mannose into UDP-glucuronic (UDP-GlcA)/GDP-mannuronic acid. Therefore, the predicted polysaccharide would have an acidic nature. For this reason, we have named it Pap (*Pseudomonas* Acidic Polysaccharide). PapA is paralog to the GDP-mannose dehydrogenase AlgD (32.7% identity, 83% alignment coverage) from F113. Structural analysis of PapA using Phyre^2^ revealed a high similarity (46–55% identity, 96–97% alignment coverage) to the UDP-glucose-6-dehydrogenases UgdG from *Sphingomonas elodea* and BceC from *Burkholderia cepacia* involved in NAD-dependent 2-fold oxidation of UDP-Glc to UDP-GlcA, which is a key step in gellan [84] and cepacian polysaccharides biosynthesis [85], respectively. Therefore, functional predictions suggest that the most probable residue formed by PapA is UDP-GlcA.

PapB is a putative NAD-dependent epimerase/dehydratase that could catalyze the reversible transformation of UDP-N-acetylglucosamine (UDP-GlcNAc)/UDP-Glc/UDP-glucuronate into UDP-N-acetylgalactosamine/UDP-galactose (UDP-Gal)/UDP-L-iduronate. Structural analysis revealed similarity with the galactose mutarotase/UDP-Gal 4-epimerase Gal10 protein from *Saccharomyces cerevisiae*, which is able to transform UDP-Gal to UDP-Glc [86]; and with the GalE protein from *Burkholderia pseudomallei* (24% identity, 94% alignment coverage), a UDP-Glc 4-epimerase which catalyzes the opposite reaction [87]. The structural and functional predictions suggest two plausible scenarios: PapB as an enzyme that modifies the UDP-GlcA putatively synthesized by PapA or as a protein whose function takes place immediately before PapA in the pathway, providing UDP-Glc/UDP-Gal for the synthesis of the polysaccharide.

PapC is predicted to be an undecaprenyl-phosphate galactose phosphotransferase (PT), located at the inner membrane, that converts UDP-Gal into α-D-galactosyl-diphosphoundecaprenol. PapC is ortholog to the GTs PslA (40.68% identity, 33% alignment coverage) and WbaP (43.37% identity, 77% alignment coverage) from *P. aeruginosa* PAO1 with a role in the Psl and Wzy-capsule biosynthetic pathways respectively [24,88]. These proteins are involved in the transport and polymerization of the polysaccharides, by accepting activated precursors subunits from a GT and their subsequent transfer to an isoprenoid carrier in the inner membrane. Thus, Pap polymers are also likely built on an isoprenoid lipid carrier from where they are assembled and then exported via the action of polymerases, flippases, and export proteins in the outer membrane as occurs in the synthesis of these related polysaccharides [24,89]. Furthermore, PapC is structurally similar to PglC from *Campylobacter concisus* (37% identity, 77% coverage) involved in N-linked glycan synthesis [90].

PapD/EpsD is a putative tyrosine-kinase protein from the CpsD/CapB family with implications in capsular polysaccharide synthesis in other bacteria such as *Streptococcus pneumoniae* [91], which shows structural similarity to Wzc/ETK tyrosine-kinase (21% identity, 98% alignment coverage) from *E. coli*, involved in the polymerization and export processes of capsular polysaccharide biosynthesis [92].

PapE is a multidomain protein placed in the inner membrane with a predicted domain and structure that suggests its involvement in polysaccharide export. For instance, the soluble ligand-binding β-grasp (SLBB) domain (Pfam PF10531) in the C-terminal region has been found in proteins such as Wza with a role in the export of group 1 capsule, including capsule and colonic acid production in *E. coli* [88,93]. Moreover, it is structurally similar to the translocon protein Wza (18% identity, 89% alignment coverage,) from the *Escherichia coli* capsule biosynthetic pathway [94,95].

PapF is a putative inner membrane regulator of the sugar nucleotides chain length due to the presence of a chain length determinant protein (Wzz) domain (Pfam PF02706). Wzz is also a component of the Wzy-capsule synthesis system [96]. This protein shows low structural homology with other known proteins. PapH is a protein that contains an O-antigen ligase (Wzy-C) domain (Pfam PF04932) found in polymerases and therefore could be responsible for the linkage of the sugar nucleotides units giving rise to a long-chain polysaccharide. The protein presents structural similarity with the peptidoglycan polymerase RodA (21% identity, 77% alignment coverage) from the Gram-negative *Thermus thermophilus*.

Additionally, three different GTs encoded in the *pap* cluster were identified, PapG (GT family 4), I, and J (GT family 2). They likely use the sugar nucleotides synthesized by the enzymes encoded in this cluster as substrate, such as the UDP-Gal, UDP-GlcNAc, or UDP-L-iduronate. These GTs present low structural homology with other known proteins (≤ 15% identity).

PapK is predicted to be a deacetylase of unknown function with low structural homology compared to the deacetylase PgaB from *E. coli* (19% identity, 93% alignment coverage). PapL is a membrane protein, and together with PapMN, are uncharacterized proteins with low sequence and structure homology with known proteins. PapL structure shares low homology with the lipid II flippase MurJ from *Escherichia coli* (17% identity, 94% alignment coverage). Thus, PapL could be involved in the formation of a flippase-like system. PapM is slightly similar to the β-glucuronidase from *Acidobacterium capsulatum* (21% identity, 47% alignment coverage [97]. Last, PapN shares limited structural homology to the acetyltransferase YncA from *Salmonella typhimurium* (18% identity, 74% alignment coverage).

PapO is a Gcn5-related N-acetyltransferase (GNAT) family protein that could have a role in the acetylation of the synthesized polysaccharide. This protein shares low homology compared to the FemX GNAT (12% identity, 84% alignment coverage) involved in the cell wall formation of *Weissella viridescens* [98]. The last protein encoded in the *pap* cluster is PapP that resembles a cellobiose PT YdjC-like (Pfam PF04794) protein with a putative role in deacetylation. Structural analysis reveals that PapP is similar to YdjC-like glycoside hydrolase/deacetylase from the Gram-positive *Enterococcus faecalis* (23% identity, 98% alignment coverage). Furthermore, cellobiose PTs in Gram-negative bacteria such as *Klebsiella pneumoniae* have been related to sugar transport [99].

Since Pap is a newly discovered polysaccharide, the specific biosynthetic mechanism and composition have yet to be elucidated. However, the functional and structural comparison of Pap proteins with proteins involved in the synthesis of other polysaccharides has allowed us to describe a predictive model for the Pap biosynthetic complex. Altogether, the predicted functions of each of the Pap components show that Pap is potentially an acidic heteropolysaccharide composed of at least glucuronic or mannuronic acid modified-residues, glucose/GlcNAc, galactose/GalNAc or UDP-L-iduronate, and α-D-galactosyl-di-phosphoundecaprenol. Pap putative composition resembles that of alginate, also an acidic polysaccharide containing mannuronic and glucuronic residues [100], although both biosynthetic pathways contain distinctive predicted enzymes. For instance, the alginate biosynthetic pathway includes a c-di-GMP-binding protein whereas no typical c-di-GMP metabolism nor binding domains have been found in the Pap biosynthetic pathway. On the other hand, the Pap biosynthetic pathway includes predicted deacetylases that are not found in the alginate biosynthetic pathway, suggesting a different composition for both polysaccharides. Besides, both polysaccharides are likely synthesized following different polymerization mechanisms. The biosynthetic pathway for this novel polysaccharide resembles the Wzy-dependent polymerization system for the synthesis of the capsule in *E. coli* [88] and the Psl biosynthetic pathway in *P. aeruginosa* PAO1 [24], relying on a lipid carrier for transportation of the repeating units across the inner membrane and polymerization in the periplasm. In *Pseudomonas*, the lipid carrier-dependent mechanism has been observed for the synthesis of Psl. On the contrary, other well-characterized polysaccharides such as cellulose, alginate, or Pel rely on a direct polymerization across the inner membrane mediated by GTs [24,101,102,103,104]. Furthermore, a certain functional overlap between Pap, alginate, and the lipopolysaccharide (LPS) biosynthetic enzymes is possible as it has been previously suggested for Pel, Psl, alginate, and LPS in *P. aeruginosa* [105,106]. Further research is required to study the putative link between those biosynthetic pathways and whether they share sugar precursors.

**Figure 3:**
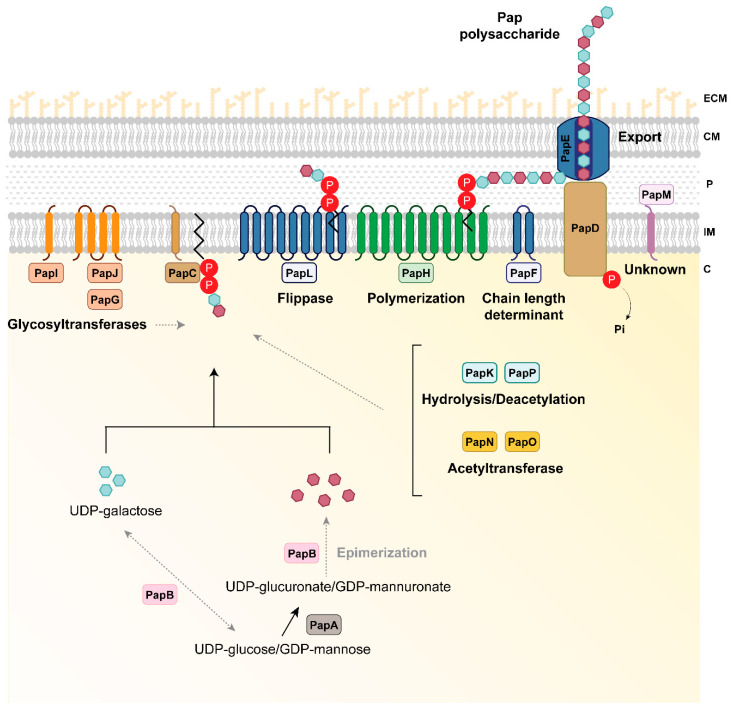
Hypothetical model of *P. fluorescens* F113 Pap biosynthesis. PapA is a UDP-glucose/GDP-mannose dehydrogenase involved in the generation of the Pap polysaccharide residues (purple hexagons). PapB is predicted to be an epimerase that could be involved in the modification of the residues formed by PapA or providing the pool of sugar precursors for the synthesis of Pap. PapC is a phosphotransferase putatively involved in the phosphorylation of UDP-galactose in the synthesis of polysaccharide residues (blue hexagons) and the link of the sugar chain to a lipid carrier in the inner membrane. Once the constituting residues are synthesized, Pap is subjected to several modifications via glycosyltransferases (the cytoplasmic PapG and the inner membrane proteins PapI and PapJ), acetyltransferases (the cytoplasmic PapN and PapO), as well as deacetylation by the cytoplasmic PapK and PapP proteins to ultimately form the mature polysaccharide. The polymerization process and flippase machinery could be carried out by the inner membrane proteins PapH and PapL, respectively. The length of the synthesized polysaccharide could be controlled by the PapF inner membrane protein. The polysaccharide is exported to the milieu via the inner membrane tyrosine-kinase PapD and the outer membrane protein PapE. Whether the polysaccharide is secreted or remains attached to the cell-surface is unknown. There are additional proteins with unknown and no-predicted functions in the biosynthetic process such as PapM. Proteins are colored according to their predicted function as shown in Figure 1. Black solid lines indicate the steps catalyzed by each enzyme. Grey dashed lines indicate putative links or steps that need to be further investigated. C, cytoplasm; ECM, extracellular matrix; IM, inner membrane; P, periplasmic space; OM, outer membrane; P inside a red circle represents phosphate.

The hypothetical model for Pap synthesis is shown in Figure 3. According to the functional predictions of the Pap components, this polysaccharide is composed of at least two different types of residues via the sugar transferases PapA and PapB and the PT PapC. PapC is the protein responsible for linking the repeating units to a lipid carrier in the inner membrane. The Pap biosynthetic pathway involves many enzymes in which three GTs (PapG, PapI, and PapJ), two deacetylases (PapK and PapP), and two acetyltransferases (PapON) were identified with a likely role in the modification of the synthesized polysaccharide. The polymerization/secretion system is putatively composed of a putative flippase system (PapL), which could translocate the polysaccharide to the periplasm, the transmembrane polymerase PapH that could be involved in the production of a long polysaccharide, the chain length determinant PapF, as well as a complex formed by an inner membrane tyrosine-kinase protein PapD and the outer membrane protein PapE that could be acting as a transporter, leading the polysaccharide to the extracellular space. Within the Pap system, there are additional proteins with unclear functions such as PapM. Whether Pap is exported across the outer membrane where it remains attached as a capsule, released as an exopolysaccharide, or even subjected to a combination of both processes, remains unknown, and further analyses are required to elucidate its fate outside the cell.

### 3.3. Phylogenetic Distribution of ECM Components Biosynthetic Pathways in Pseudomonas

In order to determine the distribution of the ECM components in pseudomonads, 611 available complete *Pseudomonas* genomes were analyzed (Supplementary Table S2). For this study, aside from the proteins encoded in F113 (alginate, levan, Pap, PNAG, MapA, LapA, PsmE, Fap, and the Flp/Tad pilus), we also selected proteins related to ECM composition previously described in other pseudomonads. These are the EPSs Pea (PP_3132-3142) and Peb (PP_1788-1795) described in *P. putida*; Pel (PelA-G, PA_3058-3064) and Psl (PslA-O, PA2231-2245) from *P. aeruginosa;* cellulose (WssA-J, PFLU0300-0308) from *P. fluorescens;* the CdrA (PA4625) and LapF (PP_0798-0806) adhesins described in *P. aeruginosa* and *P. putida*, respectively; and the Flp/Tad pilus (PA4295-4306) from *P. aeruginosa*.

A first attempt to study the distribution of biofilm-related traits in pseudomonads was performed by Lind in 2018 [107], in which genes encoding proteins involved in the synthesis of EPSs (alginate, cellulose, PNAG, Pea, Peb, Pel, and Psl) and genes encoding the adhesin LapA and certain regulators, were studied in seven *Pseudomonas* species (*P. fluorescens* SBW25, *P. protegens* Pf-5, *P. putida* KT2440, *P. syringae* pv. *tomato* DC3000, *P. savastanoi* pv. *phaseolicola* 1448A, *P. aeruginosa* PAO1, and *P. stutzeri* ATCC 1758) [107]. However, this study has not delved into the phylogenetic distribution of these components. More recently, Vesga et al. analyzed the distribution of Psl, Pel, and PNAG exopolysaccharides biosynthetic pathways in 97 *Pseudomonas* proteomes [108]. Here, we have studied the phylogenetic distribution of ECM components in roughly 600 *Pseudomonas* genomes.

The identification of orthologous groups (OGs) of proteins among all the *Pseudomonas* genomes analyzed in this study revealed a core-genome of 149 single-copy OGs present in all the genomes that were further processed to construct an ML phylogenetic tree (Supplementary Figure S2). The results show the presence of 20 main groups/SGs, with a distribution similar to the already established phylogeny of *Pseudomonas* based on MLSA and whole-genome analysis [3,39].

**Figure 4:**
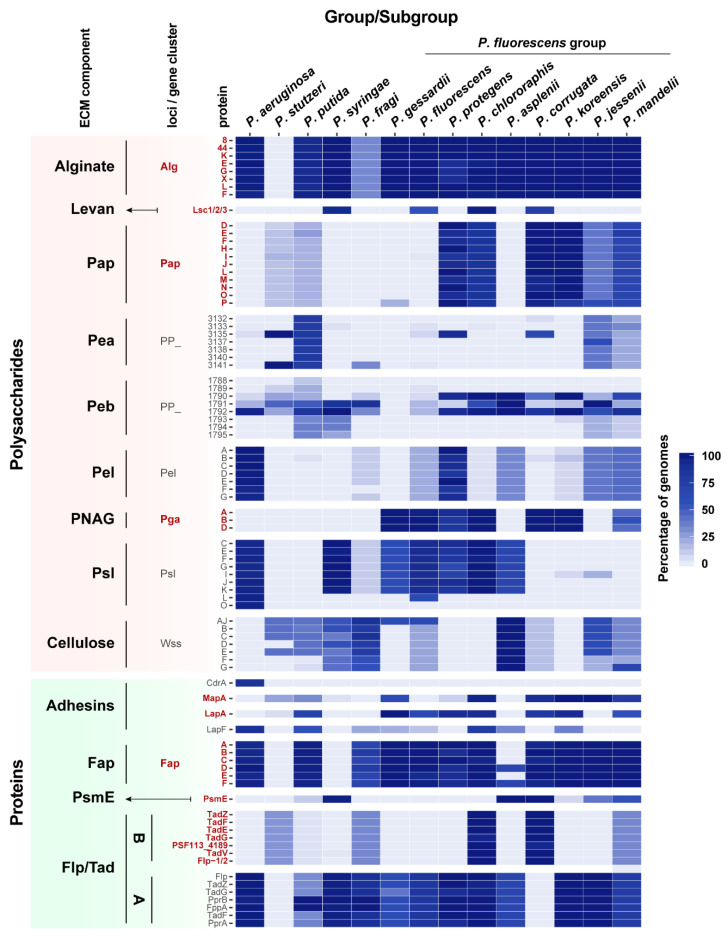
Occurrence of ECM components in *Pseudomonas* strains. Heatmap showing the phylogenetic distribution of ECM components within the *Pseudomonas* genus. Distribution based on the reconstructed phylogenetic tree (Supplementary Figure S2, including the 571 genomes classified in larger groups) and reordered according to Garrido-Sanz et al., 2016 [3] and Garrido-Sanz et al., 2017 [39]. Selected ECM components include alginate, levan, Pap, Pea, Peb, Pel, PNAG, Psl, cellulose, CdrA, MapA, LapA, and LapF, Fap, PsmE, and Flp/Tad pilus type A and B. For the polysaccharides components (except for levan) and the Flp/Tad system, only a subset of proteins is shown because they contain proteins that are part of larger orthologous groups not specific to these clusters. Color scale represents the percentage of genomes containing an ortholog in each group or subgroup. Gene names in bold indicate whether they are present in the F113 genome.

As shown in Figure 4, the distribution analysis (including the 571 out of the 611 genomes that belong to larger phylogenetic groups) has revealed that each of the genomes studied harbor genes encoding diverse polysaccharide biosynthetic pathways or extracellular proteins, indicating that the distribution of ECM components-encoding genes is highly variable within the *Pseudomonas* genus. The specific percentages of genomes for each group/subgroup containing orthologs for ECM components are shown in Supplementary Table S5. The distribution also shows that different combinations of polysaccharides and extracellular proteins could be used by different strains to structure their ECM, and therefore, their biofilms. This evidence highlights the complexity of the biofilm formation process, in which bacteria can use a wide range of polysaccharides and/or proteins to increase adherence, as has been shown in certain strains such as *P. fluorescens* SBW25 [27,109,110]. For instance, in this bacterium, the preferred pathway for pellicle formation is mediated by the synthesis of cellulose. However, in order to increase its adaptative strategies, *P. fluorescens* SBW25 can use PNAG synthesis pathway to achieve adhesion and biofilm formation [27].

Our analysis also shows the existence of a phylogenetic distribution for some of the ECM components within the *Pseudomonas* genus, and an uneven phylogenetic distribution pattern for several polysaccharides and proteins (Levan, Pap, Pea, Peb, Pel, PNAG, Psl, cellulose, adhesins, PsmE, and the Flp/Tad pilus, Figure 4).

Alginate is distributed in almost all *Pseudomonas* phylogenetic groups (Figure 4), except for *P. stutzeri*, in which the complete alginate biosynthetic cluster is absent. Conversely, levan distribution is restricted to *P. syringae* and *P. chlororaphis* groups, and *P. corrugata* and *P. fluorescens* SGs from the Pfl group. No other genome encodes levansucrases, indicating that the presence of levan is restricted to these groups.

Regarding the novel polysaccharide Pap, OG searches revealed the presence of Pap orthologs in the genomes of 112 pseudomonads. It is found mostly within the Pfl group and in a limited number of *P. putida* and *P. stutzeri* genomes. However, it is absent in other larger groups such as *P. aeruginosa* or *P. syringae*. Within the Pfl group, biosynthetic genes for Pap are present in *P. mandelii*, *P. jessenii*, *P. koreensis*, *P. corrugata*, *P. chlororaphis*, and *P. protegens*.

The presence of PNAG is restricted to most Pfl SGs, being present in a high percentage of the genomes included in the *P. koreensis*, *P. mandelii*, *P. corrugata*, *P. fluorescens*, *P. gessardii*, *P. chlororaphis*, and *P. protegens* SGs, and absent in *P. jessenii* and *P. asplenii* SGs. Although its presence in the *Pseudomonas* genus is limited, this operon is found in several distantly related pathogenic bacteria such as in certain *E. coli* strains and human pathogens, including *Staphylococcus epidermidis*, *Klebsiella pneumoniae*, *Yersinia pestis*, and *Acinetobacter baumannii,* where a role in host–bacteria interaction has been reported [111,112].

The Pea EPS is found in a high percentage of *P. putida* group genomes and to a less extent in two SGs from the Pfl group: *P. jessenii* and *P. mandelii*. A similar distribution pattern can be observed for the Peb EPS, which is only found in *P. putida* and partially present in other groups. On the other hand, the Pel EPS does not follow a phylogenetic distribution, being clearly limited to *P. aeruginosa* group and *P. protegens* SG from the Pfl group, and to a less extent to *P. jessenii*, *P. koreensis*, *P. mandelii*, *P. fluorescens*, *P. asplenii*, and *P. fragi* Pfl SGs.

Psl EPS is mainly found in *P. aeruginosa* and *P. syringae* groups, and within the Pfl group in the *P. fluorescens*, *P. gessardii*, *P. chlororaphis*, *P. protegens*, *P. asplenii*, and certain genomes of *P. fragi* SGs. Interestingly, Psl is not present in the groups where Pap is extensively found, except for *P. chlororaphis* and *P. protegens* in which a high percentage of orthologs for both EPSs are found.

Cellulose is present mostly in *P. putida* and *P. syringae* groups and in *P. asplenii* SG, and certain *P. fragi*, *P. jessenii*, *P. mandelii*, *P. corrugata*, and *P. fluorescens* Pfl SGs. In general, bacteria that produce cellulose do not produce PNAG, which might suggest that both EPSs play similar roles in the structure of ECMs and biofilms.

Regarding the extracellular proteins, the adhesin CdrA has been only found in *P. aeruginosa* genomes, this adhesin being specific to this group. However, other adhesins are present in the rest of the groups, and some of them have two or more adhesins simultaneously (Figure 4). MapA is found mostly in *P. jessenii*, *P. koreensis*, *P. mandelii*, *P. corrugata*, *P. chlororaphis*, and *P. gessardii* Pfl SGs, and in some *P. putida* and *P. stutzeri* groups. On the other hand, LapA is widely distributed within the Pfl group and *P. putida*, while LapF is found in some of the Pfl SGs, *P. putida*, and *P. aeruginosa*. The putative adhesin/extracellular epimerase PsmE is only present in a limited set of groups: *P. syringae* group and *P. asplenii, P. corrugata*, and certain *P. mandelii*, and *P. jessenii* Pfl SGs, and in a lower percentage of genomes belonging to the *P. putida* group. Although one of the putative functions of PsmE is an alginate epimerase and acetylhydrolase, this protein is not present in all the alginate-containing groups.

Fap is also found widely distributed within the genus. Interestingly, no orthologous sequences of amyloid proteins were found in the *P. syringae* group although it is present in its closest relatives, including *P. fragi* and the Pfl group. The *fap* cluster is not limited to pseudomonads, as it has been found widespread in proteobacteria and it has been determined that 36% of the *fap* carrying bacteria have a rhizosphere lifestyle [38].

The complete type IVb Flp/Tad pilus cluster found in the F113 genome has a restricted distribution within the *Pseudomonas* genus. Nearly 100% of genomes within the *P. corrugata* and the *P. chlororaphis* Pfl SGs harbor this gene cluster, while in the *P. mandelii* and *P. fragi* Pfl SGs, and the *P. stutzeri* group its presence is more limited. It is important to note that *flp-1* and *flp-2*, which are described in the F113 genome, belong to the same OG. However, *flp* from *P. aeruginosa* belongs to a different OG, suggesting a different nature for this Flp/Tad pilus in both groups. As observed in this study, The Flp/Tad pilus found in *P. aeruginosa*, hereafter named Flp/Tad type A, is more broadly distributed in the genus than the one found in F113, from now on Flp/Tad type B.

It is noteworthy that Pap, PNAG, MapA, and Flp/Tad type B components were found more restricted to certain SGs within the Pfl group and they are not present in other large groups such as *P. aeruginosa*, *P. putida,* or *P. syringae* (Figure 4). The former Pfl SGs are adapted to the plant environment. Although there is evidence of plant-pathogens such as *P. corrugata* and *P. mediterranea* in the *P. corrugata* SG [113], the remaining SGs have mainly a commensal lifestyle and are largely known for their PGPR traits [3,114]. It is therefore likely that the production of these components is relevant and specific for beneficial plant–bacteria interactions and adaptation to the rhizosphere environment.

### 3.4. Phylogeny of Flp/Tad and Pap Pseudomonas

**Figure 5:**
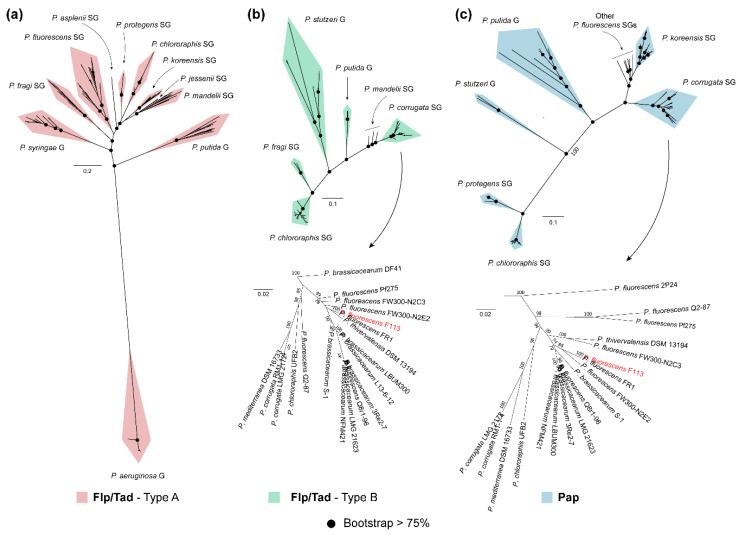
Phylogenetic analysis of Flp/Tad and Pap components in the genus *Pseudomonas*. Unrooted ML trees of Flp/Tad type A (**a**), Flp/Tad type B (**b**) and Pap (**c**) components within pseudomonads built using the concatenated alignment of TadZG, PprB, FppA, TadF, and PprA (**a**); TadB, PSF113-4189, TadEGZ, and CpaE-like (**b**); and PapDEFHIJLMO (**c**), respectively. Black dots in nodes indicate bootstrap values equal to or greater than 75%. Taxa belonging to *Pseudomonas corrugata* subgroup from the *P. fluorescens* group, which includes *P. fluorescens* F113 (red typing), is shown in greater detail at the bottom of Flp/Tad type B (**b**) and Pap (**c**) matrix components. The complete list of taxa used in this analysis can be found in Supplementary Table S6.

In this work, we have identified a predicted novel polysaccharide, Pap, and a new type of the Flp/Tad pilus in pseudomonads, distinct from the one already described in *P. aeruginosa*, that we have named Flp/Tad type B. Thus, we decided to analyze the phylogenetic relationships among *Pseudomonas* containing the Flp/Tad type A and B, and Pap components. In this study, 105, 82, and 456 taxa were included in the Pap, Flp/tad type B, and A phylogenetic trees, respectively (Figure 5).

As shown in Figure 5a, the phylogenetic tree for the Flp/Tad type A pilus includes 10 different clades. The *P. aeruginosa* clade is found very distant from the rest of the clades, which correspond to *P. putida* and *P. syringae* groups and most of the SGs from the Pfl group of species: *P. fragi*, *P. fluorescens*, *P. asplenii*, *P. protegens*, *P. chlororaphis*, *P. koreensis*, *P. jessenii,* and *P. mandelii*. On the other hand, the Flp/Tad type B phylogenetic tree (Figure 5b) is composed of six clades: a first clade constituted by *P. corrugata* and *P. mandelii* SGs from the Pfl group, *P. putida* and *P. stutzeri* groups, and more distantly *P. fragi* and *P. chlororaphis* SGs from the Pfl group. Additionally, in Figure 5c, the Pap-based phylogenetic tree is shown, including seven clades with a similar pattern to the one observed for Flp/Tad type B. The first clade includes the strains belonging to the *P. corrugata* SG. The second clade comprises strains belonging to the *P. koreensis* SG, several taxa belonging to different Pfl group SGs, *P. putida* group, *P. stutzeri* group, and more distantly *P. protegens* and *P. chlororaphis* SGs from the Pfl group.

All the clades were already clearly distinguished in the phylogenetic tree inferred from the concatenation of single-copy orthologous sequences (Supplementary Figure S2). However, the clades shown in the phylogenetic trees of Flp/Tad type B and Pap, represented in Figure 5b,c respectively, differ in the branching pattern; evidenced by *P. corrugata* SG from the Pfl group being phylogenetically closer to *P. putida* or *P. stutzeri* groups and more distant to *P. chlororaphis* SG from the Pfl group, supported by high bootstrap scores. Thereby, revealing that the evolution of Flp/Tad type B and Pap is not in accordance with the evolution of the genomes in which they appeared, and could suggest a specific ingroup divergence of these EMC components. Furthermore, the similarities between the phylogenetic trees of Flp/Tad type B (Figure 5b) and Pap, (Figure 5c) which can be especially observed in the *P. corrugata* SG from the Pfl group, suggest a possible co-evolution of both components in the genomes harboring them.

In *Pseudomonas*, most of the strains contain only one of the Flp/Tad configurations (432 of the analyzed strains) and the occurrence of type A or B is likely linked with its bacteria–host lifestyle. The plant–bacteria relationship of the taxa included in the phylogenetic analysis (excluding the *P. aeruginosa* group as members of this group contain neither Flp/Tad type B nor Pap) was assessed based on the reported lifestyle information in the literature and shown in Supplementary Table S6. Interestingly, several of the genomes containing orthologs for Pap and Flp/Tad type B proteins have a beneficial relationship with plants according to literature (Supplementary Table S6). The only known pathogens that contain these components are *P. corrugata* LMG2172 and *P. mediterranea* DSM 16733, both recognized phytopathogenic species [115,116]. On the contrary, genomes containing Flp/Tad type A orthologs have been equally reported either as PGPRs or phytopathogens (Supplementary Table S6). Therefore, these findings suggest a role for Pap and Flp/Tad type B in rhizosphere colonization and beneficial plant–bacteria interactions. The production of exopolysaccharides is one of the major traits associated with robust colonization of the rhizosphere lifestyle in members of the genera *Agrobacterium*, *Asticcacaulis*, *Ensifer*, *Lysobacter*, *Pedobacter*, and *Streptomyces* [117], as it allows the attachment to root surfaces [118]. Similarly, the pivotal role of the Flp/Tad pilus in colonization and virulence has been extensively studied in opportunistic pathogens of humans such as *Aggregatibacter* spp. [119,120], *Vibrio* spp. [121,122], *Haemophilus* spp. [123] or *P. aeruginosa* [83], and plant pathogens such as *Pectobacterium* spp., [124] or *Ralstonia solanacearum* [125]. However, little is known about its relevance in specific non-pathogenic or beneficial bacteria–host interactions.

The co-occurrence of Flp/Tad type A and B was only found in the PGPR *P. chlororaphis* SG (47/52 strains), most of them also containing Pap (40/52 strains contain the three components). Aside from *P. chlororaphis*, the co-occurrence of Flp/Tad pilus type A and B is found in the following specific strains: *P. psychrophila* LMG24276, *P. lini* DSM16768, *P. fragi* strains NMC25 and NRRL B-727, and *P. frederiksbergensis* strains AS1 and KNU-1 (Supplementary Table S6). The presence of multiple *tad* loci was previously reported in the pathogen *Vibrio vulnificus* mediating its invasion [121], and also in *Bordetella pertussis*, *Burkholderia pseudomallei*, *Mesorhizobium loti*, or *Sinorhizobium meliloti* [36].

As mentioned before, species belonging to the *P. corrugata* SG from the Pfl group, such as *P. corrugata* or *P. mediterranea,* have been demonstrated to inhabit soils and plants, generally symptomless. However, they can also have deleterious effects in plants under certain conditions [115,126,127], causing for instance tomato pith necrosis [128] or they can be used for the biological control of some phytopathogens [126,127]. Thus, the presence of the Flp/Tad type B and Pap components in the *P. corrugata* and *P. mediterranea* reported pathogenic strains could be related to their competitive rhizosphere colonization ability. Conversely, a large set of *P. brassicacearum* and related species with known beneficial effects in plants [113] were found in this study to contain in their genomes the clusters necessary for the synthesis of these ECM components.

### 3.5. Synteny Analysis of the Two Pseudomonads Flp/Tad Pilus

**Figure 6:**
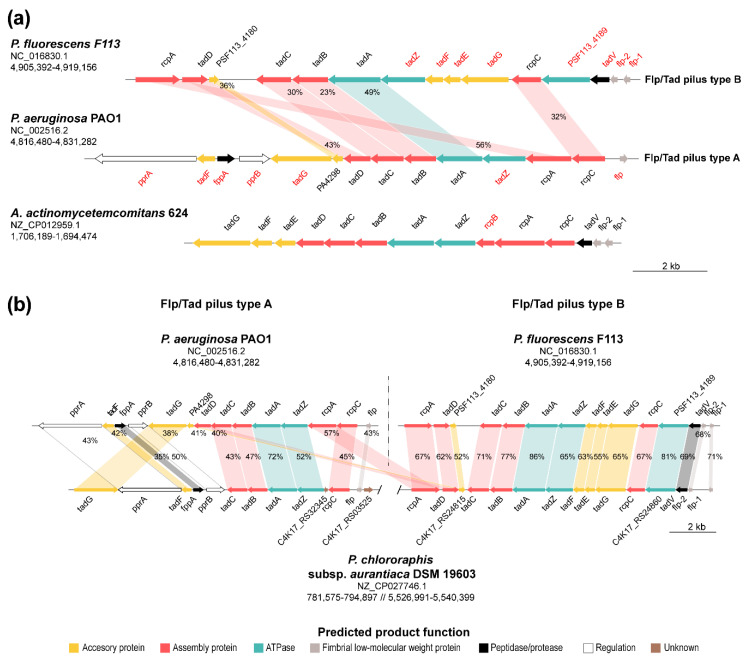
Synteny and sequence identity of *flp/tad* type A and B gene clusters. (**a**) Gene organization of *P. fluorescens* F113 (type B), *P. aeruginosa* PAO1 (type A), and *Aggregatibacter actinomycetemcomitans* 624 *flp/tad* gene clusters. Genes typed in red are specific of types A or B Flp/Tad pilus encoding clusters and therefore are not orthologous. (**b**) Synteny of the *flp/tad* clusters type A (*P. aeruginosa* PAO1) and B (*P. fluorescens* F113) with *P. chlororaphis* subsp. *aurantiaca* DSM 19603, harboring both clusters encoded in its genome. Arrows indicate the relative location, size, and direction of transcription of ORFs. Arrow colors depict the predicted gene product function as shown in the legend. Colored areas indicate genes belonging to the same OG and percentages depict amino acid sequence identity. NCBI access numbers and genomic coordinates of the regions shown are specified under the species names. Genes are scaled.

As described earlier, we have reported the presence of Flp/Tad type IVb pilus in almost all the complete *Pseudomonas* sequences thus far. Furthermore, we identified two different types of the *flp/tad* pilus biosynthetic cluster in this genus. The one previously described in *P. aeruginosa* and more broadly distributed, named *flp/tad* type A, and the one we have described here and that we have named *flp/tad* type B, with a more limited distribution. In order to further study the two distinct types of the Flp/Tad pilus distributed among the *Pseudomonas* genus, we have carried out a syntenic comparison of the F113 and *P. aeruginosa* PAO1 *flp/tad* clusters (Figure 6a), and between F113, *P. aeruginosa* PAO1 and a representative of *P. chlororaphis* (*P. chlororaphis* subsp. *aurantiaca* DSM 19603) in which both types of pilus were simultaneously found (Figure 6b). As shown in Figure 6a, the syntenic organization of *flp/tad* type A and B from *P. aeruginosa* PAO1 and F113, respectively, is poorly conserved, sharing a set of seven genes (*rcpAC, tadABCD,* PSF113_4180/PA4298). Another difference between both types of *flp/tad* clusters is the existence of a duplication event in the *flp/tad* type B cluster of the *flp* gene, encoding the pilin, as shown in other bacteria such as *Aggregatibacter actinomycetemcomitans* [37,119,129]. Moreover, a variable number of *flp* genes has been previously observed across different species such as *Bdellovibrio bacteriovoru* with four *flp* genes [130]. The *flp/tad* type B cluster shows a higher level of synteny with other bacteria, such as species of *Aggregatibacter* and is conserved in other species of the Pfl group and *P. stutzeri*. Both configurations have several putative transcriptional units operating in different directions [83,131]. On the contrary, there is low sequence identity between the peptidases (FppA and TadV), ATPases (TadZ and PSF113_4189), regulatory (PprA, PprB), and accessory (TadE, TadF, and TadH) proteins of each pilus type and they belong to different OGs in F113 and *P. aeruginosa* PAO1.

Unlike the *flp/tad* type A, the *flp/tad* type B (Figure 6b) shows a highly conserved syntenic organization between F113 and *P. chlororaphis* subsp. *aurantiaca* DSM 19603, which encodes both Flp/Tad pilus types and is more distant in the phylogenetic study (Figure 5b). Nonetheless, the syntenic organization of the *flp/tad* type A gene cluster in this same bacterium is not as conserved compared to *P. aeruginosa* PAO1, and some of the genes are missing (*rcpA*, *tadC*, and PA4298). However, homologous genes to those missing in type A cluster are found in the type B cluster, suggesting that they might be interchangeable between both pilus types. As shown in Figure 6b, there is a low amino acid sequence identity between *P. aeruginosa* PAO1 and *P. chlororaphis* subsp. *aurantiaca* DSM 19603. Thus, an event of horizontal gene transfer is very unlikely, at least recently, as previously suggested for the *flp/tad* cluster. The *tad* locus has been previously characterized as a mobile genomic island and named “widespread colonization island” due to its requirement for the colonization of a variety of environmental niches and bacteria [36,132]. Indeed, this locus has experienced duplication, loss, recombination, and horizontal gene transfer between distant bacterial relatives, and more recently between closer relatives [36].

## 4. Conclusions

In conclusion, these in silico analyses allowed the identification of nine genes/gene clusters putatively involved in the synthesis of extracellular matrix components in *Pseudomonas fluorescens* F113: the polysaccharides PNAG, alginate, levan; a novel *Pseudomonas* acidic polysaccharide (Pap); and the proteins or proteinaceous structures Fap, LapA, MapA, PsmE, and Flp/Tad pilus. Aside from the novel Pap polysaccharide that we have described here, we identified the presence of two different Flp/Tad type IVb pilus distributed among pseudomonads, namely Flp/Tad pilus type A and B.

This study has revealed an even phylogenetic distribution for alginate and an uneven phylogenetic distribution for most of the ECM components, the polysaccharides levan, Pap, Pea, Peb, Pel, PNAG, Psl, and cellulose; and the adhesins, PsmE and the Flp/Tad pili. Furthermore, the phylogenetic relationship of Flp/Tad type B and Pap novel matrix components suggests their co-evolution. Besides, the biosynthetic machinery necessary for the synthesis of Pap and Flp/Tad type B components is found in plant-associated bacteria and mainly with a commensal or beneficial lifestyle, suggesting its likely role during the rhizosphere colonization process.

## Figures and Tables

**Table 1:** Predicted localization, domains, and function for Pap proteins in *Pseudomonas fluorescens* F113.

Protein (PSF113_)	Length (Amino Acids)	Cellular Localization ^1^	Transmembrane Domains ^1^	Domain No. ^1^	Predicted Function
**PapA (PSF113_1970)**	454	Cytoplasmic	0	3	UDP-glucose 6-dehydrogenase
**PapB (PSF113_1969)**	352	Cytoplasmic	0	5	NAD-dependent epimerase/dehydratase
**PapC (PSF113_1968)**	248	Inner membrane	1	1	Undecaprenyl-phosphate galactose phosphotransferase/Glycosyltransferase
**PapD (PSF113_1967)**	214	Inner membrane	0	1	Protein EpsD/CpsD/CapB family tyrosine-kinase protein
**PapE (PSF113_1966)**	340	Outer membrane	0	3	Putative polysaccharide biosynthesis/export family protein
**PapF (PSF113_1965)**	530	Inner membrane	2	1	Lipopolysaccharide biosynthesis protein/Chain length determinant
**PapG (PSF113_1964)**	367	Cytoplasmic	0	5	Group 4 glycosyl transferase
**PapH (PSF113_1963)**	456	Inner membrane	11	1	Hypothetical protein/Polymerase/O-Antigen ligase family protein
**PapI (PSF113_1962)**	319	Inner membrane	1	4	Group 2 glycosyl transferase family protein
**PapJ (PSF113_1961)**	378	Inner membrane	4	5	Group 2 glycosyl transferase family protein
**PapK (PSF113_1960)**	334	Cytoplasmic	0	1	Polysaccharide deacetylase
**PapL (PSF113_1959)**	434	Inner membrane	9	3	Polysaccharide biosynthesis membrane protein/Oligosaccharide flippase family protein
**PapM (PSF113_1958)**	658	Periplasmic	1	-	Hypothetical protein
**PapN (PSF113_1957)**	229	Cytoplasmic	0	-	Hypothetical protein
**PapO (PSF113_1956)**	391	Cytoplasmic	0	1	Cellulose biosynthesis protein/GNAT family N-acetyltransferase
**PapP (PSF113_1955)**	260	Cytoplasmic	0	1	Cellobiose phosphotransferase system YdjC-like protein

^1^ Additional information in Supplementary Table S4.

## Supplementary Materials

The following are available online at https://www.mdpi.com/2076-2607/8/11/1740/s1, Figure S1: Domain analysis of *Pseudomonas fluorescens* F113 extracellular proteins MapA and PsmE; Figure S2: Maximum-likelihood phylogenetic tree of the 611 *Pseudomonas* based on 149 single-copy amino acid sequences; Table S1: Comparison of *Pseudomonas fluorescens* F113 ECM components outside *Pseudomonadales*; Table S2: List of *Pseudomonas* genomes used in this study; Table S3: Nucleotide and amino acid sequences comparison of *Pseudomonas fluorescens* F113 gene clusters inside the genus *Pseudomonas*; Table S4: Predicted localization, domains, and function of *Pseudomonas* acidic polysaccharide (Pap) biosynthetic proteins in *Pseudomonas fluorescens* F113; Table S5: Percentage of *Pseudomonas* genomes containing an ortholog of extracellular matrix components; Table S6: Pseudomonads containing the fimbrial low-molecular-weight protein (*flp*)/tight adherence (*tad*) and/or *Pseudomonas* acidic polysaccharide (*pap*) biosynthetic clusters.
