# Supplementary material for: In Silico Characterization and Phylogenetic Distribution of Extracellular Matrix Components in the Model Rhizobacteria Pseudomonas fluorescens F113 and Other Pseudomonads
Source: Microorganisms. 2020 Nov 6;8(11):1740. doi: 10.3390/microorganisms8111740 (PMC7716237; doi:10.3390/microorganisms8111740)
Supplement: Supplementary file 1 [file microorganisms-08-01740-s001.zip › Supplementary_Figure_2.pdf]

Supplementary Figure 2. Maximum-likelihood phylogenetic tree of the 611 *Pseudomonas* based on 149 single-copy amino acid sequences: the number of genomes included in each group or subgroup is indicated with a color scale and scaled circles. Names in bold indicate verified groups according to the current *Pseudomonas* classification [1]) Maximum likelihood trees were inferred with RaxML. The phylogenetic tree was constructed using MEGA X and drawn to scale. Bootstrap values are shown next to the corresponding nodes.

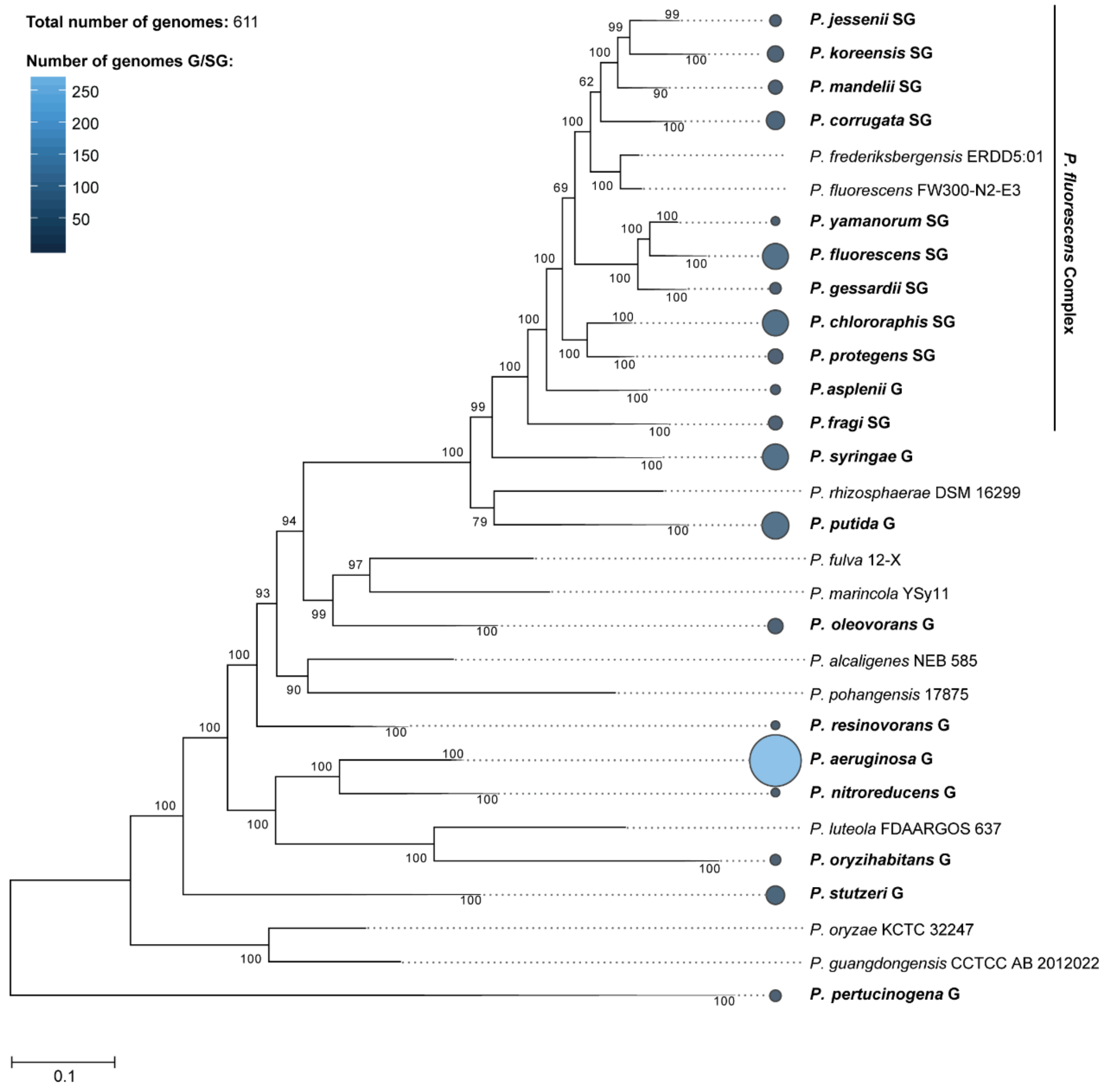

References

1. Garrido-Sanz, D.; Meier-Kolthoff, J.P.; Göker, M.; Martín, M.; Rivilla, R.; Redondo-Nieto, M. Genomic and genetic diversity within the *Pseudomonas fluorescens* complex. *PLoS One* **2016**, *11*, e0150183, doi:10.1371/journal.pone.0150183.
