# Supplementary material for: In Silico Characterization and Phylogenetic Distribution of Extracellular Matrix Components in the Model Rhizobacteria Pseudomonas fluorescens F113 and Other Pseudomonads
Source: Microorganisms. 2020 Nov 6;8(11):1740. doi: 10.3390/microorganisms8111740 (PMC7716237; doi:10.3390/microorganisms8111740)
Supplement: Supplementary file 1 [file microorganisms-08-01740-s001.zip › Supplementary_Table_1.pdf]

Supplementary Table 1. Comparison of *Pseudomonas fluorescens* F113 ECM components outside *Pseudomonadales*

Name	Locus (PSF113_)	Protein predicted function	Protein homology	
pgaA	161	Poly-beta-1,6 N-acetyl-D-glucosamine export porin	*Paraburkholderia insulsa*	70%
			Achromobacter spp.	
pgaB	162	Poly-beta-1,6-N-acetyl-D-glucosamine N-deacetylase	*Paraburkholderia insulsa*	63-77 %
			Achromobacter spp.	
			Cupriavidus gilardii	
pgaC	163	Poly-beta-1,6 N-acetyl-D-glucosamine synthase, catalytic subunit	*Paraburkholderia insulsa*	72-85 %
			Achromobacter spp.	
pgaD	164	Poly-beta-1,6-N-acetyl-D-glucosamine synthase, regulatory subunit; c-di-GMP-stimulated activity and dimerization	*Paraburkholderia insulsa*	36-46 %
			Xenorhabdus beddingii	
			Citrobacter werkmanii	
papA	1970	UDP-glucose/GDP-mannose dehydrogenase family protein	*Halomonas* sp. KM-1	61-62 %
			Serratia sp. M24T3	
			Alishewanella aestuarii	
			Chelativorans sp. BNC1	
papB	1969	Nucleoside-diphosphate-sugar epimerase	*Geobacillus* sp. WCH70	59-62 %
		NAD dependent epimerase/dehydratase	*Thermithiobacillus tepidarius*	
			Balneatrix alpaca	
			Bacillus oceanisediminis	
papC	1968	Undecaprenyl-phosphate galactose	*Zooshikella ganghwensis*	57-60 %
			Desulfobulbus japonicas	
			Sphingomonas sp. TZW2008	
papD	1967	Autophosphorylating protein tyrosine kinase	*Paraburkholderia insulsa*	71-72 %
			Achromobacter spp.	
			Cupriavidus gilardii	
papE	1966	Polysaccharide biosynthesis/export protein	*Zooshikella ganghwensis*	32-38 %
			Gamma proteobacterium HdN1	
			Desulfobulbus japonicus	
papF	1965	Uncharacterized protein involved in exopolysaccharide biosynthesis	*Zooshikella ganghwensis*	29-40 %
			Gamma proteobacterium HdN1	
			Desulfobulbus japonicus	
papG	1964	Glycosyl transferases group 1	*Zooshikella ganghwensis*	33-35 %
			Gamma proteobacterium HdN1	
			Geobacter sulfurreducens	
papH	1963	O-Antigen ligase	*Zooshikella ganghwensis*	38-43 %
			Gamma proteobacterium HdN1	

			Desulfobulbus japonicus	
papI	1962	Glycosyl transferases group 1	*Desulfobulbus japonicus*	40-43 %
			Gamma proteobacterium HdN1	
			Zooshikella ganghwensis	
papJ	1961	Glycosyl transferase, group 2 family protein	Gamma proteobacterium HdN1	40-47 %
			Desulfobulbus japonicus	
			Zooshikella ganghwensis	
papK	1960	Polysaccharide deacetylase	*Halomonas xinjiagensis*	38-41 %
			Halomonas anticariensis	
			Halomonas xianhensis	
papL	1959	Polysaccharide biosynthesis protein	Gamma proteobacterium HdN1	37-40 %
			Zooshikella ganghwensis Desulfobulbus japonicus	
papM	1958	Hypothetical protein (no putative conserved domains detected)	*Zooshikella ganghwensis* Gamma proteobacterium HdN1	35-42 %
			Desulfobulbus japonicus	
papN	1957	Hypothetical protein (no putative conserved domains detected)	*Zooshikella ganghwensis*	24-29 %
			Desulfobulbus japonicus	
papO	1956	Acetyltransferase (GNAT) domain	*Microvirga guangxiensis*	26-32 %
			Gamma proteobacterium HdN1	
			Desulfobulbus japonicus	
			Zooshikella ganghwensis	
papP	1955	YdjC-like protein	*Lachnospiraceae*	35-37 %
lapA	208	Protein LapA	*Stenotrophomonas rhizophila*	48-56%
			Pantoea	
mapA	1511	Putative hemagglutinin/hemolysin-like protein	*Pantoea*	40-56%
			Aeromonas	
psmE	3004	Protein PsmE	*Asticcaculis sp*	40-48%
			Tanacetum cinerariifolium	
			Microvirga sp. BSC39	
			Methylobacter	
