# Supplementary material for: In Silico Characterization and Phylogenetic Distribution of Extracellular Matrix Components in the Model Rhizobacteria Pseudomonas fluorescens F113 and Other Pseudomonads
Source: Microorganisms. 2020 Nov 6;8(11):1740. doi: 10.3390/microorganisms8111740 (PMC7716237; doi:10.3390/microorganisms8111740)
Supplement: Supplementary file 1 [file microorganisms-08-01740-s001.zip › Supplementary_Table_2.pdf]

Supplementary Table 2. List of *Pseudomonas* genomes used in this study.

Name	Assembly level	Assembly accesion identifier	Assigned group
***Pseudomonas aeruginosa* PSE305**	Chromosome	GCF_000750905.1	*P. aeruginosa*
***Pseudomonas aeruginosa* CCBH4851**	Chromosome	GCF_000763245.2	*P. aeruginosa*
***Pseudomonas aeruginosa* F22031**	Complete Genome	GCF_000816985.1	*P. aeruginosa*
***Pseudomonas aeruginosa* NCGM1984**	Complete Genome	GCF_000829255.1	*P. aeruginosa*
***Pseudomonas aeruginosa* NCGM1900**	Complete Genome	GCF_000829275.1	*P. aeruginosa*
***Pseudomonas aeruginosa* FRD1**	Complete Genome	GCF_000829885.1	*P. aeruginosa*
***Pseudomonas aeruginosa* Carb01 63**	Complete Genome	GCF_000981825.1	*P. aeruginosa*
***Pseudomonas aeruginosa* S04 90**	Chromosome	GCF_000988505.1	*P. aeruginosa*
***Pseudomonas aeruginosa* F9676**	Complete Genome	GCF_001077475.1	*P. aeruginosa*
***Pseudomonas aeruginosa* PA1RG**	Complete Genome	GCF_001293085.1	*P. aeruginosa*
***Pseudomonas aeruginosa* VA-134**	Complete Genome	GCF_001447845.1	*P. aeruginosa*
***Pseudomonas aeruginosa* NCTC10332**	Complete Genome	GCF_001457615.1	*P. aeruginosa*
***Pseudomonas aeruginosa* 12-4-4 59**	Complete Genome	GCF_001482325.1	*P. aeruginosa*
***Pseudomonas aeruginosa* S86968**	Complete Genome	GCF_001515845.2	*P. aeruginosa*
***Pseudomonas aeruginosa* T38079**	Complete Genome	GCF_001515915.2	*P. aeruginosa*
***Pseudomonas aeruginosa* T52373**	Complete Genome	GCF_001516005.1	*P. aeruginosa*
***Pseudomonas aeruginosa* T63266**	Complete Genome	GCF_001516105.1	*P. aeruginosa*
***Pseudomonas aeruginosa* W16407**	Complete Genome	GCF_001516165.2	*P. aeruginosa*
***Pseudomonas aeruginosa* W36662**	Complete Genome	GCF_001516185.2	*P. aeruginosa*
***Pseudomonas aeruginosa* W45909**	Complete Genome	GCF_001516205.2	*P. aeruginosa*
***Pseudomonas aeruginosa* W60856**	Complete Genome	GCF_001516225.2	*P. aeruginosa*
***Pseudomonas aeruginosa* F23197**	Complete Genome	GCF_001516245.2	*P. aeruginosa*
***Pseudomonas aeruginosa* F30658**	Complete Genome	GCF_001516265.1	*P. aeruginosa*
***Pseudomonas aeruginosa* H5708**	Complete Genome	GCF_001516305.2	*P. aeruginosa*
***Pseudomonas aeruginosa* H27930**	Complete Genome	GCF_001516325.2	*P. aeruginosa*
***Pseudomonas aeruginosa* H47921**	Complete Genome	GCF_001516345.1	*P. aeruginosa*
***Pseudomonas aeruginosa* M1608**	Complete Genome	GCF_001516365.2	*P. aeruginosa*

***Pseudomonas aeruginosa* M37351**	Complete Genome	GCF_001516385.1	*P. aeruginosa*
***Pseudomonas aeruginosa* USDA-ARS-USMARC-41639**	Complete Genome	GCF_001518975.1	*P. aeruginosa*
***Pseudomonas aeruginosa* X78812**	Complete Genome	GCF_001542795.2	*P. aeruginosa*
***Pseudomonas aeruginosa* F9670**	Complete Genome	GCF_001542835.1	*P. aeruginosa*
***Pseudomonas aeruginosa* NCGM257**	Complete Genome	GCF_001547955.1	*P. aeruginosa*
***Pseudomonas aeruginosa* 8380**	Complete Genome	GCF_001548135.1	*P. aeruginosa*
***Pseudomonas aeruginosa* IOMTU 133**	Complete Genome	GCF_001548335.1	*P. aeruginosa*
***Pseudomonas aeruginosa* F63912**	Complete Genome	GCF_001594325.2	*P. aeruginosa*
***Pseudomonas aeruginosa* N17-1**	Complete Genome	GCF_001606045.1	*P. aeruginosa*
***Pseudomonas aeruginosa* BAMCPA07-48**	Complete Genome	GCF_001632245.1	*P. aeruginosa*
***Pseudomonas aeruginosa* PA121617**	Complete Genome	GCF_001679685.1	*P. aeruginosa*
***Pseudomonas aeruginosa* ATCC 27853**	Complete Genome	GCF_001687285.1	*P. aeruginosa*
***Pseudomonas aeruginosa* PA D1**	Complete Genome	GCF_001721745.1	*P. aeruginosa*
***Pseudomonas aeruginosa* PA D2**	Complete Genome	GCF_001721765.1	*P. aeruginosa*
***Pseudomonas aeruginosa* PA D9**	Complete Genome	GCF_001721785.1	*P. aeruginosa*
***Pseudomonas aeruginosa* PA D16**	Complete Genome	GCF_001721805.1	*P. aeruginosa*
***Pseudomonas aeruginosa* PA D22**	Complete Genome	GCF_001721825.1	*P. aeruginosa*
***Pseudomonas aeruginosa* PA D25**	Complete Genome	GCF_001721845.1	*P. aeruginosa*
***Pseudomonas aeruginosa* DN1**	Complete Genome	GCF_001722005.2	*P. aeruginosa*
***Pseudomonas aeruginosa* PA D5**	Complete Genome	GCF_001722025.1	*P. aeruginosa*
***Pseudomonas aeruginosa* PA D21**	Complete Genome	GCF_001722045.1	*P. aeruginosa*
***Pseudomonas aeruginosa* ATCC 15692**	Complete Genome	GCF_001729505.1	*P. aeruginosa*
***Pseudomonas aeruginosa* FA-HZ1**	Complete Genome	GCF_001750705.1	*P. aeruginosa*
***Pseudomonas aeruginosa* PA1088**	Complete Genome	GCF_001792835.1	*P. aeruginosa*
***Pseudomonas aeruginosa* PA8281**	Complete Genome	GCF_001792855.1	*P. aeruginosa*
***Pseudomonas aeruginosa* PA11803**	Complete Genome	GCF_001792875.1	*P. aeruginosa*
***Pseudomonas aeruginosa* PA7790**	Complete Genome	GCF_001870265.1	*P. aeruginosa*
***Pseudomonas aeruginosa* B10W**	Complete Genome	GCF_001874465.1	*P. aeruginosa*

Pseudomonas aeruginosa **Paer4 119**	Complete Genome	GCF_001879525.1	*P. aeruginosa*
Pseudomonas aeruginosa **SCVFeb**	Complete Genome	GCF_001900195.1	*P. aeruginosa*
Pseudomonas aeruginosa **SCVJan**	Complete Genome	GCF_001900225.1	*P. aeruginosa*
Pseudomonas aeruginosa **NHmuc**	Complete Genome	GCF_001900265.1	*P. aeruginosa*
Pseudomonas aeruginosa **PA 154197**	Complete Genome	GCF_002075065.1	*P. aeruginosa*
Pseudomonas aeruginosa **RIVM-EMC2982**	Complete Genome	GCF_002085605.1	*P. aeruginosa*
Pseudomonas aeruginosa **E6130952**	Complete Genome	GCF_002085755.1	*P. aeruginosa*
Pseudomonas aeruginosa **PASGNDM699**	Complete Genome	GCF_002104595.1	*P. aeruginosa*
Pseudomonas aeruginosa **PASGNDM345**	Complete Genome	GCF_002104615.1	*P. aeruginosa*
Pseudomonas aeruginosa **M8A1**	Chromosome	GCF_002116945.1	*P. aeruginosa*
Pseudomonas aeruginosa **M8A4**	Chromosome	GCF_002116985.1	*P. aeruginosa*
Pseudomonas aeruginosa **M28A1**	Chromosome	GCF_002117025.1	*P. aeruginosa*
Pseudomonas aeruginosa **Pb18**	Chromosome	GCF_002117045.1	*P. aeruginosa*
Pseudomonas aeruginosa **Pa124**	Complete Genome	GCF_002192475.1	*P. aeruginosa*
Pseudomonas aeruginosa **Pa58**	Complete Genome	GCF_002192495.1	*P. aeruginosa*
Pseudomonas aeruginosa **Pa84**	Complete Genome	GCF_002205335.1	*P. aeruginosa*
Pseudomonas aeruginosa **Pa127**	Complete Genome	GCF_002205355.1	*P. aeruginosa*
Pseudomonas aeruginosa **Pa1242**	Complete Genome	GCF_002205375.1	*P. aeruginosa*
Pseudomonas aeruginosa **Pa1207**	Complete Genome	GCF_002208645.1	*P. aeruginosa*
Pseudomonas aeruginosa **PA83**	Complete Genome	GCF_002215345.1	*P. aeruginosa*
Pseudomonas aeruginosa **L10**	Complete Genome	GCF_002223805.1	*P. aeruginosa*
Pseudomonas aeruginosa **Ocean-1155**	Complete Genome	GCF_002237405.1	*P. aeruginosa*
Pseudomonas aeruginosa **Ocean-1175**	Complete Genome	GCF_002237425.1	*P. aeruginosa*
Pseudomonas aeruginosa **PPF-1**	Complete Genome	GCF_002287725.2	*P. aeruginosa*
Pseudomonas aeruginosa **97**	Complete Genome	GCF_002411865.3	*P. aeruginosa*
Pseudomonas aeruginosa **PA 150577**	Complete Genome	GCF_002442855.1	*P. aeruginosa*
Pseudomonas aeruginosa **12939**	Complete Genome	GCF_002753655.1	*P. aeruginosa*
Pseudomonas aeruginosa **PB369**	Complete Genome	GCF_002812825.1	*P. aeruginosa*
Pseudomonas aeruginosa **PB368**	Complete Genome	GCF_002812845.1	*P. aeruginosa*

Pseudomonas aeruginosa **PB353**	Complete Genome	GCF_002812865.1	*P. aeruginosa*
Pseudomonas aeruginosa **PB354**	Complete Genome	GCF_002812885.1	*P. aeruginosa*
Pseudomonas aeruginosa **PB350**	Complete Genome	GCF_002812905.1	*P. aeruginosa*
Pseudomonas aeruginosa **PB367**	Complete Genome	GCF_002812925.1	*P. aeruginosa*
Pseudomonas aeruginosa **F5677**	Complete Genome	GCF_002946935.1	*P. aeruginosa*
Pseudomonas aeruginosa **AR 0360**	Complete Genome	GCF_002968515.1	*P. aeruginosa*
Pseudomonas aeruginosa **AR 0354**	Complete Genome	GCF_002968585.1	*P. aeruginosa*
Pseudomonas aeruginosa **AR 0353**	Complete Genome	GCF_002968655.1	*P. aeruginosa*
Pseudomonas aeruginosa **AR 0230**	Complete Genome	GCF_002968695.1	*P. aeruginosa*
Pseudomonas aeruginosa **AR 0356**	Complete Genome	GCF_002968755.1	*P. aeruginosa*
Pseudomonas aeruginosa **AR 0357**	Complete Genome	GCF_002968955.1	*P. aeruginosa*
Pseudomonas aeruginosa **AR 0095**	Complete Genome	GCF_002997005.1	*P. aeruginosa*
Pseudomonas aeruginosa **CR1**	Complete Genome	GCF_003025345.2	*P. aeruginosa*
Pseudomonas aeruginosa **MRSN12280**	Complete Genome	GCF_003028335.1	*P. aeruginosa*
Pseudomonas aeruginosa **WCHPA075019**	Complete Genome	GCF_003052005.2	*P. aeruginosa*
Pseudomonas aeruginosa **JB2**	Complete Genome	GCF_003060845.1	*P. aeruginosa*
Pseudomonas aeruginosa **AR444**	Complete Genome	GCF_003073615.1	*P. aeruginosa*
Pseudomonas aeruginosa **AR441**	Complete Genome	GCF_003073695.1	*P. aeruginosa*
Pseudomonas aeruginosa **AR445**	Complete Genome	GCF_003073735.1	*P. aeruginosa*
Pseudomonas aeruginosa **AR442**	Complete Genome	GCF_003073795.1	*P. aeruginosa*
Pseudomonas aeruginosa **AR439**	Complete Genome	GCF_003073895.1	*P. aeruginosa*
Pseudomonas aeruginosa **AR 0440**	Chromosome	GCF_003074095.1	*P. aeruginosa*
Pseudomonas aeruginosa **AR 0443**	Chromosome	GCF_003074115.1	*P. aeruginosa*
Pseudomonas aeruginosa **AR 0446**	Complete Genome	GCF_003193645.1	*P. aeruginosa*
Pseudomonas aeruginosa **CCUG 70744**	Complete Genome	GCF_003194245.1	*P. aeruginosa*
Pseudomonas aeruginosa **AR 0110**	Complete Genome	GCF_003204335.1	*P. aeruginosa*
Pseudomonas aeruginosa **K34-7**	Complete Genome	GCF_003206535.1	*P. aeruginosa*
Pseudomonas aeruginosa **AR 460**	Complete Genome	GCF_003288335.1	*P. aeruginosa*

Pseudomonas aeruginosa **AR 455**	Complete Genome	GCF_003288355.1	*P. aeruginosa*
Pseudomonas aeruginosa **AR 458**	Complete Genome	GCF_003288435.1	*P. aeruginosa*
Pseudomonas aeruginosa **HS9**	Complete Genome	GCF_003319235.1	*P. aeruginosa*
Pseudomonas aeruginosa **PA34**	Complete Genome	GCF_003332705.2	*P. aeruginosa*
Pseudomonas aeruginosa **Y89**	Complete Genome	GCF_003369735.1	*P. aeruginosa*
Pseudomonas aeruginosa **Y82**	Complete Genome	GCF_003369755.1	*P. aeruginosa*
Pseudomonas aeruginosa **Y31**	Complete Genome	GCF_003369775.1	*P. aeruginosa*
Pseudomonas aeruginosa **Y71**	Complete Genome	GCF_003408495.1	*P. aeruginosa*
Pseudomonas aeruginosa **PABL048**	Complete Genome	GCF_003411785.2	*P. aeruginosa*
Pseudomonas aeruginosa **PABL012**	Complete Genome	GCF_003429185.1	*P. aeruginosa*
Pseudomonas aeruginosa **PABL017**	Complete Genome	GCF_003429205.1	*P. aeruginosa*
Pseudomonas aeruginosa **24Pae112**	Complete Genome	GCF_003433235.1	*P. aeruginosa*
Pseudomonas aeruginosa **N15-01092**	Complete Genome	GCF_003571505.1	*P. aeruginosa*
Pseudomonas aeruginosa **AR 0111**	Complete Genome	GCF_003571805.1	*P. aeruginosa*
Pseudomonas aeruginosa **BA7823**	Complete Genome	GCF_003626935.1	*P. aeruginosa*
Pseudomonas aeruginosa **268**	Complete Genome	GCF_003641125.1	*P. aeruginosa*
Pseudomonas aeruginosa **6762**	Chromosome	GCF_003665495.1	*P. aeruginosa*
Pseudomonas aeruginosa **SP4528**	Complete Genome	GCF_003716765.1	*P. aeruginosa*
Pseudomonas aeruginosa **BH9**	Chromosome	GCF_003795165.1	*P. aeruginosa*
Pseudomonas aeruginosa **H26027**	Complete Genome	GCF_003798105.1	*P. aeruginosa*
Pseudomonas aeruginosa **H25883**	Complete Genome	GCF_003798125.1	*P. aeruginosa*
Pseudomonas aeruginosa **H26023**	Complete Genome	GCF_003798145.1	*P. aeruginosa*
Pseudomonas aeruginosa **FDAARGOS 532**	Complete Genome	GCF_003812165.1	*P. aeruginosa*
Pseudomonas aeruginosa **FDAARGOS 571**	Complete Genome	GCF_003812885.1	*P. aeruginosa*
Pseudomonas aeruginosa **FDAARGOS 501**	Complete Genome	GCF_003812905.1	*P. aeruginosa*
Pseudomonas aeruginosa **FDAARGOS 505**	Complete Genome	GCF_003813005.1	*P. aeruginosa*
Pseudomonas aeruginosa **FDAARGOS 570**	Complete Genome	GCF_003813025.1	*P. aeruginosa*
Pseudomonas aeruginosa **IMP-13**	Complete Genome	GCF_003950015.1	*P. aeruginosa*

Pseudomonas aeruginosa **SP4371**	Complete Genome	GCF_003950255.1	*P. aeruginosa*
Pseudomonas aeruginosa **SP2230**	Complete Genome	GCF_003952285.1	*P. aeruginosa*
Pseudomonas aeruginosa **B14130**	Complete Genome	GCF_003952305.1	*P. aeruginosa*
Pseudomonas aeruginosa **B17932**	Complete Genome	GCF_003952325.1	*P. aeruginosa*
Pseudomonas aeruginosa **GIMC5015 PAKB6**	Complete Genome	GCF_003957825.1	*P. aeruginosa*
Pseudomonas aeruginosa **SP4527**	Complete Genome	GCF_003991465.1	*P. aeruginosa*
Pseudomonas aeruginosa **GIMC5016 PA1840**	Chromosome	GCF_004000495.1	*P. aeruginosa*
Pseudomonas aeruginosa **LW**	Complete Genome	GCF_004010895.1	*P. aeruginosa*
Pseudomonas aeruginosa **PAO1161**	Complete Genome	GCF_004102665.1	*P. aeruginosa*
Pseudomonas aeruginosa **1334/14**	Complete Genome	GCF_004193735.1	*P. aeruginosa*
Pseudomonas aeruginosa **E80**	Complete Genome	GCF_004291075.1	*P. aeruginosa*
Pseudomonas aeruginosa **PA-VAP-3**	Chromosome	GCF_004346985.1	*P. aeruginosa*
Pseudomonas aeruginosa **PA-VAP-1**	Chromosome	GCF_004347005.1	*P. aeruginosa*
Pseudomonas aeruginosa **PA-VAP-2**	Chromosome	GCF_004347025.1	*P. aeruginosa*
Pseudomonas aeruginosa **PA-VAP-4**	Chromosome	GCF_004347045.1	*P. aeruginosa*
Pseudomonas aeruginosa **AES1M**	Complete Genome	GCF_004355125.1	*P. aeruginosa*
Pseudomonas aeruginosa **AES1R**	Complete Genome	GCF_004355145.1	*P. aeruginosa*
Pseudomonas aeruginosa **AJ D 2**	Chromosome	GCF_004769055.1	*P. aeruginosa*
Pseudomonas aeruginosa **PRD-10**	Chromosome	GCF_005154325.1	*P. aeruginosa*
Pseudomonas aeruginosa **PA298**	Complete Genome	GCF_005305005.1	*P. aeruginosa*
Pseudomonas aeruginosa **FDAARGOS 767**	Complete Genome	GCF_006364735.1	*P. aeruginosa*
Pseudomonas aeruginosa **FDAARGOS 610**	Complete Genome	GCF_006364795.1	*P. aeruginosa*
Pseudomonas aeruginosa **AZPAE15042**	Complete Genome	GCF_006971785.1	*P. aeruginosa*
Pseudomonas aeruginosa **60503**	Complete Genome	GCF_007559065.1	*P. aeruginosa*
Pseudomonas aeruginosa **A681**	Complete Genome	GCF_007559085.1	*P. aeruginosa*
Pseudomonas aeruginosa **519119**	Complete Genome	GCF_007559105.1	*P. aeruginosa*
Pseudomonas aeruginosa **243931**	Complete Genome	GCF_007559125.1	*P. aeruginosa*
Pseudomonas aeruginosa **SCAID WND2-2019**	Chromosome	GCF_007566955.1	*P. aeruginosa*
Pseudomonas aeruginosa **SCAID WND3-2019**	Chromosome	GCF_007567125.1	*P. aeruginosa*
Pseudomonas aeruginosa **SCAID WND1-2019**	Chromosome	GCF_007567285.1	*P. aeruginosa*
Pseudomonas aeruginosa **C79**	Complete Genome	GCF_007833895.1	*P. aeruginosa*
Pseudomonas aeruginosa **IMP67**	Complete Genome	GCF_008033725.1	*P. aeruginosa*

Pseudomonas aeruginosa **IMP68**	Complete Genome	GCF_008033745.1	*P. aeruginosa*
Pseudomonas aeruginosa **IMP66**	Complete Genome	GCF_008033765.1	*P. aeruginosa*
Pseudomonas aeruginosa **CCUG 51971**	Complete Genome	GCF_008195485.1	*P. aeruginosa*
Pseudomonas aeruginosa **HOU1**	Complete Genome	GCF_008245185.1	*P. aeruginosa*
Pseudomonas aeruginosa **GIMC5001 PAT-23**	Chromosome	GCF_008330985.1	*P. aeruginosa*
Pseudomonas aeruginosa **GIMC5002 PAT-169**	Chromosome	GCF_008370085.1	*P. aeruginosa*
Pseudomonas aeruginosa **E90**	Complete Genome	GCF_008705235.1	*P. aeruginosa*
Pseudomonas aeruginosa **PA59**	Complete Genome	GCF_009497675.1	*P. aeruginosa*
Pseudomonas aeruginosa **CFSAN084950**	Complete Genome	GCF_009648875.1	*P. aeruginosa*
Pseudomonas aeruginosa **AG1**	Complete Genome	GCF_009662315.1	*P. aeruginosa*
Pseudomonas aeruginosa **ST773**	Complete Genome	GCF_009664165.1	*P. aeruginosa*
Pseudomonas aeruginosa **1811-13R031**	Complete Genome	GCF_009676765.1	*P. aeruginosa*
Pseudomonas aeruginosa **1811-18R001**	Complete Genome	GCF_009676785.1	*P. aeruginosa*
Pseudomonas aeruginosa **KRP1**	Complete Genome	GCF_009676885.1	*P. aeruginosa*
Pseudomonas aeruginosa **T2436**	Complete Genome	GCF_009720405.1	*P. aeruginosa*
Pseudomonas aeruginosa **T2101**	Complete Genome	GCF_009720425.1	*P. aeruginosa*
Pseudomonas aeruginosa **delta6 2**	Chromosome	GCF_009799985.1	*P. aeruginosa*
Pseudomonas aeruginosa **delta6 3**	Chromosome	GCF_009800005.1	*P. aeruginosa*
Pseudomonas aeruginosa **delta6 4**	Chromosome	GCF_009800025.1	*P. aeruginosa*
Pseudomonas aeruginosa **delta6 5**	Chromosome	GCF_009800045.1	*P. aeruginosa*
Pseudomonas aeruginosa **delta6 6**	Chromosome	GCF_009800065.1	*P. aeruginosa*
Pseudomonas aeruginosa **delta10**	Chromosome	GCF_009800085.1	*P. aeruginosa*
Pseudomonas aeruginosa **Cas9 1**	Chromosome	GCF_009800105.1	*P. aeruginosa*
Pseudomonas aeruginosa **Cas9 2**	Chromosome	GCF_009800125.1	*P. aeruginosa*
Pseudomonas aeruginosa **Environ 1**	Chromosome	GCF_009800145.1	*P. aeruginosa*
Pseudomonas aeruginosa **Environ 2**	Chromosome	GCF_009800165.1	*P. aeruginosa*
Pseudomonas aeruginosa **YB01**	Complete Genome	GCF_009867055.1	*P. aeruginosa*
Pseudomonas aeruginosa **INP-43**	Complete Genome	GCF_009905195.1	*P. aeruginosa*
Pseudomonas aeruginosa **RD1-3**	Complete Genome	GCF_009911735.1	*P. aeruginosa*
Pseudomonas aeruginosa **PAO1 Orsay**	Complete Genome	GCF_900070375.1	*P. aeruginosa*
Pseudomonas aeruginosa **PA14Or reads**	Complete Genome	GCF_900095805.1	*P. aeruginosa*
Pseudomonas aeruginosa **PcyII-10**	Complete Genome	GCF_900149285.1	*P. aeruginosa*

Pseudomonas aeruginosa **RW109**	Complete Genome	GCF_900243355.1	*P. aeruginosa*
Pseudomonas aeruginosa **1**	Complete Genome	GCF_900497025.1	*P. aeruginosa*
Pseudomonas aeruginosa **paerg000**	Complete Genome	GCF_900618245.1	*P. aeruginosa*
Pseudomonas aeruginosa **paerg002**	Complete Genome	GCF_900618255.1	*P. aeruginosa*
Pseudomonas aeruginosa **paerg004**	Complete Genome	GCF_900618265.1	*P. aeruginosa*
Pseudomonas aeruginosa **paerg003**	Complete Genome	GCF_900618275.1	*P. aeruginosa*
Pseudomonas aeruginosa **paerg005**	Complete Genome	GCF_900618285.1	*P. aeruginosa*
Pseudomonas aeruginosa **paerg009**	Complete Genome	GCF_900618305.1	*P. aeruginosa*
Pseudomonas aeruginosa **paerg010**	Complete Genome	GCF_900618315.1	*P. aeruginosa*
Pseudomonas aeruginosa **paerg011**	Complete Genome	GCF_900618325.1	*P. aeruginosa*
Pseudomonas aeruginosa **paerg012**	Complete Genome	GCF_900618335.1	*P. aeruginosa*
Pseudomonas aeruginosa **NCTC11445**	Complete Genome	GCF_900636735.1	*P. aeruginosa*
Pseudomonas aeruginosa **NCTC12903**	Complete Genome	GCF_900636755.1	*P. aeruginosa*
Pseudomonas aeruginosa **NCTC13715**	Complete Genome	GCF_900636975.1	*P. aeruginosa*
Pseudomonas aeruginosa **NCTC10728**	Complete Genome	GCF_900637045.1	*P. aeruginosa*
Pseudomonas aeruginosa **NCTC13359**	Complete Genome	GCF_901472545.1	*P. aeruginosa*
Pseudomonas aeruginosa **NCTC13620**	Complete Genome	GCF_901472565.1	*P. aeruginosa*
Pseudomonas aeruginosa **NCTC13618**	Complete Genome	GCF_901472595.1	*P. aeruginosa*
Pseudomonas aeruginosa **PcyII-29**	Complete Genome	GCF_902703185.1	*P. aeruginosa*
Pseudomonas aeruginosa **PcyII-40**	Complete Genome	GCF_902703195.1	*P. aeruginosa*
Pseudomonas aeruginosa **C7-25**	Complete Genome	GCF_902703215.1	*P. aeruginosa*
Pseudomonas aeruginosa **19BR**	Chromosome	GCF_000223945.1	*P. aeruginosa*
Pseudomonas aeruginosa **213BR**	Chromosome	GCF_000223965.1	*P. aeruginosa*
Pseudomonas aeruginosa **39016**	Chromosome	GCF_000148745.1	*P. aeruginosa*
Pseudomonas aeruginosa **B136-33**	Complete Genome	GCF_000359505.1	*P. aeruginosa*
Pseudomonas aeruginosa **c7447m**	Chromosome	GCF_000468935.2	*P. aeruginosa*
Pseudomonas aeruginosa **C-NN2**	Complete Genome	GCF_900185255.1	*P. aeruginosa*
Pseudomonas aeruginosa **DHS01**	Complete Genome	GCF_000496455.2	*P. aeruginosa*
Pseudomonas aeruginosa **DK1 substr NH57388A**	Complete Genome	GCF_900069025.1	*P. aeruginosa*

Pseudomonas aeruginosa **DK2**	Complete Genome	GCF_000271365.1	*P. aeruginosa*
Pseudomonas aeruginosa **DSM 50071 NBRC 12689**	Complete Genome	GCF_001045685.1	*P. aeruginosa*
Pseudomonas aeruginosa **LES400**	Chromosome	GCF_000583935.1	*P. aeruginosa*
Pseudomonas aeruginosa **LES431**	Complete Genome	GCF_000508765.1	*P. aeruginosa*
Pseudomonas aeruginosa **LESB58**	Complete Genome	GCF_000026645.1	*P. aeruginosa*
Pseudomonas aeruginosa **LESB65**	Chromosome	GCF_000583955.1	*P. aeruginosa*
Pseudomonas aeruginosa **LESlike1**	Chromosome	GCF_000583975.1	*P. aeruginosa*
Pseudomonas aeruginosa **LESlike4**	Chromosome	GCF_000583995.1	*P. aeruginosa*
Pseudomonas aeruginosa **LESlike5**	Chromosome	GCF_000583895.1	*P. aeruginosa*
Pseudomonas aeruginosa **LESlike7**	Chromosome	GCF_000583915.1	*P. aeruginosa*
Pseudomonas aeruginosa **M18**	Complete Genome	GCF_000226155.1	*P. aeruginosa*
Pseudomonas aeruginosa **MTB-1**	Complete Genome	GCF_000504045.1	*P. aeruginosa*
Pseudomonas aeruginosa **NCGM2 S1**	Complete Genome	GCF_000284555.1	*P. aeruginosa*
Pseudomonas aeruginosa **PA1**	Complete Genome	GCF_000496605.2	*P. aeruginosa*
Pseudomonas aeruginosa **PA1R**	Complete Genome	GCF_000496645.1	*P. aeruginosa*
Pseudomonas aeruginosa **PA7**	Complete Genome	GCF_000017205.1	*P. aeruginosa*
Pseudomonas aeruginosa **PA96**	Chromosome	GCF_000626655.2	*P. aeruginosa*
Pseudomonas aeruginosa **PACS2**	Chromosome	GCF_000168335.1	*P. aeruginosa*
Pseudomonas aeruginosa **PAK**	Complete Genome	GCF_902172305.1	*P. aeruginosa*
Pseudomonas aeruginosa **PAO1**	Complete Genome	GCF_000006765.1	*P. aeruginosa*
Pseudomonas aeruginosa **PAO1H2O**	Chromosome	GCF_000714515.1	*P. aeruginosa*
Pseudomonas aeruginosa **PAO1-VE13**	Chromosome	GCF_000484545.2	*P. aeruginosa*
Pseudomonas aeruginosa **PAO1-VE2**	Chromosome	GCF_000484495.2	*P. aeruginosa*
Pseudomonas aeruginosa **PAO581**	Chromosome	GCF_000468555.2	*P. aeruginosa*
Pseudomonas aeruginosa **RP73**	Complete Genome	GCF_000414035.1	*P. aeruginosa*
Pseudomonas aeruginosa **SCV20265**	Complete Genome	GCF_000510305.1	*P. aeruginosa*
Pseudomonas aeruginosa **SJTD-1**	Complete Genome	GCF_000271985.2	*P. aeruginosa*
Pseudomonas aeruginosa **UCBPP-PA14**	Complete Genome	GCF_000014625.1	*P. aeruginosa*
Pseudomonas aeruginosa **VRFPA04**	Complete Genome	GCF_000473745.2	*P. aeruginosa*
Pseudomonas aeruginosa **YL84**	Complete Genome	GCF_000524595.1	*P. aeruginosa*
Pseudomonas agarici **NCPPB 2472**	Chromosome	GCF_001543125.1	*P. asplenii*
Pseudomonas alcaligenes **NEB 585**	Complete Genome	GCF_001597285.1	
Pseudomonas alcaliphila **JAB1**	Complete Genome	GCF_001941865.1	*P. oleovorans*

Pseudomonas alkylphenolica **KL28**	Complete Genome	GCF_000746525.1	*P. putida*
Pseudomonas alkylphenolica **Neo**	Complete Genome	GCF_009755645.1	*P. putida*
Pseudomonas amygdali **pv lachrymans NM002**	Complete Genome	GCF_002068135.1	*P. syringae*
Pseudomonas amygdali **pv lachrymans str M301315**	Complete Genome	GCF_000146005.2	*P. syringae*
Pseudomonas amygdali **pv morsprunorum R15244**	Complete Genome	GCF_002905685.2	*P. syringae*
Pseudomonas amygdali **pv tabaci str ATCC 11528**	Complete Genome	GCF_000145945.2	*P. syringae*
Pseudomonas antarctica **PAMC 27494**	Complete Genome	GCF_001647715.1	*P. fluorescens*
Pseudomonas antarctica **LMG 22709**	Chromosome	GCF_900103795.1	*P. fluorescens*
Pseudomonas arsenicoxydans **CECT 7543**	Chromosome	GCF_900103875.1	*P. mandelii*
Pseudomonas asplenii **ATCC 23835**	Chromosome	GCF_900105475.1	*P. asplenii*
Pseudomonas asturiensis **CC1524**	Complete Genome	GCF_000452745.2	*P. syringae*
Pseudomonas avellanae **R2leaf**	Complete Genome	GCF_002905795.2	*P. syringae*
Pseudomonas azotoformans **S4**	Complete Genome	GCF_001579805.1	*P. fluorescens*
Pseudomonas azotoformans **F77**	Complete Genome	GCF_002007785.1	*P. fluorescens*
Pseudomonas azotoformans **P45A**	Complete Genome	GCF_006547025.1	*P. fluorescens*
Pseudomonas azotoformans **LMG 21611**	Chromosome	GCF_900103345.1	*P. fluorescens*
Pseudomonas balearica **DSM 6083 SP1402**	Complete Genome	GCF_000818015.1	*P. stutzeri*
Pseudomonas brassicacearum **DF41**	Complete Genome	GCF_000585995.1	*P. corrugata*
Pseudomonas brassicacearum **LBUM300**	Complete Genome	GCF_001449085.1	*P. corrugata*
Pseudomonas brassicacearum **L13-6-12**	Complete Genome	GCF_001746815.1	*P. corrugata*
Pseudomonas brassicacearum **3Re2-7**	Complete Genome	GCF_008370715.1	*P. corrugata*
Pseudomonas brassicacearum **S-1**	Complete Genome	GCF_009601685.1	*P. corrugata*
Pseudomonas brassicacearum **LMG 21623**	Chromosome	GCF_900103245.1	*P. corrugata*
Pseudomonas brassicacearum **subsp brassicacearum NFM421**	Complete Genome	GCF_000194805.1	*P. corrugata*
Pseudomonas brenneri **DSM 15294**	Chromosome	GCF_900105815.1	*P. gessardii*
Pseudomonas cedrina **LMG 23661**	Chromosome	GCF_900104915.1	*P. fluorescens*
Pseudomonas cerasi **Sour cherry Prunus cerasus symptomatic leaf**	Complete Genome	GCF_900074915.1	*P. syringae*
Pseudomonas cerasi **PL963**	Complete Genome	GCF_900235895.1	*P. syringae*
Pseudomonas chlororaphis **PA23**	Complete Genome	GCF_000698865.1	*P. chlororaphis*
Pseudomonas chlororaphis **PCL1606**	Complete Genome	GCF_000963835.1	*P. chlororaphis*

Pseudomonas chlororaphis **UFB2**	Complete Genome	GCF_001023535.1	*P. corrugata*
Pseudomonas chlororaphis **189**	Complete Genome	GCF_001602135.1	*P. chlororaphis*
Pseudomonas chlororaphis **Lzh-T5**	Complete Genome	GCF_002844145.1	*P. chlororaphis*
Pseudomonas chlororaphis **TAMOak81**	Complete Genome	GCF_003850605.1	*P. chlororaphis*
Pseudomonas chlororaphis **ATCC 17415**	Complete Genome	GCF_003851145.1	*P. chlororaphis*
Pseudomonas chlororaphis **Pb-St2**	Complete Genome	GCF_003851785.1	*P. chlororaphis*
Pseudomonas chlororaphis **B25**	Complete Genome	GCF_003851985.1	*P. chlororaphis*
Pseudomonas chlororaphis **R47**	Complete Genome	GCF_009834495.1	*P. chlororaphis*
Pseudomonas chlororaphis **ATCC 13985**	Chromosome	GCF_900104715.1	*P. chlororaphis*
Pseudomonas chlororaphis **LMG 21630**	Chromosome	GCF_900104985.1	*P. chlororaphis*
Pseudomonas chlororaphis **DSM 21509**	Chromosome	GCF_900104995.1	*P. chlororaphis*
Pseudomonas chlororaphis **NCTC7357**	Complete Genome	GCF_900636995.1	*P. chlororaphis*
Pseudomonas chlororaphis **O6**	Chromosome	GCF_000264555.1	*P. chlororaphis*
Pseudomonas chlororaphis **subsp aurantiaca JD37**	Complete Genome	GCF_000761195.1	*P. chlororaphis*
Pseudomonas chlororaphis **subsp aurantiaca StFRB508**	Complete Genome	GCF_002355875.1	*P. chlororaphis*
Pseudomonas chlororaphis **subsp aurantiaca M12**	Complete Genome	GCF_003851165.1	*P. chlororaphis*
Pseudomonas chlororaphis **subsp aurantiaca 449**	Complete Genome	GCF_003851205.1	*P. chlororaphis*
Pseudomonas chlororaphis **subsp aurantiaca CW2**	Complete Genome	GCF_003851225.1	*P. chlororaphis*
Pseudomonas chlororaphis **subsp aurantiaca M71**	Complete Genome	GCF_003851265.1	*P. chlororaphis*
Pseudomonas chlororaphis **subsp aurantiaca K27**	Complete Genome	GCF_003851285.1	*P. chlororaphis*
Pseudomonas chlororaphis **subsp aurantiaca PCM 2210**	Complete Genome	GCF_003851305.1	*P. chlororaphis*
Pseudomonas chlororaphis **subsp aurantiaca Q16**	Complete Genome	GCF_003851345.1	*P. chlororaphis*
Pseudomonas chlororaphis **subsp aurantiaca 464**	Complete Genome	GCF_003851805.1	*P. chlororaphis*
Pseudomonas chlororaphis **subsp aurantiaca DSM 19603**	Complete Genome	GCF_003851835.1	*P. chlororaphis*
Pseudomonas chlororaphis **subsp aurantiaca ARS 38**	Complete Genome	GCF_009362955.1	*P. chlororaphis*
Pseudomonas chlororaphis **subsp aurantiaca zm-1**	Complete Genome	GCF_010092525.1	*P. chlororaphis*
Pseudomonas chlororaphis **subsp aureofaciens P2**	Complete Genome	GCF_003851365.1	*P. chlororaphis*
Pseudomonas chlororaphis **subsp aureofaciens C50**	Complete Genome	GCF_003851385.1	*P. chlororaphis*
Pseudomonas chlororaphis **subsp aureofaciens 66**	Complete Genome	GCF_003851405.1	*P. chlororaphis*

***Pseudomonas chlororaphis* subsp aureofaciens ChPhzS23**	Complete Genome	GCF_003851425.1	*P. chlororaphis*
***Pseudomonas chlororaphis* subsp aureofaciens ChPhzS24**	Complete Genome	GCF_003851445.1	*P. chlororaphis*
***Pseudomonas chlororaphis* subsp aureofaciens ChPhzTR36**	Complete Genome	GCF_003851865.1	*P. chlororaphis*
***Pseudomonas chlororaphis* subsp aureofaciens DSM 6698**	Complete Genome	GCF_003851905.1	*P. chlororaphis*
***Pseudomonas chlororaphis* subsp aureofaciens ChPhzTR39**	Complete Genome	GCF_003851925.1	*P. chlororaphis*
***Pseudomonas chlororaphis* subsp aureofaciens ChPhzTR18**	Complete Genome	GCF_003851955.1	*P. chlororaphis*
***Pseudomonas chlororaphis* subsp aureofaciens ChPhzTR38**	Complete Genome	GCF_003852005.1	*P. chlororaphis*
***Pseudomonas chlororaphis* subsp aureofaciens 30-84 30-84**	Chromosome	GCF_000281915.1	*P. chlororaphis*
***Pseudomonas chlororaphis* subsp chlororaphis DSM 50083**	Complete Genome	GCF_003945765.1	*P. chlororaphis*
***Pseudomonas chlororaphis* subsp piscium ZJU60**	Complete Genome	GCF_003008635.1	*P. chlororaphis*
***Pseudomonas chlororaphis* subsp piscium DSM 21509**	Complete Genome	GCF_003850345.1	*P. chlororaphis*
***Pseudomonas chlororaphis* subsp piscium ATCC 17809**	Complete Genome	GCF_003850365.1	*P. chlororaphis*
***Pseudomonas chlororaphis* subsp piscium ATCC 17411**	Complete Genome	GCF_003850385.1	*P. chlororaphis*
***Pseudomonas chlororaphis* subsp piscium SLPH10**	Complete Genome	GCF_003850405.1	*P. chlororaphis*
***Pseudomonas chlororaphis* subsp piscium DTR133**	Complete Genome	GCF_003850425.1	*P. chlororaphis*
***Pseudomonas chlororaphis* subsp piscium PCL1391**	Complete Genome	GCF_003850445.1	*P. chlororaphis*
***Pseudomonas chlororaphis* subsp piscium PCL1607**	Complete Genome	GCF_003850465.1	*P. chlororaphis*
***Pseudomonas chlororaphis* subsp piscium ChPhzS135**	Complete Genome	GCF_003850485.1	*P. chlororaphis*
***Pseudomonas chlororaphis* subsp piscium ChPhzS140**	Complete Genome	GCF_003850505.1	*P. chlororaphis*
***Pseudomonas chlororaphis* subsp piscium ChPhzTR44**	Complete Genome	GCF_003850525.1	*P. chlororaphis*
***Pseudomonas chlororaphis* subsp piscium ToZa7**	Complete Genome	GCF_003850585.1	*P. chlororaphis*
***Pseudomonas cichorii* JBC1**	Complete Genome	GCF_000517305.1	*P. syringae*
***Pseudomonas citronellolis* P3B5**	Complete Genome	GCF_001586155.1	*P. nitroreducens*
***Pseudomonas citronellolis* SJTE-3**	Complete Genome	GCF_001654435.1	*P. nitroreducens*
***Pseudomonas coronafaciens* pv oryzae str 1 6**	Complete Genome	GCF_000156995.2	*P. syringae*
***Pseudomonas corrugata* RM1-1-4**	Complete Genome	GCF_001708425.1	*P. corrugata*
***Pseudomonas corrugata* LMG 2172**	Chromosome	GCF_900106085.1	*P. corrugata*
***Pseudomonas cremoricolorata* ND07**	Complete Genome	GCF_000759535.1	*P. putida*

Pseudomonas denitrificans **nomen rejiciendum BG1**	Complete Genome	GCF_008807415.1	*P. nitroreducens*
Pseudomonas entomophila **2014**	Complete Genome	GCF_003940785.1	*P. putida*
Pseudomonas entomophila **1257**	Complete Genome	GCF_003940825.1	*P. putida*
Pseudomonas entomophila **L48 L48**	Complete Genome	GCF_000026105.1	*P. putida*
Pseudomonas extremaustralis **DSM 17835**	Chromosome	GCF_900102035.1	*P. fluorescens*
Pseudomonas extremorientalis **LMG 19695**	Chromosome	GCF_900104365.1	*P. fluorescens*
Pseudomonas fluorescens **UK4**	Complete Genome	GCF_000730425.1	*P. gessardii*
Pseudomonas fluorescens **FW300-N2E3**	Complete Genome	GCF_001307155.1	
Pseudomonas fluorescens **FW300-N2C3**	Complete Genome	GCF_001307275.1	*P. corrugata*
Pseudomonas fluorescens **FW300-N2E2**	Chromosome	GCF_001623525.1	*P. corrugata*
Pseudomonas fluorescens **L321**	Complete Genome	GCF_001708445.1	*P. fluorescens*
Pseudomonas fluorescens **L111**	Complete Genome	GCF_001708465.1	*P. fluorescens*
Pseudomonas fluorescens **Pt14**	Complete Genome	GCF_001747385.1	*P. fluorescens*
Pseudomonas fluorescens **2P24**	Complete Genome	GCF_002865505.1	*P. corrugata*
Pseudomonas fluorescens **FR1**	Chromosome	GCF_002879715.1	*P. corrugata*
Pseudomonas fluorescens **MS82**	Complete Genome	GCF_003055645.1	*P. koreensis*
Pseudomonas fluorescens **NEP1**	Chromosome	GCF_003352875.1	*P. koreensis*
Pseudomonas fluorescens **Pf275**	Complete Genome	GCF_003410335.1	*P. corrugata*
Pseudomonas fluorescens **SIK W1**	Complete Genome	GCF_003612935.1	*P. fluorescens*
Pseudomonas fluorescens **PF08**	Complete Genome	GCF_003626995.1	*P. gessardii*
Pseudomonas fluorescens **LBUM677**	Complete Genome	GCF_004683905.1	*P. koreensis*
Pseudomonas fluorescens **ATCC 13525**	Chromosome	GCF_900215245.1	*P. fluorescens*
Pseudomonas fluorescens **NCTC10038**	Complete Genome	GCF_900475215.1	*P. fluorescens*
Pseudomonas fluorescens **NCTC10783**	Complete Genome	GCF_900636635.1	*P. aeruginosa*
Pseudomonas fluorescens **NCTC9428**	Complete Genome	GCF_900636825.1	*P. koreensis*
Pseudomonas fluorescens **A506**	Complete Genome	GCF_000262325.2	*P. fluorescens*
Pseudomonas fluorescens **F113**	Complete Genome	GCF_000237065.1	*P. corrugata*
Pseudomonas fluorescens **NCIMB 11764**	Complete Genome	GCF_000293885.2	*P. mandelii*
Pseudomonas fluorescens **Pf0-1**	Complete Genome	GCF_000012445.1	*P. koreensis*
Pseudomonas fluorescens **Q2-87**	Chromosome	GCF_000281895.1	*P. corrugata*
Pseudomonas fluorescens **Q8r1-96**	Chromosome	GCF_000263695.1	*P. corrugata*

***Pseudomonas fluorescens* R124**	Chromosome	GCF_000292795.1	*P. koreensis*
***Pseudomonas fluorescens* SBW25**	Complete Genome	GCF_000009225.2	*P. fluorescens*
***Pseudomonas fluorescens* WH6**	Chromosome	GCF_000166515.1	*P. fluorescens*
***Pseudomonas fragi* P121**	Complete Genome	GCF_001543265.1	*P. fragi*
***Pseudomonas fragi* NMC25**	Complete Genome	GCF_002128325.1	*P. fragi*
***Pseudomonas fragi* DBC**	Chromosome	GCF_002205455.1	*P. fragi*
***Pseudomonas fragi* NRRL B-727**	Chromosome	GCF_900105835.1	*P. fragi*
***Pseudomonas frederiksbergensis* ERDD5 01**	Complete Genome	GCF_001874645.1	
***Pseudomonas frederiksbergensis* AS1**	Complete Genome	GCF_001952935.1	*P. mandelii*
***Pseudomonas frederiksbergensis* KNU-15**	Complete Genome	GCF_002355315.1	*P. mandelii*
***Pseudomonas fulva* SB1**	Complete Genome	GCF_002688705.1	*P. putida*
***Pseudomonas fulva* FDAARGOS 167**	Complete Genome	GCF_002951475.1	*P. putida*
***Pseudomonas fulva* 12-X**	Complete Genome	GCF_000213805.1	
***Pseudomonas furukawaii* KF707**	Complete Genome	GCF_002355475.1	*P. resinovorans*
***Pseudomonas fuscovaginae* LMG 2158**	Chromosome	GCF_900108595.1	*P. asplenii*
***Pseudomonas granadensis* LMG 27940**	Chromosome	GCF_900105485.1	*P. koreensis*
***Pseudomonas guangdongensis* CCTCC AB 2012022**	Chromosome	GCF_900105885.1	
***Pseudomonas knackmussii* N1-2**	Complete Genome	GCF_009911755.1	*P. nitroreducens*
***Pseudomonas knackmussii* B13**	Complete Genome	GCF_000689415.1	*P. nitroreducens*
***Pseudomonas koreensis* D26**	Complete Genome	GCF_001605965.1	*P. koreensis*
***Pseudomonas koreensis* CRS05-R5**	Complete Genome	GCF_001654515.1	*P. koreensis*
***Pseudomonas koreensis* LMG 21318**	Chromosome	GCF_900101415.1	*P. koreensis*
***Pseudomonas kribbensis* 46-2**	Complete Genome	GCF_003352185.1	*P. koreensis*
***Pseudomonas lactis* SS101**	Chromosome	GCF_000263675.1	*P. fluorescens*
***Pseudomonas libanensis* DMSP-1**	Complete Genome	GCF_003952245.1	*P. fluorescens*
***Pseudomonas libanensis* DSM 17149**	Chromosome	GCF_900101035.1	*P. fluorescens*
***Pseudomonas lini* DSM 16768**	Chromosome	GCF_900104735.1	*P. mandelii*
***Pseudomonas litoralis* 2SM5**	Chromosome	GCF_900105005.1	*P. pertucinogena*
***Pseudomonas lundensis* AU1044**	Complete Genome	GCF_001020725.2	*P. fragi*
***Pseudomonas lurida* L228**	Complete Genome	GCF_001708485.1	*P. fluorescens*
***Pseudomonas lurida* MYb11**	Complete Genome	GCF_002966835.1	*P. fluorescens*
***Pseudomonas luteola* FDAARGOS 637**	Complete Genome	GCF_008693965.1	

***Pseudomonas mandelii* LMG 21607**	Chromosome	GCF_900106065.1	*P. mandelii*
***Pseudomonas mandelii* JR-1**	Complete Genome	GCF_000257545.3	*P. mandelii*
***Pseudomonas marincola* YSy11**	Complete Genome	GCF_900682675.1	
***Pseudomonas mediterranea* DSM 16733**	Chromosome	GCF_900106005.1	*P. corrugata*
***Pseudomonas mendocina* NSYSU**	Complete Genome	GCF_002155165.1	*P. oleovorans*
***Pseudomonas mendocina* MAE1-K**	Complete Genome	GCF_002442555.1	*P. oleovorans*
***Pseudomonas mendocina* NEB698**	Complete Genome	GCF_003008615.1	*P. oleovorans*
***Pseudomonas mendocina* NCTC10897**	Complete Genome	GCF_900636545.1	*P. oleovorans*
***Pseudomonas mendocina* NK-01**	Complete Genome	GCF_000204295.1	*P. oleovorans*
***Pseudomonas mendocina* S5 2**	Complete Genome	GCF_000733715.2	*P. oleovorans*
***Pseudomonas mendocina* ymp**	Complete Genome	GCF_000016565.1	*P. oleovorans*
***Pseudomonas monteilii* USDA-ARS-USMARC-56711**	Complete Genome	GCF_001534745.1	*P. putida*
***Pseudomonas monteilii* FDAARGOS 171**	Complete Genome	GCF_002943995.1	*P. putida*
***Pseudomonas monteilii* B5**	Complete Genome	GCF_003671975.1	*P. putida*
**Pseudomonas monteilii TCU-CK1**	Complete Genome	GCF_009831395.1	*P. putida*
***Pseudomonas monteilii* SB3078**	Complete Genome	GCF_000510285.1	*P. putida*
***Pseudomonas monteilii* SB3101**	Complete Genome	GCF_000510325.1	*P. putida*
***Pseudomonas moraviensis* LMG 24280**	Chromosome	GCF_900105805.1	*P. koreensis*
***Pseudomonas moraviensis* R28-S**	Chromosome	GCF_000512275.1	*P. koreensis*
***Pseudomonas mosselii* BS011**	Complete Genome	GCF_002309555.1	*P. putida*
***Pseudomonas mosselii* PtA1**	Complete Genome	GCF_002736065.1	*P. putida*
***Pseudomonas mucidolens* LMG 2223**	Chromosome	GCF_900106045.1	*P. gessardii*
***Pseudomonas mucidolens* NCTC8068**	Complete Genome	GCF_900475945.1	*P. gessardii*
Pseudomonas oleovorans	Complete Genome	GCF_000953455.1	*P. oleovorans*
***Pseudomonas oleovorans* T9AD**	Complete Genome	GCF_900626055.1	*P. oleovorans*
***Pseudomonas orientalis* F9**	Complete Genome	GCF_002934065.1	*P. fluorescens*
***Pseudomonas orientalis* L1-3-08**	Complete Genome	GCF_003851585.1	*P. fluorescens*
***Pseudomonas orientalis* R2-66-08W**	Complete Genome	GCF_003851605.1	*P. fluorescens*
***Pseudomonas orientalis* R4-35-08**	Complete Genome	GCF_003851645.1	*P. fluorescens*
***Pseudomonas orientalis* 8B**	Complete Genome	GCF_003852045.1	*P. fluorescens*

***Pseudomonas orientalis* LMG 23660**	Chromosome	GCF_900105795.1	*P. fluorescens*
***Pseudomonas oryzae* KCTC 32247**	Chromosome	GCF_900104805.1	
***Pseudomonas oryzihabitans* USDA-ARS-USMARC-56511**	Complete Genome	GCF_001518815.1	*P. oryzihabitans*
***Pseudomonas oryzihabitans* FDAARGOS 657**	Complete Genome	GCF_008693825.1	*P. oryzihabitans*
***Pseudomonas palleroniana* MAB3**	Chromosome	GCF_002953635.1	*P. fluorescens*
***Pseudomonas parafulva* CRS01-1**	Complete Genome	GCF_000800255.1	*P. putida*
***Pseudomonas parafulva* PRS09-11288**	Complete Genome	GCF_002021815.1	*P. putida*
***Pseudomonas parafulva* JBCS1880**	Complete Genome	GCF_003410295.1	*P. putida*
***Pseudomonas pelagia* Kongs-67**	Complete Genome	GCF_009497895.1	*P. pertucinogena*
***Pseudomonas plecoglossicida* NyZ12**	Complete Genome	GCF_000831585.1	*P. putida*
***Pseudomonas plecoglossicida* XSDHY-P**	Complete Genome	GCF_003391255.1	*P. putida*
***Pseudomonas poae* LMG 21465**	Chromosome	GCF_900103965.1	*P. fluorescens*
***Pseudomonas poae* RE1-1-14**	Complete Genome	GCF_000336465.1	*P. fluorescens*
***Pseudomonas pohangensis* DSM 17875**	Chromosome	GCF_900105995.1	
***Pseudomonas prosekii* LMG 26867**	Chromosome	GCF_900105155.1	*P. mandelii*
***Pseudomonas protegens* UCT**	Chromosome	GCF_001874665.1	*P. protegens*
***Pseudomonas protegens* H78**	Complete Genome	GCF_002006545.1	*P. protegens*
***Pseudomonas protegens* FDAARGOS 307**	Complete Genome	GCF_002208745.2	*P. protegens*
***Pseudomonas protegens* FD6**	Complete Genome	GCF_003363755.1	*P. protegens*
***Pseudomonas protegens* pf5**	Complete Genome	GCF_008807675.1	*P. protegens*
**Pseudomonas protegens pf5-k3**	Complete Genome	GCF_008807695.1	*P. protegens*
***Pseudomonas protegens* pf5-k2**	Complete Genome	GCF_008807715.1	*P. protegens*
***Pseudomonas protegens* SN15-2**	Complete Genome	GCF_009745915.1	*P. protegens*
***Pseudomonas protegens* Cab57**	Complete Genome	GCF_000828695.1	*P. protegens*
***Pseudomonas protegens* CHA0**	Complete Genome	GCF_000397205.1	*P. protegens*
***Pseudomonas protegens* Pf-5**	Complete Genome	GCF_000012265.1	*P. protegens*
***Pseudomonas pseudoalcaligenes* CECT 5344**	Complete Genome	GCF_000297075.2	*P. oleovorans*
***Pseudomonas psychrophila* LMG 24276**	Chromosome	GCF_900106105.1	*P. fragi*
***Pseudomonas psychrotolerans* PRS08-11306**	Complete Genome	GCF_001913135.1	*P. oryzihabitans*
***Pseudomonas psychrotolerans* CS51**	Complete Genome	GCF_006384975.1	*P. oryzihabitans*
***Pseudomonas putida* DLL-E4**	Complete Genome	GCF_000691565.1	*P. putida*

***Pseudomonas putida* 1A00316**	Complete Genome	GCF_001515585.1	*P. putida*
***Pseudomonas putida* PC2**	Complete Genome	GCF_001636055.1	*P. putida*
***Pseudomonas putida* PP112420**	Complete Genome	GCF_001886975.1	*P. putida*
***Pseudomonas putida* DRA525**	Chromosome	GCF_001908395.1	*P. putida*
***Pseudomonas putida* AA7**	Complete Genome	GCF_002025705.1	*P. putida*
***Pseudomonas putida* KF715**	Complete Genome	GCF_002356095.1	*P. putida*
***Pseudomonas putida* E46**	Complete Genome	GCF_002736045.1	*P. putida*
***Pseudomonas putida* E41**	Complete Genome	GCF_002736125.1	*P. putida*
***Pseudomonas putida* IEC33019**	Complete Genome	GCF_002741075.1	*P. putida*
***Pseudomonas putida* JBC17**	Complete Genome	GCF_003228315.1	*P. jessenii*
***Pseudomonas putida* NX-1**	Complete Genome	GCF_003290365.1	*P. putida*
***Pseudomonas putida* B4**	Complete Genome	GCF_003671955.1	*P. putida*
***Pseudomonas putida* 1290**	Complete Genome	GCF_005080685.1	*P. putida*
***Pseudomonas putida* B1**	Complete Genome	GCF_007833495.1	*P. putida*
***Pseudomonas putida* JYR-1**	Complete Genome	GCF_008605605.1	*P. putida*
***Pseudomonas putida* W5**	Complete Genome	GCF_009883635.1	*P. putida*
***Pseudomonas putida* TS312**	Complete Genome	GCF_009938035.1	*P. putida*
***Pseudomonas putida* N1R**	Chromosome	GCF_900156185.1	*P. putida*
***Pseudomonas putida* NCTC13186**	Complete Genome	GCF_900636645.1	*P. putida*
***Pseudomonas putida* B6-2**	Complete Genome	GCF_000226035.2	*P. putida*
***Pseudomonas putida* BIRD-1**	Complete Genome	GCF_000183645.1	*P. putida*
***Pseudomonas putida* DOT-T1E**	Complete Genome	GCF_000281215.1	*P. putida*
***Pseudomonas putida* F1**	Complete Genome	GCF_000016865.1	*P. putida*
***Pseudomonas putida* GB-1**	Complete Genome	GCF_000019125.1	*P. putida*
***Pseudomonas putida* H8234**	Complete Genome	GCF_000410575.1	*P. putida*
***Pseudomonas putida* HB3267 PC9**	Complete Genome	GCF_000325725.1	*P. putida*
***Pseudomonas putida* JB**	Complete Genome	GCF_001767335.1	*P. putida*
***Pseudomonas putida* KT2440**	Complete Genome	GCF_000007565.2	*P. putida*

Pseudomonas putida **NBRC 14164**	Complete Genome	GCF_000412675.1	*P. putida*
Pseudomonas putida **ND6**	Complete Genome	GCF_000264665.2	*P. putida*
Pseudomonas putida **S12**	Complete Genome	GCF_000495455.2	*P. putida*
Pseudomonas putida **S13 1 2**	Complete Genome	GCF_000498395.2	*P. putida*
Pseudomonas putida **S16**	Complete Genome	GCF_000219705.1	*P. putida*
Pseudomonas putida **SJTE-1**	Complete Genome	GCF_000271965.2	*P. putida*
Pseudomonas putida **W619**	Complete Genome	GCF_000019445.1	*P. putida*
Pseudomonas reinekei **DSM 18361**	Chromosome	GCF_900104125.1	*P. jessenii*
Pseudomonas resinovorans **NBRC 106553**	Complete Genome	GCF_000412695.1	*P. resinovorans*
Pseudomonas rhizosphaerae **DSM 16299**	Complete Genome	GCF_000761155.1	
Pseudomonas rhodesiae **LMG 17764**	Chromosome	GCF_900105575.1	*P. fluorescens*
Pseudomonas sabulinigri **JCM 14963**	Chromosome	GCF_900105255.1	*P. pertucinogena*
Pseudomonas salegens **CECT 8338**	Chromosome	GCF_900105655.1	*P. pertucinogena*
Pseudomonas saudiphocaensis **20 BN**	Chromosome	GCF_000756775.1	*P. stutzeri*
Pseudomonas savastanoi **pv phaseolicola 1448A BAA-978**	Complete Genome	GCF_000012205.1	*P. syringae*
Pseudomonas savastanoi **pv savastanoi NCPPB 3335**	Complete Genome	GCF_000164015.3	*P. syringae*
Pseudomonas sihuiensis **KCTC 32246**	Chromosome	GCF_900106015.1	*P. oleovorans*
Pseudomonas silesiensis **A3**	Complete Genome	GCF_001661075.1	*P. mandelii*
Pseudomonas simiae **WCS417**	Chromosome	GCF_000698265.1	*P. fluorescens*
Pseudomonas simiae **PCL1751**	Complete Genome	GCF_000934565.1	*P. fluorescens*
Pseudomonas simiae **PICF7**	Complete Genome	GCF_000963495.1	*P. fluorescens*
Pseudomonas soli **SJ10**	Complete Genome	GCF_000498975.2	*P. putida*
Pseudomonas stutzeri **CGMCC 1 1803**	Complete Genome	GCF_000219605.1	*P. stutzeri*
Pseudomonas stutzeri **28a24**	Complete Genome	GCF_000590475.1	*P. stutzeri*
Pseudomonas stutzeri **19SMN4**	Complete Genome	GCF_000661915.1	*P. stutzeri*
Pseudomonas stutzeri **SLG510A3-8**	Complete Genome	GCF_001038645.1	*P. stutzeri*
Pseudomonas stutzeri **273**	Complete Genome	GCF_001648195.1	*P. stutzeri*
Pseudomonas stutzeri **DW2-1**	Complete Genome	GCF_003001655.1	*P. stutzeri*
Pseudomonas stutzeri **1W1-1A**	Complete Genome	GCF_003008495.1	*P. stutzeri*
Pseudomonas stutzeri **SGAir0442**	Complete Genome	GCF_003047145.2	*P. stutzeri*

Pseudomonas stutzeri **PheN2**	Complete Genome	GCF_005844005.1	*P. stutzeri*
Pseudomonas stutzeri **XL272**	Complete Genome	GCF_009740185.1	*P. stutzeri*
Pseudomonas stutzeri **PM101005**	Complete Genome	GCF_009789555.1	*P. stutzeri*
Pseudomonas stutzeri **NCTC10450**	Complete Genome	GCF_900636845.1	*P. stutzeri*
Pseudomonas stutzeri **NCTC10475**	Complete Genome	GCF_900638035.1	*P. stutzeri*
Pseudomonas stutzeri **A1501**	Complete Genome	GCF_000013785.1	*P. stutzeri*
Pseudomonas stutzeri **CCUG 29243**	Complete Genome	GCF_000267545.1	*P. stutzeri*
Pseudomonas stutzeri **DSM 10701**	Complete Genome	GCF_000279165.1	*P. stutzeri*
Pseudomonas stutzeri **DSM 4166**	Complete Genome	GCF_000195105.1	*P. stutzeri*
Pseudomonas stutzeri **RCH2**	Complete Genome	GCF_000327065.1	*P. stutzeri*
Pseudomonas synxantha **LBUM223**	Complete Genome	GCF_000968415.2	*P. fluorescens*
Pseudomonas synxantha **KENGFT3**	Chromosome	GCF_001602175.1	*P. fluorescens*
Pseudomonas synxantha **30B**	Complete Genome	GCF_003851465.1	*P. fluorescens*
Pseudomonas synxantha **28887**	Complete Genome	GCF_003851495.1	*P. fluorescens*
Pseudomonas synxantha **R2-4-08W**	Complete Genome	GCF_003851525.1	*P. fluorescens*
Pseudomonas synxantha **R6-28-08**	Complete Genome	GCF_003851555.1	*P. fluorescens*
Pseudomonas synxantha **R2-54-08W**	Complete Genome	GCF_003852025.1	*P. fluorescens*
Pseudomonas synxantha **LMG 2190**	Chromosome	GCF_900105675.1	*P. fluorescens*
Pseudomonas synxantha **NCTC10696**	Complete Genome	GCF_901482615.1	*P. fluorescens*
Pseudomonas synxantha **BG33R**	Chromosome	GCF_000263715.2	*P. fluorescens*
Pseudomonas syringae **inb918**	Complete Genome	GCF_004006335.1	
Pseudomonas syringae **Ps25**	Chromosome	GCF_004323795.1	*P. syringae*
Pseudomonas syringae **31R1**	Chromosome	GCF_900105295.1	*P. syringae*
Pseudomonas syringae **CFBP3840**	Complete Genome	GCF_900235815.1	*P. syringae*
Pseudomonas syringae **CC1557**	Complete Genome	GCF_000452705.1	*P. syringae*
Pseudomonas syringae **group genomosp 3 CFBP6411**	Complete Genome	GCF_900235905.1	*P. syringae*
Pseudomonas syringae **pv actinidiae ICMP 20586**	Complete Genome	GCF_001913215.1	*P. syringae*
Pseudomonas syringae **pv actinidiae NZ-47**	Complete Genome	GCF_001913235.1	*P. syringae*
Pseudomonas syringae **pv actinidiae CRAFRU 12 29**	Complete Genome	GCF_002024285.1	*P. syringae*
Pseudomonas syringae **pv actinidiae CRAFRU 14 08**	Complete Genome	GCF_002024305.1	*P. syringae*

***Pseudomonas syringae* pv actinidiae MAFF212063**	Complete Genome	GCF_002763655.1	*P. syringae*
***Pseudomonas syringae* pv actinidiae P155**	Complete Genome	GCF_003665415.1	*P. syringae*
***Pseudomonas syringae* pv actinidiae ICMP 18708**	Complete Genome	GCF_000344355.2	*P. syringae*
***Pseudomonas syringae* pv actinidiae ICMP 18884**	Complete Genome	GCF_000648735.3	*P. syringae*
***Pseudomonas syringae* pv actinidiae ICMP 9617**	Chromosome	GCF_000658965.1	*P. syringae*
***Pseudomonas syringae* pv actinidiae ICMP 9853**	Complete Genome	GCF_000344335.2	*P. syringae*
***Pseudomonas syringae* pv actinidiae str Shaanxi M228**	Complete Genome	GCF_000344475.3	*P. syringae*
***Pseudomonas syringae* pv atrofaciens LMG5095**	Complete Genome	GCF_003047185.1	*P. syringae*
***Pseudomonas syringae* pv avii CFBP3846**	Complete Genome	GCF_900235835.1	*P. syringae*
***Pseudomonas syringae* pv cerasicola CFBP6109**	Complete Genome	GCF_900235885.1	*P. syringae*
***Pseudomonas syringae* pv lapsa ATCC 10859**	Complete Genome	GCF_001482725.1	*P. syringae*
***Pseudomonas syringae* pv maculicola str ES4326**	Complete Genome	GCF_000145845.2	*P. syringae*
***Pseudomonas syringae* pv pisi str PP1**	Complete Genome	GCF_000452445.2	*P. syringae*
***Pseudomonas syringae* pv syringae Pss9097**	Complete Genome	GCF_002905815.2	*P. syringae*
***Pseudomonas syringae* pv syringae CFBP4215**	Complete Genome	GCF_900235825.1	*P. syringae*
***Pseudomonas syringae* pv syringae CFBP2118**	Complete Genome	GCF_900235865.1	*P. syringae*
***Pseudomonas syringae* pv syringae B301D**	Complete Genome	GCF_000988485.1	*P. syringae*
***Pseudomonas syringae* pv syringae B64**	Chromosome	GCF_000331385.1	*P. syringae*
***Pseudomonas syringae* pv syringae B728a**	Complete Genome	GCF_000012245.1	*P. syringae*
***Pseudomonas syringae* pv syringae HS191**	Complete Genome	GCF_000988395.1	*P. syringae*
***Pseudomonas syringae* pv syringae SM**	Chromosome	GCF_000412165.1	*P. syringae*
***Pseudomonas syringae* pv tomato B13-200**	Complete Genome	GCF_002966555.1	*P. syringae*
***Pseudomonas syringae* pv tomato delta VI**	Chromosome	GCF_009800185.1	*P. syringae*
***Pseudomonas syringae* pv tomato delta IV,IX**	Chromosome	GCF_009800205.1	*P. syringae*
***Pseudomonas syringae* pv tomato delta X**	Complete Genome	GCF_009800225.1	*P. syringae*
***Pseudomonas syringae* pv tomato DC3000**	Complete Genome	GCF_000007805.1	*P. syringae*
***Pseudomonas syringae* UB303**	Complete Genome	GCF_000452565.2	*P. syringae*
***Pseudomonas syringae* UMAF0158**	Complete Genome	GCF_001281365.1	*P. syringae*
***Pseudomonas syringae* USA011**	Complete Genome	GCF_000452525.3	*P. syringae*
***Pseudomonas taetrolens* NCTC10697**	Complete Genome	GCF_900475285.1	*P. fragi*

***Pseudomonas taetrolens* NCTC8067**	Complete Genome	GCF_900637735.1	*P. fragi*
***Pseudomonas thivervalensis* DSM 13194**	Chromosome	GCF_900102295.1	*P. corrugata*
***Pseudomonas tolaasii* 2192T**	Chromosome	GCF_002072675.1	*P. fluorescens*
***Pseudomonas trivialis* IHBB745**	Complete Genome	GCF_001186335.1	*P. fluorescens*
***Pseudomonas trivialis* LMG 21464**	Chromosome	GCF_900104885.1	*P. fluorescens*
***Pseudomonas umsongensis* GO16**	Chromosome	GCF_008824165.1	*P. jessenii*
***Pseudomonas umsongensis* LMG 21317**	Chromosome	GCF_900105285.1	*P. jessenii*
***Pseudomonas vancouverensis* LMG 20222**	Chromosome	GCF_900105825.1	*P. jessenii*
***Pseudomonas veronii* R02**	Complete Genome	GCF_002028325.1	*P. fluorescens*
***Pseudomonas veronii* Pvy**	Complete Genome	GCF_004919535.2	*P. fluorescens*
***Pseudomonas versuta* L10 10**	Complete Genome	GCF_001294575.1	*P. fragi*
***Pseudomonas viridiflava* CFBP 1590**	Complete Genome	GCF_900184295.1	*P. syringae*
***Pseudomonas xanthomarina* LMG 23572**	Chromosome	GCF_900108535.1	*P. stutzeri*
***Pseudomonas xinjiangensis* NRRL B-51270**	Chromosome	GCF_900104945.1	*P. pertucinogena*
***Pseudomonas yamanorum* LBUM636**	Complete Genome	GCF_001612705.2	*P. yamanorum*
***Pseudomonas yamanorum* LMG 27247**	Chromosome	GCF_900105735.1	*P. yamanorum*
