# Supplementary material for: In Silico Characterization and Phylogenetic Distribution of Extracellular Matrix Components in the Model Rhizobacteria Pseudomonas fluorescens F113 and Other Pseudomonads
Source: Microorganisms. 2020 Nov 6;8(11):1740. doi: 10.3390/microorganisms8111740 (PMC7716237; doi:10.3390/microorganisms8111740)
Supplement: Supplementary file 1 [file microorganisms-08-01740-s001.zip › Supplementary_Table_4.pdf]

Supplementary Table 4. Predicted localization, domains, and function of *Pseudomonas* acidic polysaccharide (Pap) biosynthetic proteins in *Pseudomonas fluorescens* F113.

Locus (PSF113_)	Protein	Length	Predicted cellular localization			Predicted transmembrane domains (HMMER and TMHMM2)		Predicted domains			Predicted function
			Localization	CELLO Score	Psortb Score	Number	Position	Domain	Position	SMART e-value	
**1970**	**PapA**	454	**Cytoplasmic**	4,34	9,97	0	-	UDP-glucose/GDP-mannose dehydrogenase family, NAD binding domain	1-188	6,4E-61	UDP-glucose 6-dehydrogenase
								UDP-glucose/GDP-mannose dehydrogenase family, central domain	205-298	4,8E-40	
								UDP-glucose/GDP-mannose dehydrogenase family, UDP binding domain	321-425	3,28E-47	
**1969**	**PapB**	352	**Cytoplasmic**	4,719	9,97	0	-	NAD dependent epimerase/dehydratase family	3-263	1,8E-56	NAD-dependent epimerase/dehydratase
								GDP-mannose 4,6 dehydratase	4-326	9,7E-54	
								RmlD substrate binding domain	1-246	1,5E-19	
								3-beta hydroxysteroid dehydrogenase/isomerase family	4-288	2,9E-18	
								Polysaccharide biosynthesis protein	3-183	6,1E-12	
								NAD binding domain	41-229	0,000001	
**1968**	**PapC**	248	**Inner membrane**	2,381	10	1	56-80	Bacterial sugar transferase	54-243	2,3E-61	Undecaprenyl-phosphate galactose phosphotransferase/Glycosyltransferase

**1967**	**PapD**	214	Outer membrane/**inner membrane**	1,864	9,97	0	-	Cellulose biosynthesis protein BcsQ	27-192	7,1E-09	Protein EpsD / CpsD/CapB family tyrosine-kinase protein
**1966**	**PapE**	340	**Outer membrane**	2,08	-	0	-	Polysaccharide biosynthesis/export protein	63-144	5,7E-13	Putative polysaccharide biosynthesis/export familiy protein
								Polysaccharide biosynthesis/export protein	135-216	9,4E-09	
								SLBB domain	223-273	9,1E-10	
**1965**	**PapF**	530	**Inner membrane**	2,65	9,82	2	21-40, 460-479	Chain length determinant protein (Wzz) domain	7-101	0,000000014	Lipopolysaccharide biosynthesis protein/Chain length determinant
**1964**	**PapG**	367	**Cytoplasmic**	2,736	9,26	0	-	Glycosyl transferases group 1	177-340	7,1E-35	Group 4 glycosyl transferase
								Glycosyl transferases group 1	186-326	2,1E-30	
								Glycosyltransferase Family 4	27-173	1,6E-13	
								Glycosyl transferase 4-like domain	33-170	3,8E-127	
								Glycosyl transferases group 1	263-357	0,00000001	
**1963**	**PapH**	456	**Inner membrane**	4,932	10	11	7-41, 64-86, 98-116, 126-143, 150-172, 187-204, 209-227, 233-249, 261-283, 369-391, 404-426	O-Antigen ligase (Wzy-C)	217-378	3,3E-16	Hypothetical protein/Polymerase/O-Antigen ligase family protein
**1962**	**PapI**	319	**Inner membrane/** Cytoplasmic	2,837	8,96	1	254-276	Glycosyl transferase family 2	5-162	1,1E-21	Group 2 glycosyl transferase family protein
								Glycosyltransferase like family 2	2-216	1,1E-12	
								Glycosyl transferase family group 2	82-305	0,000000001	

								Glycosyl transferase family 21	69-214	0,0000011	
**1961**	**PapJ**	378	**Inner membrane**	4,283	10	4	14-33, 288-307, 313-333, 345-364	Glycosyl transferase family 2	45-193	1,5E-26	Group 2 glycosyl transferase family protein
								Glycosyltransferase like family 2	41-259	2,5E-19	
								Glycosyltransferase like family 2	43-146	6,4E-11	
								Glycosyl transferase family group 2	124-358	0,000000026	
								Glycosyl transferase family 21	96-257	0,00000062	
**1960**	**PapK**	334	**Cytoplasmic**	4,179	9,97	0	-	Polysaccharide deacetylase	85-291	2,7E-25	Polysaccharide deacetylase
**1959**	**PapL**	434	**Inner membrane**	4,569	10	9	113-135, 147-169, 174-196, 236-258, 260-277, 296-318, 325-344, 354-376, 381-403	Polysaccharide biosynthesis protein	30-326	3E-21	Polysaccharide biosynthesis protein membrane protein/Oligosaccharide flippase family protein
								Polysaccharide biosynthesis protein	7-277	3,6E-17	
								Polysaccharide biosynthesis C-terminal domain	327-420	1,1E-12	
**1958**	**PapM**	658	**Periplasmic**	3,295	-	1	7-25	-	-	-	Hypothetical protein
**1957**	**PapN**	229	**Cytoplasmic**	2,977	8,96	0	-	-	-	-	Hypothetical protein
**1956**	**PapO**	391	**Cytoplasmic**	2,184	8,96	0	-	Acetyltransferase (GNAT) domain	171-315	9,4E-22	Cellulose biosynthesis protein/GNAT family N-acetyltransferase
**1955**	**PapP**	260	**Cytoplasmic**	2,553	8,96	0	-	YdjC-like protein	1-233	3,5E-43	Cellobiose phosphotransferase system YdjC-like protein
