# Supplementary material for: In Silico Characterization and Phylogenetic Distribution of Extracellular Matrix Components in the Model Rhizobacteria Pseudomonas fluorescens F113 and Other Pseudomonads
Source: Microorganisms. 2020 Nov 6;8(11):1740. doi: 10.3390/microorganisms8111740 (PMC7716237; doi:10.3390/microorganisms8111740)
Supplement: Supplementary file 1 [file microorganisms-08-01740-s001.zip › Supplementary_Table_6.pdf]

Supplementary Table 6. Pseudomonads containing the fimbrial low-molecular-weight protein (*flp*)/tight adherence *(tad)* and/or *Pseudomonas* acidic polysaccharide (*pap)* biosynthetic clusters: the rhizosphere lifestyle of the different taxa is highlighted as PGPR (B) or pathogenic (P) behavior according to previous reports in the literature.

Species	Subps/Strain/ Pv/Isolate	Flp/Tad Type A	Flp/Tad Type B	Pap	Plant-bacteria relationship	Reference
Pseudomonas aeruginosa	PSE305	+	-	-	/	/
Pseudomonas aeruginosa	CCBH4851	+	-	-	/	/
Pseudomonas aeruginosa	F22031	+	-	-	/	/
Pseudomonas aeruginosa	NCGM1984	+	-	-	/	/
Pseudomonas aeruginosa	NCGM1900	+	-	-	/	/
Pseudomonas aeruginosa	Carb01 63	+	-	-	/	/
Pseudomonas aeruginosa	S04 90	+	-	-	/	/
Pseudomonas aeruginosa	F9676	+	-	-	/	/
Pseudomonas aeruginosa	PA1RG	+	-	-	/	/
Pseudomonas aeruginosa	VA-134	+	-	-	/	/
Pseudomonas aeruginosa	38089 59	+	-	-	/	/
Pseudomonas aeruginosa	S86968	+	-	-	/	/
Pseudomonas aeruginosa	T38079	+	-	-	/	/
Pseudomonas aeruginosa	T52373	+	-	-	/	/
Pseudomonas aeruginosa	T63266	+	-	-	/	/
Pseudomonas aeruginosa	W16407	+	-	-	/	/
Pseudomonas aeruginosa	W36662	+	-	-	/	/
Pseudomonas aeruginosa	W60856	+	-	-	/	/
Pseudomonas aeruginosa	F23197	+	-	-	/	/
Pseudomonas aeruginosa	F30658	+	-	-	/	/
Pseudomonas aeruginosa	H5708	+	-	-	/	/
Pseudomonas aeruginosa	H27930	+	-	-	/	/
Pseudomonas aeruginosa	H47921	+	-	-	/	/
Pseudomonas aeruginosa	M1608	+	-	-	/	/
Pseudomonas aeruginosa	M37351	+	-	-	/	/
Pseudomonas aeruginosa	USDA-ARS-USMARC-41639	+	-	-	/	/
Pseudomonas aeruginosa	X78812	+	-	-	/	/
Pseudomonas aeruginosa	F9670	+	-	-	/	/
Pseudomonas aeruginosa	NCGM257	+	-	-	/	/
Pseudomonas aeruginosa	8380	+	-	-	/	/
Pseudomonas aeruginosa	IOMTU 133	+	-	-	/	/
Pseudomonas aeruginosa	F63912	+	-	-	/	/
Pseudomonas aeruginosa	N17-1	+	-	-	/	/
Pseudomonas aeruginosa	BAMCPA07-48	+	-	-	/	/
Pseudomonas aeruginosa	PA121617	+	-	-	/	/
Pseudomonas aeruginosa	ATCC 27853	+	-	-	/	/
Pseudomonas aeruginosa	PA D1	+	-	-	/	/

Pseudomonas aeruginosa	PA D2	+	-	-	/	/
Pseudomonas aeruginosa	PA D9	+	-	-	/	/
Pseudomonas aeruginosa	PA D16	+	-	-	/	/
Pseudomonas aeruginosa	PA D22	+	-	-	/	/
Pseudomonas aeruginosa	PA D25	+	-	-	/	/
Pseudomonas aeruginosa	DN1	+	-	-	/	/
Pseudomonas aeruginosa	PA D5	+	-	-	/	/
Pseudomonas aeruginosa	PA D21	+	-	-	/	/
Pseudomonas aeruginosa	ATCC 15692	+	-	-	/	/
Pseudomonas aeruginosa	PA1088	+	-	-	/	/
Pseudomonas aeruginosa	PA8281	+	-	-	/	/
Pseudomonas aeruginosa	PA11803	+	-	-	/	/
Pseudomonas aeruginosa	PA7790	+	-	-	/	/
Pseudomonas aeruginosa	B10W	+	-	-	/	/
Pseudomonas aeruginosa	Paer4 119	+	-	-	/	/
Pseudomonas aeruginosa	SCVFeb	+	-	-	/	/
Pseudomonas aeruginosa	SCVJan	+	-	-	/	/
Pseudomonas aeruginosa	NHmuc	+	-	-	/	/
Pseudomonas aeruginosa	PA 154197	+	-	-	/	/
Pseudomonas aeruginosa	RIVM-EMC2982	+	-	-	/	/
Pseudomonas aeruginosa	E6130952	+	-	-	/	/
Pseudomonas aeruginosa	PASGNDM699	+	-	-	/	/
Pseudomonas aeruginosa	PASGNDM345	+	-	-	/	/
Pseudomonas aeruginosa	M8A1	+	-	-	/	/
Pseudomonas aeruginosa	M8A4	+	-	-	/	/
Pseudomonas aeruginosa	M28A1	+	-	-	/	/
Pseudomonas aeruginosa	Pb18	+	-	-	/	/
Pseudomonas aeruginosa	Pa124	+	-	-	/	/
Pseudomonas aeruginosa	Pa58	+	-	-	/	/
Pseudomonas aeruginosa	Pa84	+	-	-	/	/
Pseudomonas aeruginosa	Pa127	+	-	-	/	/
Pseudomonas aeruginosa	Pa1242	+	-	-	/	/
Pseudomonas aeruginosa	PA83	+	-	-	/	/
Pseudomonas aeruginosa	L10	+	-	-	/	/
Pseudomonas aeruginosa	Ocean-1155	+	-	-	/	/
Pseudomonas aeruginosa	Ocean-1175	+	-	-	/	/
Pseudomonas aeruginosa	PPF-1	+	-	-	/	/
Pseudomonas aeruginosa	97	+	-	-	/	/
Pseudomonas aeruginosa	PA 150577	+	-	-	/	/
Pseudomonas aeruginosa	12939	+	-	-	/	/
Pseudomonas aeruginosa	PB369	+	-	-	/	/
Pseudomonas aeruginosa	PB368	+	-	-	/	/
Pseudomonas aeruginosa	PB353	+	-	-	/	/
Pseudomonas aeruginosa	PB354	+	-	-	/	/

Pseudomonas aeruginosa	PB350	+	-	-	/	/
Pseudomonas aeruginosa	PB367	+	-	-	/	/
Pseudomonas aeruginosa	F5677	+	-	-	/	/
Pseudomonas aeruginosa	AR 360	+	-	-	/	/
Pseudomonas aeruginosa	AR 354	+	-	-	/	/
Pseudomonas aeruginosa	AR 353	+	-	-	/	/
Pseudomonas aeruginosa	AR 230	+	-	-	/	/
Pseudomonas aeruginosa	AR 356	+	-	-	/	/
Pseudomonas aeruginosa	AR 357	+	-	-	/	/
Pseudomonas aeruginosa	AR 95	+	-	-	/	/
Pseudomonas aeruginosa	CR1	+	-	-	/	/
Pseudomonas aeruginosa	MRSN12280	+	-	-	/	/
Pseudomonas aeruginosa	WCHPA075019	+	-	-	/	/
Pseudomonas aeruginosa	JB2	+	-	-	/	/
Pseudomonas aeruginosa	AR444	+	-	-	/	/
Pseudomonas aeruginosa	AR441	+	-	-	/	/
Pseudomonas aeruginosa	AR445	+	-	-	/	/
Pseudomonas aeruginosa	AR442	+	-	-	/	/
Pseudomonas aeruginosa	AR439	+	-	-	/	/
Pseudomonas aeruginosa	AR 440	+	-	-	/	/
Pseudomonas aeruginosa	AR 443	+	-	-	/	/
Pseudomonas aeruginosa	AR 446	+	-	-	/	/
Pseudomonas aeruginosa	CCUG 70744	+	-	-	/	/
Pseudomonas aeruginosa	AR 110	+	-	-	/	/
Pseudomonas aeruginosa	K34-7	+	-	-	/	/
Pseudomonas aeruginosa	AR 460	+	-	-	/	/
Pseudomonas aeruginosa	AR 455	+	-	-	/	/
Pseudomonas aeruginosa	AR 458	+	-	-	/	/
Pseudomonas aeruginosa	PA34	+	-	-	/	/
Pseudomonas aeruginosa	Y89	+	-	-	/	/
Pseudomonas aeruginosa	Y82	+	-	-	/	/
Pseudomonas aeruginosa	Y31	+	-	-	/	/
Pseudomonas aeruginosa	Y71	+	-	-	/	/
Pseudomonas aeruginosa	PABL048	+	-	-	/	/
Pseudomonas aeruginosa	PABL012	+	-	-	/	/
Pseudomonas aeruginosa	PABL017	+	-	-	/	/
Pseudomonas aeruginosa	24Pae112	+	-	-	/	/
Pseudomonas aeruginosa	N15-01092	+	-	-	/	/
Pseudomonas aeruginosa	AR 111	+	-	-	/	/
Pseudomonas aeruginosa	BA7823	+	-	-	/	/
Pseudomonas aeruginosa	268	+	-	-	/	/
Pseudomonas aeruginosa	6762	+	-	-	/	/
Pseudomonas aeruginosa	SP4528	+	-	-	/	/
Pseudomonas aeruginosa	BH9	+	-	-	/	/
Pseudomonas aeruginosa	H26027	+	-	-	/	/
Pseudomonas aeruginosa	H25883	+	-	-	/	/

Pseudomonas aeruginosa	H26023	+	-	-	/	/
Pseudomonas aeruginosa	FDAARGOS 532	+	-	-	/	/
Pseudomonas aeruginosa	FDAARGOS 571	+	-	-	/	/
Pseudomonas aeruginosa	FDAARGOS 501	+	-	-	/	/
Pseudomonas aeruginosa	FDAARGOS 505	+	-	-	/	/
Pseudomonas aeruginosa	FDAARGOS 570	+	-	-	/	/
Pseudomonas aeruginosa	IMP-13	+	-	-	/	/
Pseudomonas aeruginosa	SP4371	+	-	-	/	/
Pseudomonas aeruginosa	SP2230	+	-	-	/	/
Pseudomonas aeruginosa	B14130	+	-	-	/	/
Pseudomonas aeruginosa	B17932	+	-	-	/	/
Pseudomonas aeruginosa	GIMC5015 PAKB6	+	-	-	/	/
Pseudomonas aeruginosa	SP4527	+	-	-	/	/
Pseudomonas aeruginosa	LW	+	-	-	/	/
Pseudomonas aeruginosa	PAO1161	+	-	-	/	/
Pseudomonas aeruginosa	1334 14	+	-	-	/	/
Pseudomonas aeruginosa	E80	+	-	-	/	/
Pseudomonas aeruginosa	PA-VAP-3	+	-	-	/	/
Pseudomonas aeruginosa	PA-VAP-1	+	-	-	/	/
Pseudomonas aeruginosa	PA-VAP-2	+	-	-	/	/
Pseudomonas aeruginosa	PA-VAP-4	+	-	-	/	/
Pseudomonas aeruginosa	AES1M	+	-	-	/	/
Pseudomonas aeruginosa	AES1R	+	-	-	/	/
Pseudomonas aeruginosa	AJ D 2	+	-	-	/	/
Pseudomonas aeruginosa	PRD-10	+	-	-	/	/
Pseudomonas aeruginosa	PA298	+	-	-	/	/
Pseudomonas aeruginosa	FDAARGOS 767	+	-	-	/	/
Pseudomonas aeruginosa	FDAARGOS 610	+	-	-	/	/
Pseudomonas aeruginosa	AZPAE15042	+	-	-	/	/
Pseudomonas aeruginosa	60503	+	-	-	/	/
Pseudomonas aeruginosa	A681	+	-	-	/	/
Pseudomonas aeruginosa	519119	+	-	-	/	/
Pseudomonas aeruginosa	243931	+	-	-	/	/
Pseudomonas aeruginosa	SCAID WND2-2019	+	-	-	/	/
Pseudomonas aeruginosa	SCAID WND1-2019	+	-	-	/	/
Pseudomonas aeruginosa	C79	+	-	-	/	/
Pseudomonas aeruginosa	IMP67	+	-	-	/	/
Pseudomonas aeruginosa	IMP68	+	-	-	/	/
Pseudomonas aeruginosa	IMP66	+	-	-	/	/
Pseudomonas aeruginosa	CCUG 51971	+	-	-	/	/

Pseudomonas aeruginosa	HOU1	+	-	-	/	/
Pseudomonas aeruginosa	GIMC5001 PAT-23	+	-	-	/	/
Pseudomonas aeruginosa	GIMC5002 PAT-169	+	-	-	/	/
Pseudomonas aeruginosa	E90	+	-	-	/	/
Pseudomonas aeruginosa	PA59	+	-	-	/	/
Pseudomonas aeruginosa	CFSAN084950	+	-	-	/	/
Pseudomonas aeruginosa	AG1	+	-	-	/	/
Pseudomonas aeruginosa	ST773	+	-	-	/	/
Pseudomonas aeruginosa	1811-13R031	+	-	-	/	/
Pseudomonas aeruginosa	1811-18R001	+	-	-	/	/
Pseudomonas aeruginosa	T2436	+	-	-	/	/
Pseudomonas aeruginosa	T2101	+	-	-	/	/
Pseudomonas aeruginosa	delta6 2	+	-	-	/	/
Pseudomonas aeruginosa	delta6 3	+	-	-	/	/
Pseudomonas aeruginosa	delta6 4	+	-	-	/	/
Pseudomonas aeruginosa	delta6 5	+	-	-	/	/
Pseudomonas aeruginosa	delta6 6	+	-	-	/	/
Pseudomonas aeruginosa	delta10	+	-	-	/	/
Pseudomonas aeruginosa	Cas9 1	+	-	-	/	/
Pseudomonas aeruginosa	Cas9 2	+	-	-	/	/
Pseudomonas aeruginosa	Environ 1	+	-	-	/	/
Pseudomonas aeruginosa	Environ 2	+	-	-	/	/
Pseudomonas aeruginosa	YB01	+	-	-	/	/
Pseudomonas aeruginosa	INP-43	+	-	-	/	/
Pseudomonas aeruginosa	RD1-3	+	-	-	/	/
Pseudomonas aeruginosa	PAO1 Orsay	+	-	-	/	/
Pseudomonas aeruginosa	PA14Or reads	+	-	-	/	/
Pseudomonas aeruginosa	PcyII-10	+	-	-	/	/
Pseudomonas aeruginosa	RW109	+	-	-	/	/
Pseudomonas aeruginosa	1	+	-	-	/	/
Pseudomonas aeruginosa	paerg002	+	-	-	/	/
Pseudomonas aeruginosa	paerg004	+	-	-	/	/
Pseudomonas aeruginosa	paerg003	+	-	-	/	/
Pseudomonas aeruginosa	paerg005	+	-	-	/	/
Pseudomonas aeruginosa	paerg009	+	-	-	/	/
Pseudomonas aeruginosa	paerg010	+	-	-	/	/
Pseudomonas aeruginosa	paerg011	+	-	-	/	/
Pseudomonas aeruginosa	paerg012	+	-	-	/	/
Pseudomonas aeruginosa	NCTC11445	+	-	-	/	/
Pseudomonas aeruginosa	NCTC12903	+	-	-	/	/
Pseudomonas aeruginosa	NCTC13715	+	-	-	/	/
Pseudomonas aeruginosa	NCTC10728	+	-	-	/	/
Pseudomonas aeruginosa	NCTC13359	+	-	-	/	/
Pseudomonas aeruginosa	NCTC13620	+	-	-	/	/
Pseudomonas aeruginosa	PcyII-29	+	-	-	/	/

Pseudomonas aeruginosa	PcyII-40	+	-	-	/	/
Pseudomonas aeruginosa	C7-25	+	-	-	/	/
Pseudomonas aeruginosa	19BR	+	-	-	/	/
Pseudomonas aeruginosa	213BR	+	-	-	/	/
Pseudomonas aeruginosa	39016	+	-	-	/	/
Pseudomonas aeruginosa	B136-33	+	-	-	/	/
Pseudomonas aeruginosa	c7447m	+	-	-	/	/
Pseudomonas aeruginosa	C-NN2	+	-	-	/	/
Pseudomonas aeruginosa	DHS01	+	-	-	/	/
Pseudomonas aeruginosa	DK1 substr NH57388A	+	-	-	/	/
Pseudomonas aeruginosa	DK2	+	-	-	/	/
Pseudomonas aeruginosa	DSM 50071 NBRC 12689	+	-	-	/	/
Pseudomonas aeruginosa	LES400	+	-	-	/	/
Pseudomonas aeruginosa	LES431	+	-	-	/	/
Pseudomonas aeruginosa	LESB58	+	-	-	/	/
Pseudomonas aeruginosa	LESB65	+	-	-	/	/
Pseudomonas aeruginosa	LESlike1	+	-	-	/	/
Pseudomonas aeruginosa	LESlike4	+	-	-	/	/
Pseudomonas aeruginosa	LESlike5	+	-	-	/	/
Pseudomonas aeruginosa	LESlike7	+	-	-	/	/
Pseudomonas aeruginosa	M18	+	-	-	/	/
Pseudomonas aeruginosa	MTB-1	+	-	-	/	/
Pseudomonas aeruginosa	NCGM2 S1	+	-	-	/	/
Pseudomonas aeruginosa	PA1	+	-	-	/	/
Pseudomonas aeruginosa	PA1R	+	-	-	/	/
Pseudomonas aeruginosa	PA7	+	-	-	/	/
Pseudomonas aeruginosa	PA96	+	-	-	/	/
Pseudomonas aeruginosa	PACS2	+	-	-	/	/
Pseudomonas aeruginosa	PAK	+	-	-	/	/
Pseudomonas aeruginosa	PAO1	+	-	-	/	/
Pseudomonas aeruginosa	PAO1-VE13	+	-	-	/	/
Pseudomonas aeruginosa	PAO1-VE2	+	-	-	/	/
Pseudomonas aeruginosa	PAO581	+	-	-	/	/
Pseudomonas aeruginosa	RP73	+	-	-	/	/
Pseudomonas aeruginosa	SCV20265	+	-	-	/	/
Pseudomonas aeruginosa	SJTD-1	+	-	-	/	/
Pseudomonas aeruginosa	UCBPP-PA14	+	-	-	/	/
Pseudomonas aeruginosa	VRFPA04	+	-	-	/	/
Pseudomonas aeruginosa	YL84	+	-	-	/	/
Pseudomonas amygdali	pv *lachrymans* NM002	+	-	-	P	[1]
Pseudomonas amygdali	pv *lachrymans* str M301315	+	-	-	/	/
Pseudomonas amygdali	pv *morsprunorum* R15244	+	-	-	/	/

Pseudomonas amygdali	pv *tabaci* str ATCC 11528	+	-	-	P	[2]
Pseudomonas antarctica	PAMC 27494	+	-	-	/	/
Pseudomonas antarctica	LMG 22709	+	-	-	/	/
Pseudomonas arsenicoxydans	CECT 7543	+	-	+	/	/
Pseudomonas asplenii	ATCC 23835	+	-	-	P	[3]
Pseudomonas azotoformans	S4	+	-	-	B	[4]
Pseudomonas azotoformans	F77	+	-	-	/	/
Pseudomonas azotoformans	P45A	+	-	-	/	/
Pseudomonas azotoformans	LMG 21611	+	-	-	/	/
Pseudomonas balearica	DSM 6083 SP1402	-	+	-	/	/
Pseudomonas brassicacearum	DF41	-	+	-	B	[5]
Pseudomonas brassicacearum	LBUM300	-	+	+	B	[6]
Pseudomonas brassicacearum	L13-6-12	-	+	-	B	[7]
Pseudomonas brassicacearum	3Re2-7	-	+	+	B	[8]
Pseudomonas brassicacearum	S-1	-	+	+	/	/
Pseudomonas brassicacearum	LMG 21623	-	+	+	/	/
Pseudomonas brassicacearum	subsp *brassicacearum* NFM421	-	+	+	B	[9]
Pseudomonas brenneri	DSM 15294	+	-	-	/	/
Pseudomonas cedrina	LMG 23661	+	-	-	/	/
Pseudomonas cerasi	Sour cherry Prunus cerasus symptomatic leaf	+	-	-	P	[10]
Pseudomonas cerasi	PL963	+	-	-	P	[10]
Pseudomonas chlororaphis	PA23	+	+	+	B	[11]
Pseudomonas chlororaphis	PCL1606	+	+	-	B	[12]
Pseudomonas chlororaphis	UFB2	-	+	+	B	[13]
Pseudomonas chlororaphis	189	+	+	-	B	[14]
Pseudomonas chlororaphis	Lzh-T5	+	+	+	B	[15]
Pseudomonas chlororaphis	TAMOak81	+	+	-	/	/
Pseudomonas chlororaphis	ATCC 17415	+	+	-	/	/
Pseudomonas chlororaphis	Pb-St2	+	+	+	B	[16]
Pseudomonas chlororaphis	B25	+	+	-	/	/
Pseudomonas chlororaphis	R47	+	+	+	B	[17]
Pseudomonas chlororaphis	ATCC 13985	+	+	+	/	/
Pseudomonas chlororaphis	LMG 21630	+	+	+	/	/
Pseudomonas chlororaphis	DSM 21509	+	+	+	/	/
Pseudomonas chlororaphis	NCTC7357	+	+	+	/	/
Pseudomonas chlororaphis	O6	+	+	+	B	[18]
Pseudomonas chlororaphis	subsp *aurantiaca* JD37	+	+	+	B	[19]
Pseudomonas chlororaphis	subsp *aurantiaca* StFRB508	+	+	+	B	[20]

Pseudomonas chlororaphis	subsp *aurantiaca* M12	+	+	+	/	/
Pseudomonas chlororaphis	subsp *aurantiaca* 449	+	+	+	/	/
Pseudomonas chlororaphis	subsp *aurantiaca* CW2	+	+	+	/	/
Pseudomonas chlororaphis	subsp *aurantiaca* M71	+	+	+	B	[21]
Pseudomonas chlororaphis	subsp *aurantiaca* K27	+	-	+	/	/
Pseudomonas chlororaphis	subsp *aurantiaca* PCM 2210	+	+	+	/	/
Pseudomonas chlororaphis	subsp *aurantiaca* Q16	+	+	+	B	[22]
Pseudomonas chlororaphis	subsp *aurantiaca* 464	+	+	+	/	/
Pseudomonas chlororaphis	subsp *aurantiaca* DSM 19603	+	+	+	/	/
Pseudomonas chlororaphis	subsp *aurantiaca* ARS 38	+	+	+	B	[23]
Pseudomonas chlororaphis	subsp *aurantiaca* zm-1	+	+	+	/	/
Pseudomonas chlororaphis	subsp *aureofaciens* P2	-	+	+	/	/
Pseudomonas chlororaphis	subsp *aureofaciens* C50	+	+	+	/	/
Pseudomonas chlororaphis	subsp *aureofaciens* 66	+	+	+	/	/
Pseudomonas chlororaphis	subsp *aureofaciens* ChPhzS23	-	-	+	/	/
Pseudomonas chlororaphis	subsp *aureofaciens* ChPhzS24	+	+	+	/	/
Pseudomonas chlororaphis	subsp *aureofaciens* ChPhzTR36	+	+	+	/	/
Pseudomonas chlororaphis	subsp *aureofaciens* DSM 6698	+	+	+	/	/
Pseudomonas chlororaphis	subsp *aureofaciens* ChPhzTR39	-	+	+	/	/
Pseudomonas chlororaphis	subsp *aureofaciens* ChPhzTR18	+	+	+	/	/
Pseudomonas chlororaphis	subsp *aureofaciens* ChPhzTR38	+	+	+	/	/

Pseudomonas chlororaphis	subsp *aureofaciens* 30-84	+	+	-	B	[24]
Pseudomonas chlororaphis	subsp *chlororaphis* DSM 50083	+	+	-	/	/
Pseudomonas chlororaphis	subsp *piscium* ZJU60	+	+	+	/	/
Pseudomonas chlororaphis	subsp *piscium* DSM 21509	+	+	+	/	/
Pseudomonas chlororaphis	subsp *piscium* ATCC 17809	+	+	+	/	/
Pseudomonas chlororaphis	subsp *piscium* ATCC 17411	+	+	+	/	/
Pseudomonas chlororaphis	subsp *piscium* SLPH10	+	+	+	/	/
Pseudomonas chlororaphis	subsp *piscium* DTR133	+	+	+	/	/
Pseudomonas chlororaphis	subsp *piscium* PCL1391	+	+	+	/	/
Pseudomonas chlororaphis	subsp *piscium* PCL1607	+	+	+	/	/
Pseudomonas chlororaphis	subsp *piscium* ChPhzS135	+	+	+	/	/
Pseudomonas chlororaphis	subsp *piscium* ChPhzS140	+	+	+	/	/
Pseudomonas chlororaphis	subsp *piscium* ChPhzTR44	+	+	+	/	/
Pseudomonas chlororaphis	subsp *piscium* ToZa7	+	+	+	/	/
Pseudomonas cichorii	JBC1	+	-	-	P	[25]
Pseudomonas coronafaciens	pv oryzae str 1 6	+	-	-	P	[26]
Pseudomonas corrugata	RM1-1-4	-	+	+	B	[27]
Pseudomonas corrugata	LMG 2172	-	+	+	P	[28]
Pseudomonas cremoricolorata	ND07	-	-	+	/	/
Pseudomonas entomophila	2014	+	-	-	/	/
Pseudomonas entomophila	1257	+	-	-	/	/
Pseudomonas entomophila	L48 L48	+	-	-	/	/
Pseudomonas extremorientalis	LMG 19695	+	-	-	/	/
Pseudomonas fluorescens	UK4	+	-	-	/	/
Pseudomonas fluorescens	FW300-N2C3	-	+	+	/	/
Pseudomonas fluorescens	FW300-N2E2	-	+	+	/	/
Pseudomonas fluorescens	Pt14	+	-	-	/	/
Pseudomonas fluorescens	2P24	-	-	+	B	[29]
Pseudomonas fluorescens	FR1	-	+	+	B	[30]
Pseudomonas fluorescens	MS82	+	-	+	B	[31]
Pseudomonas fluorescens	NEP1	+	-	+	/	/
Pseudomonas fluorescens	Pf275	-	+	+	B	[32]
Pseudomonas fluorescens	SIK W1	+	-	-	/	/
Pseudomonas fluorescens	LBUM677	+	-	+	B	[33]
Pseudomonas fluorescens	ATCC 13525	+	-	-	/	/

Pseudomonas fluorescens	NCTC10038	+	-	-	/	/
Pseudomonas fluorescens	NCTC10783	+	-	-	/	/
Pseudomonas fluorescens	NCTC9428	+	-	+	/	/
Pseudomonas fluorescens	A506	+	-	-	/	/
Pseudomonas fluorescens	F113	-	+	+	B	[34]
Pseudomonas fluorescens	NCIMB 11764	+	-	-	/	/
Pseudomonas fluorescens	Pf0-1	+	-	+	B	[35]
Pseudomonas fluorescens	Q2-87	-	+	+	B	[36]
Pseudomonas fluorescens	Q8r1-96	-	+	+	B	[37]
Pseudomonas fluorescens	R124	+	-	+	/	/
Pseudomonas fluorescens	SBW25	+	-	-	B	[38]
Pseudomonas fragi	P121	+	-	-	/	/
Pseudomonas fragi	NMC25	+	+	-	/	/
Pseudomonas fragi	NRRL B-727	+	+	-	/	/
Pseudomonas frederiksbergensis	AS1	+	+	-	/	/
Pseudomonas frederiksbergensis	KNU-15	+	+	+	/	/
Pseudomonas fuscovaginae	LMG 2158	+	-	-	P	[39]
Pseudomonas granadensis	LMG 27940	+	-	+	/	/
Pseudomonas koreensis	D26	+	-	+	/	/
Pseudomonas koreensis	CRS05-R5	+	-	+	B	[40]
Pseudomonas koreensis	LMG 21318	+	-	+	/	/
Pseudomonas kribbensis	46-2	+	-	+	/	/
Pseudomonas lactis	SS101	+	-	-	/	/
Pseudomonas libanensis	DMSP-1	+	-	-	/	/
Pseudomonas libanensis	DSM 17149	+	-	-	/	/
Pseudomonas lini	DSM 16768	+	+	-	/	/
Pseudomonas lundensis	AU1044	+	-	-	/	/
Pseudomonas lurida	L228	+	-	-	/	/
Pseudomonas lurida	MYb11	+	-	-	/	/
Pseudomonas mandelii	LMG 21607	+	-	+	/	/
Pseudomonas mandelii	JR-1	+	-	+	/	/
Pseudomonas mediterranea	DSM 16733	-	+	+	/	/
Pseudomonas monteilii	USDA-ARS-USMARC-56711	-	-	+	/	/
Pseudomonas moraviensis	LMG 24280	+	-	+	/	/
Pseudomonas moraviensis	R28-S	+	-	+	/	/
Pseudomonas mosselii	BS011	+	-	+	B	[41]
Pseudomonas mosselii	PtA1	+	-	+	/	/
Pseudomonas orientalis	F9	+	-	-	B	[42]
Pseudomonas orientalis	L1-3-08	+	-	-	/	/
Pseudomonas orientalis	R2-66-08W	+	-	-	B	[43]
Pseudomonas orientalis	R4-35-08	+	-	-	/	/
Pseudomonas orientalis	8B	+	-	-	/	/
Pseudomonas orientalis	LMG 23660	+	-	-	/	/
Pseudomonas palleroniana	MAB3	+	-	-	B	[44]
Pseudomonas parafulva	CRS01-1	+	-	+	B	[45]

Pseudomonas parafulva	JBCS1880	+	-	+	B	[46]
Pseudomonas prosekii	LMG 26867	-	-	+	/	/
Pseudomonas protegens	H78	+	-	+	B	[47]
Pseudomonas protegens	FDAARGOS 307	+	-	+	/	/
Pseudomonas protegens	FD6	+	-	+	B	[48]
Pseudomonas protegens	pf5	+	-	+	/	/
Pseudomonas protegens	pf5-k3	+	-	-	/	/
Pseudomonas protegens	pf5-k2	+	-	+	/	/
Pseudomonas protegens	SN15-2	+	-	+	B	[49]
Pseudomonas protegens	Cab57	+	-	+	B	[50]
Pseudomonas protegens	CHA0	+	-	+	B	[51]
Pseudomonas protegens	Pf-5	+	-	+	B	[52]
Pseudomonas psychrophila	LMG 24276	+	+	-	/	/
Pseudomonas putida	1A00316	+	-	+	B	[53]
Pseudomonas putida	PC2	+	-	+	/	/
Pseudomonas putida	DRA525	-	+	-	/	/
Pseudomonas putida	KF715	+	-	-	/	/
Pseudomonas putida	IEC33019	+	-	-	/	/
Pseudomonas putida	JBC17	+	-	-	B	[54]
Pseudomonas putida	NX-1	+	-	+	/	/
Pseudomonas putida	1290	-	-	+	/	/
Pseudomonas putida	B1	+	-	-	/	/
Pseudomonas putida	JYR-1	+	-	-	/	/
Pseudomonas putida	H8234	+	-	-	P	[55]
Pseudomonas putida	HB3267 PC9	-	+	-	/	/
Pseudomonas putida	NBRC 14164	+	-	-	/	/
Pseudomonas putida	W619	+	-	-	B	[56]
Pseudomonas reinekei	DSM 18361	+	-	+	/	/
Pseudomonas rhodesiae	LMG 17764	+	-	-	/	/
Pseudomonas saudiphocaensis	20 BN	-	-	+	/	/
Pseudomonas savastanoi	pv *phaseolicola* 1448A BAA-978	+	-	-	/	/
Pseudomonas savastanoi	pv *savastanoi* NCPPB 3335	+	-	-	P	[57]
Pseudomonas simiae	WCS417	+	-	-	B	[58]
Pseudomonas simiae	PCL1751	+	-	-	B	[59]
Pseudomonas simiae	PICF7	+	-	-	/	/
Pseudomonas soli	SJ10	+	-	+	/	/
Pseudomonas stutzeri	28a24	-	-	+	/	/
Pseudomonas stutzeri	DW2-1	-	+	-	/	/
Pseudomonas stutzeri	PheN2	-	+	-	/	/
Pseudomonas stutzeri	XL272	-	+	-	/	/
Pseudomonas stutzeri	NCTC10475	-	+	-	/	/
Pseudomonas stutzeri	RCH2	-	+	-	/	/
Pseudomonas synxantha	LBUM223	+	-	-	B	[60]
Pseudomonas synxantha	KENGFT3	+	-	-	/	/

Pseudomonas synxantha	30B	+	-	-	/	/
Pseudomonas synxantha	28887	+	-	-	/	/
Pseudomonas synxantha	R2-4-08W	+	-	-	/	/
Pseudomonas synxantha	R6-28-08	+	-	-	/	/
Pseudomonas synxantha	R2-54-08W	+	-	-	/	/
Pseudomonas synxantha	LMG 2190	+	-	-	/	/
Pseudomonas synxantha	NCTC10696	+	-	-	/	/
Pseudomonas synxantha	BG33R	+	-	-	/	/
Pseudomonas syringae	inb918	+	-	+	/	/
Pseudomonas syringae	Ps25	+	-	-	/	/
Pseudomonas syringae	31R1	+	-	-	/	/
Pseudomonas syringae	CFBP3840	+	-	-	/	/
Pseudomonas syringae	CC1557	+	-	-	P	[61]
Pseudomonas syringae	group genomosp 3 CFBP6411	+	-	-	/	/
Pseudomonas syringae	pv *actinidiae* ICMP 20586	+	-	-	/	/
Pseudomonas syringae	pv *actinidiae* NZ-47	+	-	-	/	/
Pseudomonas syringae	pv *actinidiae* CRAFRU 12.29	+	-	-	P	[62]
Pseudomonas syringae	pv *actinidiae* CRAFRU 14.8	+	-	-	/	/
Pseudomonas syringae	pv *actinidiae* P155	+	-	-	/	/
Pseudomonas syringae	pv *actinidiae* ICMP18708	+	-	-	P	[63]
Pseudomonas syringae	pv *actinidiae* ICMP18884	+	-	-	P	[64]
Pseudomonas syringae	pv *actinidiae* ICMP9617	+	-	-	P	[64]
Pseudomonas syringae	pv *actinidiae* ICMP9853	+	-	-	P	[65]
Pseudomonas syringae	pv *actinidiae* str Shaanxi M228	+	-	-	P	[66]
Pseudomonas syringae	pv *atrofaciens* LMG5095	+	-	-	/	/
Pseudomonas syringae	pv *avii* CFBP3846	+	-	-	P	[67]
Pseudomonas syringae	pv *cerasicola* CFBP6109	+	-	-	P	[68]
Pseudomonas syringae	pv *lapsa* ATCC 10859	+	-	-	P	[69]
Pseudomonas syringae	pv *maculicola* str ES4326	+	-	-	/	/
Pseudomonas syringae	pv *pisi* str PP1	+	-	-	/	/
Pseudomonas syringae	pv *syringae* Pss9097	+	-	-	/	/
Pseudomonas syringae	pv *syringae* CFBP4215	+	-	-	P	[68]

Pseudomonas syringae	pv *syringae* CFBP2118	+	-	-	P	[68]
Pseudomonas syringae	pv *syringae* B301D	+	-	-	P	[70]
Pseudomonas syringae	pv *syringae* B64	+	-	-	P	[71]
Pseudomonas syringae	pv *syringae* B728a	+	-	-	P	[72]
Pseudomonas syringae	pv *syringae* HS191	+	-	-	P	[70]
Pseudomonas syringae	pv *syringae* SM	+	-	-	/	/
Pseudomonas syringae	pv *tomato* B13-200	+	-	-	/	/
Pseudomonas syringae	pv *tomato* delta VI	+	-	-	/	/
Pseudomonas syringae	pv *tomato* delta IV IX	+	-	-	/	/
Pseudomonas syringae	pv *tomato* delta X	+	-	-	/	/
Pseudomonas syringae	pv *tomato* str DC3000	+	-	-	P	[73]
Pseudomonas syringae	UB303	+	-	-	/	/
Pseudomonas syringae	UMAF0158	+	-	-	P	[74]
Pseudomonas syringae	USA011	+	-	-	/	/
Pseudomonas taetrolens	NCTC10697	+	-	-	/	/
Pseudomonas taetrolens	NCTC8067	+	-	-	/	/
Pseudomonas thivervalensis	DSM 13194	-	+	+	/	/
Pseudomonas tolaasii	2192T	+	-	-	/	/
Pseudomonas trivialis	IHBB745	+	-	-	B	[75]
Pseudomonas umsongensis	GO16	+	-	-	/	/
Pseudomonas umsongensis	LMG 21317	+	-	-	/	/
Pseudomonas vancouverensis	LMG 20222	+	-	+	/	/
Pseudomonas versuta	L10 10	+	-	-	/	/
Pseudomonas viridiflava	CFBP 1590	+	-	-	P	[76]

References

1. Smith, E.F.; Bryan, M. Angular leaf spot of cucumbers. *Journal of Agricultural Research* **1915**, *5*, 465-476.
2. Jeong, H.; Kloepper, J.W.; Ryu, C.M. Genome sequences of *Pseudomonas amygdali* pv. *tabaci* strain ATCC 11528 and pv. *lachrymans* strain 98A-744. *Genome Announc* **2015**, *3*, doi:10.1128/genomeA.00683-15.
3. Ark, P.A.; Tompkins, C.M. Bacterial leaf blight of bird's nest fern. *Phytopathology* **1946**, *36*, 758-761.
4. Fang, Y.; Wu, L.; Chen, G.; Feng, G. Complete genome sequence of *Pseudomonas azotoformans* S4, a potential biocontrol bacterium. *J Biotechnol* **2016**, *227*, 25-26, doi:10.1016/j.jbiotec.2016.04.020.
5. Berry, C.; Fernando, W.D.; Loewen, P.C.; De Kievit, T.R. Lipopeptides are essential for *Pseudomonas* sp. DF41 biocontrol of *Sclerotinia sclerotiorum*. *Biological Control* **2010**, *55(3)*, 211-218.
6. Novinscak, A.; Gadkar, V.J.; Joly, D.L.; Filion, M. Complete genome sequence of *Pseudomonas brassicacearum* LBUM300, a disease-suppressive bacterium with antagonistic activity toward fungal, oomycete, and bacterial plant pathogens. *Genome Announc* **2016**, *4*, doi:10.1128/genomeA.01623-15.
7. Grosch, R.; Faltin, F.; Lottmann, J.; Kofoet, A.; Berg, G. Effectiveness of 3 antagonistic bacterial isolates to control *Rhizoctonia solani* Kühn on lettuce and potato. *Can J Microbiol* **2005**, *51*, 345-353, doi:10.1139/w05-002.
8. Nelkner, J.; Tejerizo, G.T.; Hassa, J.; Lin, T.W.; Witte, J.; Verwaaijen, B.; Winkler, A.; Bunk, B.; Sproer, C.; Overmann, J., et al. Genetic potential of the biocontrol agent *Pseudomonas brassicacearum* (formerly *P. trivialis*) 3Re2-7 unraveled by genome sequencing and mining, comparative genomics and transcriptomics. *Genes (Basel)* **2019**, *10*, doi:10.3390/genes10080601.

9. Ortet, P.; Barakat, M.; Lalaouna, D.; Fochesato, S.; Barbe, V.; Vacherie, B.; Santaella, C.; Heulin, T.; Achouak, W. Complete genome sequence of a beneficial plant root-associated bacterium, *Pseudomonas brassicacearum*. *J Bacteriol* **2011**, *193*, 3146, doi:10.1128/JB.00411-11.
10. Kaluzna, M.; Willems, A.; Pothier, J.F.; Ruinelli, M.; Sobiczewski, P.; Pulawska, J. *Pseudomonas cerasi* sp. nov. (non Griffin, 1911) isolated from diseased tissue of cherry. *Syst Appl Microbiol* **2016**, *39*, 370-377, doi:10.1016/j.syapm.2016.05.005.
11. Loewen, P.C.; Villenueva, J.; Fernando, W.G.; de Kievit, T. Genome sequence of *Pseudomonas chlororaphis* strain PA23. *Genome Announc* **2014**, *2*, doi:10.1128/genomeA.00689-14.
12. Calderon, C.E.; de Vicente, A.; Cazorla, F.M. Role of 2-hexyl, 5-propyl resorcinol production by *Pseudomonas chlororaphis* PCL1606 in the multitrophic interactions in the avocado rhizosphere during the biocontrol process. *FEMS Microbiol Ecol* **2014**, *89*, 20-31, doi:10.1111/1574-6941.12319.
13. Deng, P.; Wang, X.; Baird, S.M.; Lu, S.E. Complete genome of *Pseudomonas chlororaphis* strain UFB2, a soil bacterium with antibacterial activity against bacterial canker pathogen of tomato. *Stand Genomic Sci* **2015**, *10*, 117, doi:10.1186/s40793-015-0106-x.
14. Town, J.; Audy, P.; Boyetchko, S.M.; Dumonceaux, T.J. Genome sequence of *Pseudomonas chlororaphis* strain 189. *Genome Announc* **2016**, *4*, doi:10.1128/genomeA.00581-16.
15. Li, Z.; Li, X.; Zeng, Q.; Chen, M.; Liu, D.; Wang, J.; Shen, L.; Song, F. Genome sequence of *Pseudomonas chlororaphis* Lzh-T5, a plant growth-promoting rhizobacterium with antimicrobial activity. *Genome Announc* **2018**, *6*, doi:10.1128/genomeA.00328-18.
16. Mehnaz, S.; Baig, D.N.; Lazarovits, G. Genetic and phenotypic diversity of plant growth promoting rhizobacteria isolated from sugarcane plants growing in pakistan. *J Microbiol Biotechnol* **2010**, *20*, 1614-1623, doi:10.4014/jmb.1005.05014.
17. Hunziker, L.; Bonisch, D.; Groenhagen, U.; Bailly, A.; Schulz, S.; Weisskopf, L. *Pseudomonas* strains naturally associated with potato plants produce volatiles with high potential for inhibition of *Phytophthora infestans*. *Appl Environ Microbiol* **2015**, *81*, 821-830, doi:10.1128/AEM.02999-14.
18. Kim, M.S.; Kim, Y.C.; Cho, B.H. Gene expression analysis in cucumber leaves primed by root colonization with *Pseudomonas chlororaphis* O6 upon challenge-inoculation with *Corynespora cassiicola*. *Plant Biol (Stuttg)* **2004**, *6*, 105-108, doi:10.1055/s-2004-817803.
19. Zhang, L.; Chen, W.; Jiang, Q.; Fei, Z.; Xiao, M. Genome analysis of plant growth-promoting rhizobacterium *Pseudomonas chlororaphis* subsp. *aurantiaca* JD37 and insights from comparasion of genomics with three *Pseudomonas* strains. *Microbiol Res* **2020**, *237*, 126483, doi:10.1016/j.micres.2020.126483.
20. Morohoshi, T.; Yamaguchi, T.; Xie, X.; Wang, W.Z.; Takeuchi, K.; Someya, N. Complete genome sequence of *Pseudomonas chlororaphis* subsp. *aurantiaca* reveals a triplicate quorum-sensing mechanism for regulation of phenazine production. *Microbes Environ* **2017**, *32*, 47-53, doi:10.1264/jsme2.ME16162.
21. Puopolo, G.; Raio, A.; Pierson III, L.S.; Zoina, A. Selection of a new *Pseudomonas chlororaphis* strain for the biological control of *Fusarium oxysporum* f. sp. *radicis-lycopersici*. *Phytopathologia Mediterranea* **2011**, *50(2)*, 228-235.
22. Poštić, D.; Jošić, D.; Lepšanović, Z.; Aleksić, G.; Latković, D.; Starović, M. The effect of *Pseudomonas chlororaphis* subsp. *aurantiaca* strain Q16 able to inhibit *Fusarium oxysporum* growth on potato yield. *Ratarstvo i povrtarstvo* **2019**, *56(2)*, 41-48.
23. Mehnaz, S.; Bechthold, A.; Gross, H. Draft genome sequence of *Pseudomonas chlororaphis* subsp. *aurantiaca* ARS-38, a bacterial strain with plant growth promotion potential, isolated from the rhizosphere of cotton in Pakistan. *Microbiol Resour Announc* **2020**, *9*, doi:10.1128/MRA.01398-19.
24. Pierson, L.S., 3rd; Gaffney, T.; Lam, S.; Gong, F. Molecular analysis of genes encoding phenazine biosynthesis in the biological control bacterium *Pseudomonas aureofaciens* 30-84. *FEMS Microbiol Lett* **1995**, *134*, 299-307, doi:10.1111/j.1574-6968.1995.tb07954.x.
25. Nagendran, R.; Lee, Y.H. Green and red light reduces the disease severity by *Pseudomonas cichorii* JBC1 in tomato plants via upregulation of defense-related gene expression. *Phytopathology* **2015**, *105*, 412-418, doi:10.1094/PHYTO-04-14-0108-R.
26. Dutta, B.; Gitaitis, R.; Agarwal, G.; Coutinho, T.; Langston, D. *Pseudomonas coronafaciens* sp. nov., a new phytobacterial species diverse from *Pseudomonas syringae*. *PLoS One* **2018**, *13*, e0208271, doi:10.1371/journal.pone.0208271.
27. Zachow, C.; Muller, H.; Laireiter, C.M.; Tilcher, R.; Berg, G. Complete genome sequence of *Pseudomonas corrugata* strain RM1-1-4, a stress protecting agent from the rhizosphere of an oilseed rape bait plant. *Stand Genomic Sci* **2017**, *12*, 66, doi:10.1186/s40793-017-0278-7.
28. Sutra, L.; Siverio, F.; Lopez, M.M.; Hunault, G.; Bollet, C.; Gardan, L. Taxonomy of *Pseudomonas* strains isolated from tomato pith necrosis: emended description of *Pseudomonas corrugata* and proposal of three unnamed fluorescent *Pseudomonas* genomospecies. *Int J Syst Bacteriol* **1997**, *47*, 1020-1033, doi:10.1099/00207713-47-4-1020.
29. Yin, D.; Wang, N.; Xia, F.; Li, Q.; Wang, W. Impact of biocontrol agents *Pseudomonas fluorescens* 2P24 and CPF10 on the bacterial community in the cucumber rhizosphere. *European journal of soil biology* **2013**, *59*, 36-42.
30. Stritzler, M.; Diez Tissera, A.; Soto, G.; Ayub, N. Plant growth-promoting bacterium *Pseudomonas fluorescens* FR1 secrets a novel type of extracellular polyhydroxybutyrate polymerase involved in abiotic stress response in plants. *Biotechnol Lett* **2018**, *40*, 1419-1423, doi:10.1007/s10529-018-2576-6.
31. Ma, L.; Qu, S.; Lin, J.; Jia, J.; Baird, S.M.; Jiang, N.; Li, H.; Hou, L.; Lu, S.E. The complete genome of the antifungal bacterium *Pseudomonas* sp. strain MS82. *Journal of Plant Diseases and Protection* **2019**, *126(2)*, 153-160.
32. Dutta, S.; Yu, S.M.; Jeong, S.C.; Lee, Y.H. High-throughput analysis of genes involved in biocontrol performance of *Pseudomonas fluorescens* NBC275 against Gray mold. *J Appl Microbiol* **2020**, *128*, 265-279, doi:10.1111/jam.14475.
33. Novinscak, A.; Joly, D.L.; Filion, M. Complete genome sequence of the plant growth-promoting rhizobacterium *Pseudomonas fluorescens* LBUM677. *Microbiol Resour Announc* **2019**, *8*, doi:10.1128/MRA.00438-19.
34. P., S.; A., B.; F., O.G.; D., G.J. Isolation, trace enrichment and liquid-chromatographic analysis of diacetylphloroglucinol in culture and soil samples using UV and amperometric detection. *Journal of Chromatography A* **1992**, *606(2)*, 171-177.

35. Silby, M.W.; Cerdeno-Tarraga, A.M.; Vernikos, G.S.; Giddens, S.R.; Jackson, R.W.; Preston, G.M.; Zhang, X.X.; Moon, C.D.; Gehrig, S.M.; Godfrey, S.A., et al. Genomic and genetic analyses of diversity and plant interactions of *Pseudomonas fluorescens*. *Genome Biol* **2009**, *10*, R51, doi:10.1186/gb-2009-10-5-r51.
36. Bangera, M.G.; Thomashow, L.S. Characterization of a genomic locus required for synthesis of the antibiotic 2,4-diacetylphloroglucinol by the biological control agent *Pseudomonas fluorescens* Q2-87. *Mol Plant Microbe Interact* **1996**, *9*, 83-90, doi:10.1094/mpmi-9-0083.
37. Raaijmakers, J.M.; Weller, D.M. Exploiting the genetic diversity of *Pseudomonas* spp: characterization of superior colonizing *P. fluorescens* strain Q8r1-96. *Appl. Environ. Microbiol* **2001**, *67*, 2545-2554.
38. Naseby, D.C.; Way, J.A.; Bainton, N.J.; Lynch, J.M. Biocontrol of *Pythium* in the pea rhizosphere by antifungal metabolite producing and non-producing *Pseudomonas* strains. *J Appl Microbiol* **2001**, *90*, 421-429, doi:10.1046/j.1365-2672.2001.01260.x.
39. Zeigler, R.S.; Alvarez, E. Bacterial sheath brown rot of rice caused by *Pseudomonas fuscovaginae* in Latin America. *Plant Disease* **1987**, *71(7)*, 592-597.
40. Lin, H.; Hu, S.; Liu, R.; Chen, P.; Ge, C.; Zhu, B.; Guo, L. Genome sequence of *Pseudomonas koreensis* CRS05-R5, an antagonistic bacterium isolated from rice paddy field. *Front Microbiol* **2016**, *7*, 1756, doi:10.3389/fmicb.2016.01756.
41. Wu, L.; Xiao, W.; Chen, G.; Song, D.; Khaskheli, M.A.; Li, P.; Zhang, S.; Feng, G. Identification of *Pseudomonas mosselii* BS011 gene clusters required for suppression of Rice Blast Fungus *Magnaporthe oryzae*. *J Biotechnol* **2018**, *282*, 1-9, doi:10.1016/j.jbiotec.2018.04.016.
42. Zengerer, V.; Schmid, M.; Bieri, M.; Muller, D.C.; Remus-Emsermann, M.N.P.; Ahrens, C.H.; Pelludat, C. *Pseudomonas orientalis* F9: a potent antagonist against phytopathogens with phytotoxic effect in the apple flower. *Front Microbiol* **2018**, *9*, 145, doi:10.3389/fmicb.2018.00145.
43. Jaaffar, A.K.M.; Parejko, J.A.; Paulitz, T.C.; Weller, D.M.; Thomashow, L.S. Sensitivity of *Rhizoctonia* isolates to phenazine-1-carboxylic acid and biological control by phenazine-producing *Pseudomonas* spp. *Phytopathology* **2017**, *107*, 692-703, doi:10.1094/PHYTO-07-16-0257-R.
44. Uron, P.; Giachini, A.J.; Glick, B.R.; Rossi, M.J.; Nascimento, F.X. Near-complete genome sequence of *Pseudomonas palleroniana* MAB3, a beneficial 1-aminocyclopropane-1-carboxylate deaminase-producing bacterium able to promote the growth of mushrooms and plants. *Genome Announc* **2018**, *6*, doi:10.1128/genomeA.00242-18.
45. Liu, Q.; Zhang, Y.; Yu, N.; Bi, Z.; Zhu, A.; Zhan, X.; Wu, W.; Yu, P.; Chen, D.; Cheng, S., et al. Genome sequence of *Pseudomonas parafulva* CRS01-1, an antagonistic bacterium isolated from rice field. *J Biotechnol* **2015**, *206*, 89-90, doi:10.1016/j.jbiotec.2015.03.017.
46. Kakembo, D.; Lee, Y.H. Analysis of traits for biocontrol performance of *Pseudomonas parafulva* JBCS1880 against bacterial pustule in soybean plants. *Biological Control* **2019**, *134*, 72-81.
47. Huang, X.; Wang, Z.; Liu, Y.; Zhang, X. Complete genome sequence of *Pseudomonas protegens* H78, a plant growth-promoting rhizobacterium. *Genome Announc* **2017**, *5*, doi:10.1128/genomeA.00233-17.
48. Chang, L.; Li, Q.; Tong, Y.; Xu, J.; Zhang, Q. Identification of the biocontrol bacterial strain FD6 and antimicrobial study of this bacterium against tomato grey mould pathogen *Botrytis cinerea*. *Acta Phytophylacica Sinica* **2011**, *38(6)*, 487-492.
49. Lou, H.; Wang, X.; Chen, J.; Wang, B.; Wang, W. Transcriptomic response of *Ralstonia solanacearum* to antimicrobial *Pseudomonas fluorescens* SN15-2 metabolites. *Can J Microbiol* **2018**, *64*, 816-825, doi:10.1139/cjm-2018-0094.
50. Takeuchi, K.; Noda, N.; Someya, N. Complete genome sequence of the biocontrol strain *Pseudomonas protegens* Cab57 discovered in Japan reveals strain-specific diversity of this species. *PLoS One* **2014**, *9*, e93683, doi:10.1371/journal.pone.0093683.
51. Stutz, E.W.; Défago, G.; Kern, H. Naturally occurring fluorescent pseudomonads involved in suppression of black root rot of tobacco. *Phytopathology* **1986**, *76*, 181-185.
52. Howell, C.R.; Stipanovic, R.D. Control of *Rhizoctonia solani* on cotton seedlings with *Pseudomonas fluorescens* and with an antibiotic produced by the bacterium. *Phytopathology* **1979**, *69(5)*, 480-482.
53. Zhai, Y.; Shao, Z.; Cai, M.; Zheng, L.; Li, G.; Yu, Z.; Zhang, J. Cyclo(l-Pro(-)l-Leu) of *Pseudomonas putida* MCCC 1A00316 isolated from antarctic soil: identification and characterization of activity against *Meloidogyne incognita*. *Molecules* **2019**, *24*, doi:10.3390/molecules24040768.
54. Yu, S.M.; Lee, Y.H. Genes involved in nutrient competition by *Pseudomonas putida* JBC17 to suppress green mold in postharvest satsuma mandarin. *J Basic Microbiol* **2015**, *55*, 898-906, doi:10.1002/jobm.201400792.
55. Molina, L.; Bernal, P.; Udaondo, Z.; Segura, A.; Ramos, J.L. Complete genome sequence of a *Pseudomonas putida* clinical isolate, strain H8234. *Genome Announc* **2013**, *1*, doi:10.1128/genomeA.00496-13.
56. Taghavi, S.; Garafola, C.; Monchy, S.; Newman, L.; Hoffman, A.; Weyens, N.; Barac, T.; Vangronsveld, J.; van der Lelie, D. Genome survey and characterization of endophytic bacteria exhibiting a beneficial effect on growth and development of poplar trees. *Appl Environ Microbiol* **2009**, *75*, 748-757, doi:10.1128/AEM.02239-08.
57. Perez-Martinez, I.; Rodriguez-Moreno, L.; Matas, I.M.; Ramos, C. Strain selection and improvement of gene transfer for genetic manipulation of *Pseudomonas savastanoi* isolated from olive knots. *Res Microbiol* **2007**, *158*, 60-69, doi:10.1016/j.resmic.2006.09.008.
58. Pieterse, C.M.; Zamioudis, C.; Berendsen, R.L.; Weller, D.M.; Van Wees, S.C.; Bakker, P.A. Induced systemic resistance by beneficial microbes. *Annu Rev Phytopathol* **2014**, *52*, 347-375, doi:10.1146/annurev-phyto-082712-102340.
59. Kamilova, F.; Validov, S.; Azarova, T.; Mulders, I.; Lugtenberg, B. Enrichment for enhanced competitive plant root tip colonizers selects for a new class of biocontrol bacteria. *Environ Microbiol* **2005**, *7*, 1809-1817, doi:10.1111/j.1462-2920.2005.00889.x.
60. Arseneault, T.; Goyer, C.; Filion, M. *Pseudomonas fluorescens* LBUM223 increases potato yield and reduces common scab symptoms in the field. *Phytopathology* **2015**, *105*, 1311-1317, doi:10.1094/PHYTO-12-14-0358-R.

61. Hockett, K.L.; Nishimura, M.T.; Karlsrud, E.; Dougherty, K.; Baltrus, D.A. *Pseudomonas syringae* CC1557: a highly virulent strain with an unusually small type III effector repertoire that includes a novel effector. *Mol Plant Microbe Interact* **2014**, *27*, 923-932, doi:10.1094/MPMI-11-13-0354-R.
62. Firrao, G.; Torelli, E.; Polano, C.; Ferrante, P.; Ferrini, F.; Martini, M.; Marcelletti, S.; Scortichini, M.; Ermacora, P. Genomic structural variations affecting virulence during clonal expansion of *Pseudomonas syringae* pv. *actinidiae* biovar 3 in Europe. *Front Microbiol* **2018**, *9*, 656, doi:10.3389/fmicb.2018.00656.
63. Everett, K.R.; Taylor, R.K.; Romberg, M.K.; Rees-George, J.; Fullerton, R.A.; Vanneste, J.L.; Manning, M.A. First report of *Pseudomonas syringae* pv. *actinidiae* causing kiwifruit bacterial canker in New Zealand. *Australasian Plant Disease Notes* **2011**, *6(1)*, 67-71.
64. McCann, H.C.; Rikkerink, E.H.; Bertels, F.; Fiers, M.; Lu, A.; Rees-George, J.; Andersen, M.T.; Gleave, A.P.; Haubold, B.; Wohlers, M.W., et al. Genomic analysis of the Kiwifruit pathogen *Pseudomonas syringae* pv. *actinidiae* provides insight into the origins of an emergent plant disease. *PLoS Pathog* **2013**, *9*, e1003503, doi:10.1371/journal.ppat.1003503.
65. Takikawa, Y.; Serizawa, S.; Ichikawa, T.; Tsuyumu, S.; Goto, M. *Pseudomonas syringae* pv. *actinidiae* pv. nov. . *Japanese Journal of Phytopathology* **1989**, *55(4)*, 437-444.
66. Ho, J.; Taiaroa, G.; Butler, M.I.; Poulter, R.T.M. The genome sequence of M228, a chinese isolate of *Pseudomonas syringae* pv. *actinidiae*, illustrates insertion sequence element mobility. *Microbiol Resour Announc* **2019**, *8*, doi:10.1128/MRA.01427-18.
67. Ménard, M.; Sutra, L.; Luisetti, J.; Prunier, J.P.; Gardan, L. *Pseudomonas syringae* pv. *avii* (pv. nov.), the causal agent of bacterial canker of wild cherries (*Prunus avium*) in France. *European Journal of Plant Pathology* **2003**, *109(6)*, 565-576.
68. Ruinelli, M.; Blom, J.; Smits, T.H.M.; Pothier, J.F. Comparative genomics and pathogenicity potential of members of the *Pseudomonas syringae* species complex on *Prunus* spp. *BMC Genomics* **2019**, *20*, 172, doi:10.1186/s12864-019-5555-y.
69. Kong, J.; Jiang, H.; Li, B.; Zhao, W.; Li, Z.; Zhu, S. Complete genome sequence of *Pseudomonas syringae* pv. *lapsa* strain ATCC 10859, isolated from infected wheat. *Genome Announc* **2016**, *4*, doi:10.1128/genomeA.00024-16.
70. Ravindran, A.; Jalan, N.; Yuan, J.S.; Wang, N.; Gross, D.C. Comparative genomics of *Pseudomonas syringae* pv. *syringae* strains B301D and HS 191 and insights into intrapathovar traits associated with plant pathogenesis. *Microbiologyopen* **2015**, *4(4)*, 553-573.
71. Dudnik, A.; Dudler, R. High-quality draft genome sequence of *Pseudomonas syringae* pv. *syringae* strain SM, isolated from wheat. *Genome Announc* **2013**, *1*, doi:10.1128/genomeA.00610-13.
72. Vinatzer, B.A.; Teitzel, G.M.; Lee, M.W.; Jelenska, J.; Hotton, S.; Fairfax, K.; Jenrette, J.; Greenberg, J.T. The type III effector repertoire of *Pseudomonas syringae* pv. *syringae* B728a and its role in survival and disease on host and non-host plants. *Mol Microbiol* **2006**, *62*, 26-44, doi:10.1111/j.1365-2958.2006.05350.x.
73. Cuppels, D.A. Generation and characterization of Tn5 insertion mutations in *Pseudomonas syringae* pv. *tomato*. *Appl Environ Microbiol* **1986**, *51*, 323-327, doi:10.1128/AEM.51.2.323-327.1986.
74. Martinez-Garcia, P.M.; Rodriguez-Palenzuela, P.; Arrebola, E.; Carrion, V.J.; Gutierrez-Barranquero, J.A.; Perez-Garcia, A.; Ramos, C.; Cazorla, F.M.; de Vicente, A. Bioinformatics analysis of the complete genome sequence of the mango tree pathogen *Pseudomonas syringae* pv. *syringae* UMAF0158 reveals traits relevant to virulence and epiphytic lifestyle. *PLoS One* **2015**, *10*, e0136101, doi:10.1371/journal.pone.0136101.
75. Gulati, A.; Swarnkar, M.K.; Vyas, P.; Rahi, P.; Thakur, R.; Thakur, N.; Singh, A.K. Complete genome sequence of the rhizobacterium *Pseudomonas trivialis* strain IHBB745 with multiple plant growth-promoting activities and tolerance to desiccation and alkalinity. *Genome Announc* **2015**, *3*, doi:10.1128/genomeA.00943-15.
76. Ruinelli, M.; Blom, J.; Pothier, J.F. Complete genome sequence of *Pseudomonas viridiflava* CFBP 1590, isolated from diseased cherry in France. *Genome Announc* **2017**, *5*, doi:10.1128/genomeA.00662-17.
